# Optimizing Dry Eye Disease Management with Topical Anti-Inflammatory or Immunomodulatory Drugs Combined with Artificial Tears: A Systematic Review and Meta-Analysis of Clinical Trials

**DOI:** 10.3390/jcm15207801

**Published:** 2026-10-09

**Authors:** Eugenia Piragine, Sara Veneziano, Lorenzo Flori, Valentina Citi, Marco Sansò, Chiara Posarelli, Alma Martelli, Vincenzo Calderone, Michele Figus

**Affiliations:** 1 Department of Pharmacy, University of Pisa, via Bonanno 6, 56126 Pisa, Italy; eugenia.piragine@unipi.it (E.P.); sara.veneziano@phd.unipi.it (S.V.); valentina.citi@unipi.it (V.C.); alma.martelli@unipi.it (A.M.); 2 Departmental Faculty of Medicine, UniCamillus, Saint Camillus International University of Health Sciences, via di Sant’Alessandro, 8, 00131 Rome, Italy; lorenzo.flori@unicamillus.org; 3 School of Specialization in Hospital Pharmacy, University of Pisa, via Bonanno 6, 56126 Pisa, Italy; 4 Istituto Farmaco Biologico Sperimentale (IFBS), via Carducci 64/D, 56017 Pisa, Italy; m.sanso@ifbs.it; 5 Department of Surgical Medical Molecular Pathology and Critical Care Medicine, University of Pisa, via Paradisa 2, 56124 Pisa, Italy; chiara.posarelli@unipi.it (C.P.); michele.figus@unipi.it (M.F.); 6 Istituto di Ricovero e Cura a Carattere Scientifico (IRCCS) Fondazione G.B. Bietti per lo Studio e la Ricerca in Oftalmologia, via Santo Stefano Rotondo 6, 00184 Rome, Italy

**Keywords:** dry eye disease, artificial tears, anti-inflammatory drugs, immunomodulatory drugs, efficacy, safety, meta-analysis

## Abstract

**Background/Objectives**: Dry eye disease (DED) is an ocular condition that significantly impairs quality of life. In mild cases, it is commonly treated with artificial tears (ATs), while more advanced cases are managed with immunomodulatory and anti-inflammatory drugs. The aim of this systematic review and meta-analysis was to evaluate the efficacy and safety of topical anti-inflammatory drugs, immunomodulatory drugs, AT, and their combination in adults with DED. **Methods:** The protocol was registered in the PROSPERO database (CRD420251245079). Intervention studies were searched in MEDLINE and Scopus databases. Random-effects meta-analyses were performed with the R software (version 4.5.2). **Results:** A total of 42 studies were included in the meta-analysis of efficacy and 46 in the analysis of safety. Overall, ATs and anti-inflammatory drugs did not produce significant improvements in clinical outcomes compared with controls. Immunomodulatory drugs showed variable results, with inconsistent efficacy and an increased risk of ocular adverse events (AEs). In contrast, combination therapy with ATs significantly improved clinical outcomes, including tear production (mean difference, MD, mm: 1.96, 95% CI [0.58–3.34] for anti-inflammatory drugs and 2.73, 95% CI [0.93; 4.52] for immunomodulatory drugs) and tear break-up time (MD, s: 1.43, 95% CI [0.81; 2.05] for anti-inflammatory drugs and 1.41, 95% CI [0.68; 2.13] for immunomodulatory drugs), without increasing the risk of AEs. **Conclusions:** Topical combination therapy with anti-inflammatory or immunomodulatory drugs and ATs appears to provide clinical improvements in tear function and patient-reported outcomes in DED, without additional safety concerns compared with ATs alone. These findings support combination treatment as a promising therapeutic strategy.

## 1. Introduction

Dry eye disease (DED) is a multifactorial disorder characterized by disruption of tear film homeostasis. It can be categorized into three subtypes: aqueous-deficient DED (resulting from reduced tear secretion), evaporative DED (caused by increased tear evaporation), and a mixed form [1]. Major symptoms include visual disturbance, burning, redness, foreign body sensation, and stinging, which can substantially impair patients’ quality of life [2,3].

The global prevalence of DED ranges from 5% to 34% [4] and increases with age [5]. In recent years, its incidence has also risen among younger individuals, potentially due to the widespread use of electronic devices and contact lenses, which may contribute to ocular surface stress [6,7]. In addition to lifestyle and aging, other recognized risk factors include female sex, the use of certain medications (such as antihistamines or topical antiglaucoma therapies), genetic predisposition, infections, and environmental exposures [4,8,9]. In rare cases, DED may also occur secondary to systemic conditions, such as Sjögren’s syndrome [10].

All these factors predispose the ocular surface to subclinical and chronic inflammation by promoting the release of chemokines, matrix metalloproteinases, and pro-inflammatory cytokines [11]. Accordingly, patients with DED exhibit higher tear levels of inflammatory mediators compared with healthy controls [12]. Activation of the immune system further contributes to a self-perpetuating vicious cycle that drives the progression and persistence of DED. Specifically, tear film instability and increased tear osmolarity induce cellular stress and damage to the ocular surface epithelium, which triggers the release of inflammatory mediators and the activation of innate and adaptive immune responses. Inflammation, in turn, further damages the corneal and conjunctival epithelium and can impair the function of the lacrimal glands and Meibomian glands, thereby reducing tear secretion and/or increasing tear evaporation. These changes further destabilize the tear film and exacerbate hyperosmolarity, perpetuating ocular surface inflammation and tissue damage. This vicious cycle can ultimately lead to persistent ocular surface dysfunction, worsening of signs and symptoms, and progression of DED [13].

The clinical management of DED typically begins with the use of artificial tears, which contain agents such as cellulose derivatives, polyvinyl alcohol, hydroxypropyl guar, povidone, and hyaluronic acid. These products range from low-viscosity solutions to high-viscosity gels and are ideally preservative-free to minimize further irritation of the ocular surface. Artificial tears primarily exert a mechanical effect by enhancing tear film stability and reducing surface stress, but they do not exhibit direct pharmacological activity [14]. Although they are widely used to temporarily relieve symptoms and improve lubrication of the ocular surface, artificial tears primarily provide supportive treatment and do not directly act on the pathophysiological mechanisms underlying DED, including inflammation of the ocular surface. Furthermore, their effects may be transient, requiring frequent administration, and their efficacy may vary depending on the formulation and the patient’s individual characteristics [15,16,17]. Therefore, in some patients, artificial tears alone may not ensure adequate disease control, supporting the rationale for combining them with pharmacological agents targeting specific pathogenic mechanisms.

Topical therapeutic interventions include immunomodulatory and anti-inflammatory agents, as immune system activation and inflammation are key etiopathogenetic mechanisms in DED. Among immunomodulatory drugs, the calcineurin inhibitors cyclosporine A (CsA) and tacrolimus, as well as the integrin antagonist lifitegrast, can inhibit T cell infiltration and reduce the subsequent release of pro-inflammatory cytokines [13]. Previous meta-analyses have shown that CsA and lifitegrast improve both signs and symptoms in patients with DED [18,19,20]. However, these studies focused on specific formulations and did not comprehensively evaluate other immunomodulatory drugs, limiting the overall evidence regarding the efficacy of this therapeutic class.

Anti-inflammatory drugs, on the other hand, include corticosteroids and non-steroidal anti-inflammatory drugs (NSAIDs), which suppress inflammatory signaling by inhibiting the inflammatory cascade [21]. In addition, the P2Y2 receptor agonist diquafosol has been shown to reduce tear film inflammatory markers in patients with DED after 4 weeks of treatment [22], while the mucin secretagogue rebamipide reduced cytokine levels in vitro [23] and attenuated ocular inflammation in a rat model [24]. Despite the established use of anti-inflammatory agents in clinical practice, only one meta-analysis has evaluated the efficacy and safety of topical corticosteroids in patients with DED [25], highlighting a gap in the literature.

Finally, the topical combination of anti-inflammatory or immunomodulatory drugs with artificial tears remains poorly explored. Combining these treatments may provide complementary effects by simultaneously improving tear film homeostasis and targeting the underlying inflammatory mechanisms of DED. Moreover, artificial tears may potentially influence drug retention time on the ocular surface, although the clinical relevance of this effect remains to be established. However, no up-to-date clinical guidelines or meta-analyses have comprehensively evaluated the combined use of pharmacological agents with artificial tears. Therefore, the aim of this systematic review and meta-analysis is to synthesize the available clinical evidence on the efficacy and safety of topical anti-inflammatory and immunomodulatory drugs, artificial tears, or their combination in patients with DED, thereby providing evidence to support clinical decision-making.

## 2. Materials and Methods

The study protocol for this systematic review was registered in the PROSPERO database (CRD420251245079). The research question was formulated using the PICOS framework. The risk of bias was assessed using: i) the Cochrane Risk of Bias (RoB 2) tool (version of 22 August 2019; Cochrane, London, UK) for randomized controlled trials (RCTs); ii) the RoB 2 tool for crossover studies (version revised on 18 March 2021; Cochrane, London, UK); and iii) the ROBINS-I tool (2016; Cochrane, London, UK) for nonrandomized controlled trials (NRCTs). The results were reported in accordance with the Preferred Reporting Items for Systematic reviews and Meta-Analyses (PRISMA) guidelines.

### 2.1. Search Strategy

Studies published up to 14 February 2025 were searched in the MEDLINE (via PubMed) and Scopus databases. The search strategy included two terms combined using the Boolean operator “AND”: the first related to the disease, and the second to the available therapeutic strategies for DED (Supplementary Material S1).

### 2.2. Study Selection

The study selection process was conducted using the screening tool Rayyan web (Rayyan Systems, Inc., Cambridge, MA, USA, accessed in February 2025) [26]. After removal of duplicates, three authors (E.P., S.V., and L.F.) independently screened the titles and abstracts of all retrieved records. Each record was classified as either irrelevant or potentially eligible according to predefined inclusion and exclusion criteria. The full texts of potentially eligible articles were then retrieved or, when unavailable, requested directly from the authors. Two authors (E.P. and S.V.) assessed the full texts and excluded studies that did not meet the inclusion criteria. Any disagreements during the study selection process were resolved through discussion with a third author (V.C.), who independently reviewed the titles, abstracts, and full texts of the contested papers and made the final decision regarding eligibility.

### 2.3. Inclusion Criteria

The inclusion criteria were defined according to the PICOS framework. P (population): adult patients (≥18 years) with DED, regardless of gender, concomitant pathological conditions, disease severity, or etiology (primary or secondary). I (intervention): topical anti-inflammatory or immunomodulatory drugs, artificial tears, or combinations of drugs with artificial tears. C (comparator): saline solution, vehicle/placebo, no treatment, or artificial tears (for studies evaluating drugs or their combination with artificial tears). O (outcome): efficacy (primary outcome, defined as improvement in clinical signs of DED and in patient-reported quality of life) and safety (secondary outcome). S (study design): parallel or cross-over controlled clinical studies, either randomized or nonrandomized. For cross-over studies, only data from the first treatment period were extracted to minimize bias related to carryover effects and ensure homogeneity with parallel-group trials.

### 2.4. Exclusion Criteria

Single-arm trials, studies evaluating non-topical drugs, and articles comparing different artificial tears, different drugs, or the same drugs administered at different doses were excluded. Observational studies, post hoc analyses of previously published studies, abstracts, reviews, letters to the editor, technical papers, commentaries, case reports, and studies published in languages other than English were also excluded.

### 2.5. Data Extraction

For each study included in the systematic review, two authors (E.P. and S.V.) independently extracted the following information using a Microsoft Excel spreadsheet (version 2025; Microsoft Corporation, Redmond, WA, USA): (i) reference (first author and year of publication); (ii) study design; (iii) description of the study population; (iv) type of control; (v) type of intervention (name, dosage, and treatment duration); (vi) concomitant use of artificial tears (for studies on drugs); (vii) sample size; (viii) general characteristics of patients, including age and gender; (ix) duration of follow-up period; (x) efficacy outcomes (baseline and follow-up values for each group, together with the reported measures of variability); and (xi) safety outcomes. For efficacy outcomes, between-group effect estimates and their corresponding variances were subsequently calculated using R software (version 4.5.2) from the extracted data according to the procedures described in Section 2.7. For safety outcomes, the number of events and the total number of participants in each group were extracted and used to calculate the risk differences (RDs) between the groups. Any discrepancies were resolved through discussion with a third author (L.F.).

### 2.6. Risk of Bias Assessment

Two authors (S.V. and L.F.) independently assessed the methodological quality of the included studies. The risk of bias in RCTs and cross-over studies was evaluated using the RoB 2 tool [27]. This tool comprises five domains assessing potential biases arising from the randomization process, deviations from intended interventions, missing outcome data, measurement of the outcome, and selection of the reported result. Each domain was rated as “low risk of bias”, “some concerns”, or “high risk of bias”. The overall risk of bias for each study was defined as “high risk” if at least one domain was rated as “high risk”, as “low risk” if all domains were rated as “low risk”, or as “some concerns” in all other cases.

The risk of bias in NRCTs was assessed using the Cochrane ROBINS-I tool [28]. This tool comprises seven domains assessing potential biases due to confounding, selection of participants, classification of interventions, deviations from intended interventions, missing data, measurement of outcomes, and selection of the reported result. Each domain was rated as having “low risk”, “moderate risk”, “serious risk”, or “critical risk” of bias. The overall risk of bias for each study was classified as “serious” if at least one domain was rated as “serious”, “moderate” if at least one domain was rated as “moderate”, and “low” if all domains were rated as “low”.

### 2.7. Meta-Analysis

When at least three studies were sufficiently homogeneous in terms of outcome measures and reported a follow-up period of at least one week, a random-effects model meta-analysis was performed using R software, and forest plots were generated. Given that the systematic review included studies evaluating different types of interventions (i.e., topical anti-inflammatory and immunomodulatory drugs, artificial tears, or their combination), each therapeutic approach was analyzed separately. When multiple interventions were compared with the same control group, the comparisons were analyzed separately to avoid double-counting patients and overestimating the precision of the pooled effect estimate.

For continuous outcomes (i.e., Schirmer’s test, tear break-up time—TBUT, and ocular surface disease index—OSDI—score), changes from baseline to follow-up were expressed as mean ± standard deviation (SD) for both the intervention and control groups. When the standard error of the mean (SEM) was reported, the SD was calculated as the SEM multiplied by the square root of the sample size in each group [29]. When a 95% or 80% Confidence Interval (CI) was reported, the SD was derived using the method described in the Cochrane Handbook for Systematic Reviews of Interventions [29]. When the mean change was not reported, it was calculated by subtracting the baseline value from the follow-up value for both the intervention and control groups. The corresponding SD was imputed assuming a correlation coefficient of 0.8, as previously described [30]. A random-effects meta-analysis of mean differences (MDs) was performed using the “metacont ()” function to estimate the pooled MD and corresponding 95% CI between the intervention and control groups.

Dichotomous outcomes (i.e., adverse events—AEs) were expressed as the number of events relative to the total number of participants in each group. RDs with corresponding 95% CI between groups were estimated using the “metabin ()” function. This analysis was performed for overall AEs, treatment-related AEs, ocular AEs, and treatment-related ocular AEs. Outcomes not included in these categories were summarized separately by extracting the safety data into a Microsoft Excel spreadsheet (version 2025) and calculating the percentage of patients experiencing AEs in the intervention and control groups. Only between-group differences greater than 2.0% between groups were reported in the Results Section. The definition of AEs was based on that provided by the original authors.

In all meta-analyses, study-specific weights were calculated using the inverse variance method, while between-study variance (τ^2^) was estimated using the restricted maximum likelihood (REML) estimator. Statistical heterogeneity was quantified using the Higgins’ I^2^ statistic and interpreted according to the Cochrane guidelines as potentially not important (0–40%), moderate (30–60%), substantial (50–90%), or considerable (75–100%) [29]. Heterogeneity was also assessed using Cochrane’s Q test. For the effectiveness of combination therapy, potential sources of heterogeneity were further explored through stratified analyses according to follow-up duration and DED severity. Statistical significance was set at *p* < 0.05 for both within-group and between-group comparisons.

## 3. Results

### 3.1. Systematic Review

**Figure 1:**
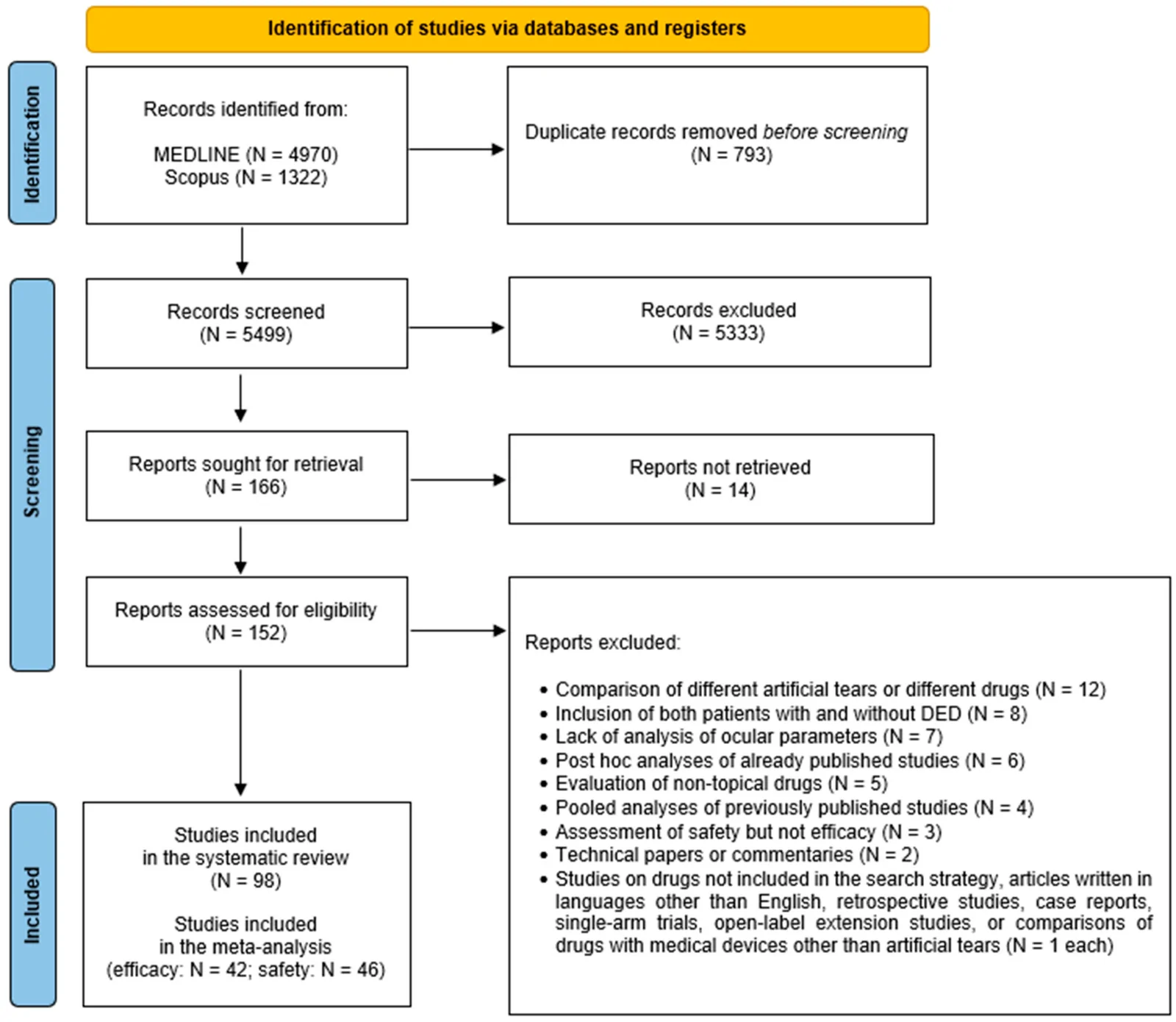
PRISMA flow diagram.

The search strategy identified 4970 records from MEDLINE (via PubMed) and 1322 from Scopus. After removal of duplicates (N = 793), a total of 5499 records were screened for eligibility. Of these, 5333 were excluded because they did not meet the inclusion criteria. The full texts of the remaining 166 studies were sought, but 14 could not be retrieved. Of the 152 studies assessed for eligibility, 54 were excluded for the following reasons: (i) comparison of different artificial tears or different drugs (N = 12); (ii) inclusion of both patients with and without DED (N = 8); (iii) lack of analysis of ocular parameters (N = 7); (iv) post hoc analyses of already published studies (N = 6); (v) evaluation of non-topical drugs (N = 5); (vi) pooled analyses of previously published studies (N = 4); (vii) assessment of safety but not efficacy (N = 3); (viii) technical papers or commentaries (N = 2); or (ix) studies on drugs not included in the search strategy, articles written in languages other than English, retrospective studies, case reports, single-arm trials, open-label extension studies, or comparisons of drugs with medical devices other than artificial tears (N = 1 each). At the end of the screening process, 98 studies were included in the systematic review, of which 42 were included in the efficacy meta-analysis and 46 in the safety of meta-analysis (Figure 1).

### 3.2. Characteristics of the Included Studies

The characteristics of the studies included in the systematic review are summarized in Table S1. Most studies were RCTs (N = 82) [31,32,33,34,35,36,37,38,39,40,41,42,43,44,45,46,47,48,49,50,51,52,53,54,55,56,57,58,59,60,61,62,63,64,65,66,67,68,69,70,71,72,73,74,75,76,77,78,79,80,81,82,83,84,85,86,87,88,89,90,91,92,93,94,95,96,97,98,99,100,101,102,103,104,105,106,107,108,109,110,111,112], 9 were NRCTs [113,114,115,116,117,118,119,120,121], and 7 were cross-over studies [122,123,124,125,126,127,128].

All studies included patients with DED. Some studies reported specific information regarding (i) disease severity (mild: N = 2 [117,126]; moderate: N = 3 [42,92,123]; mild to moderate: N = 5 [43,44,72,78,119]; moderate to severe: N = 20 [33,36,37,39,41,45,54,67,80,81,83,90,100,101,102,110,112,115,125,127]; severe: N = 4 [34,69,77,98]); (ii) DED features (short TBUT: N = 2 [62,64]; unstable tear film: N = 2 [84,99]; lipid-deficient DED: N = 1 [31]; aqueous-deficient DED: N = 1 [91]; delayed tear clearance: N = 1 [82]); and (iii) main causes or comorbidities, including Meibomian gland dysfunction (N = 6) [51,61,63,65,84,87], Sjögren’s syndrome (N = 5) [37,52,55,77,113], corneal/cataract surgery or laser in situ keratomileusis (N = 6) [32,47,59,76,89,108], contact lens use (N = 5) [58,60,74,109,116], chronic graft-versus-host disease (N = 3) [111,117,121], thyroid orbitopathy (N = 1) [34], allergic conjunctivitis (N = 1) [73], and trachoma (N = 1) [56]. The mean age (± SD) of participants was 54.4 ± 12.9 years in the intervention group and 53.3 ± 13.4 years in the control group, while the proportion of men was 23.7% and 24.7%, respectively.

Comparisons included artificial tears versus saline solution, seawater spray, vehicle, placebo, or no treatment (N = 15 study arms); anti-inflammatory or immunomodulatory drugs versus saline solution, vehicle, placebo, or no treatment (N = 35 study arms); drugs versus artificial tears (N = 28 study arms); and combinations of drugs with artificial tears versus artificial tears alone (N = 31 study arms). Regarding anti-inflammatory and immunomodulatory drugs, most study arms assessed CsA (N = 47), followed by diquafosol (N = 14), lifitegrast (N = 6), and the corticosteroids loteprednol etabonate or fluorometholone (both N = 4). Other drugs evaluated in fewer study arms are listed in Table S1. Observation periods ranged from 6 h [123] for acute interventions to 12 months [85,86,89] for chronic treatments.

### 3.3. Results of the Risk of Bias Assessment

**Figure 2:**
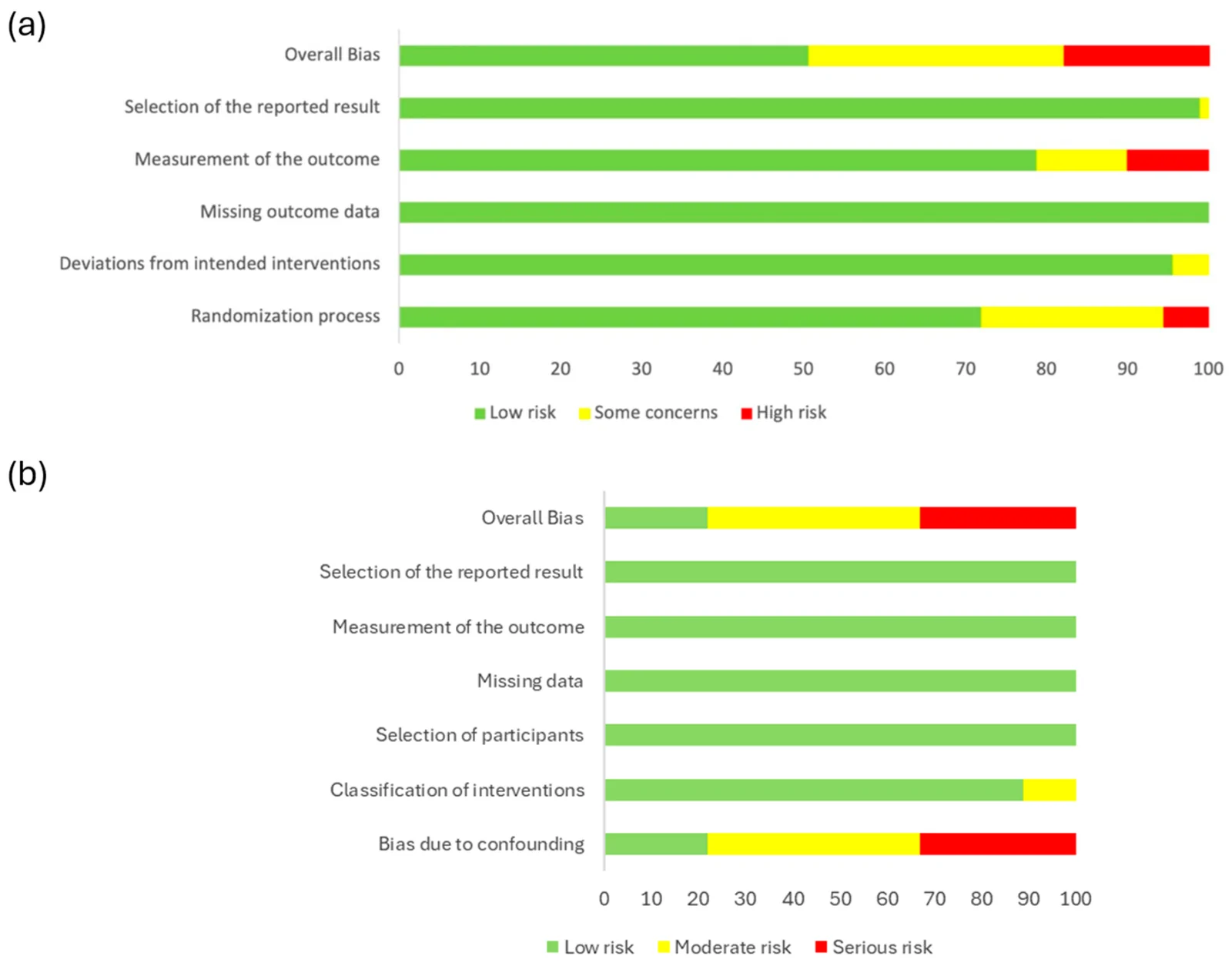
Summary of the risk of bias assessment of the RCTs (**a**) and NRCTs (**b**) included in the systematic review. Results are reported as percentages.

Among RCTs and cross-over studies, 45 (50.5%) were classified as having a “low” risk of bias [32,33,35,36,37,38,39,41,42,43,45,51,52,53,56,57,61,63,66,67,68,69,75,78,80,81,84,88,89,92,94,95,96,97,105,106,111,112,122,124,125,126,127,128], 28 (31.5%) as having “some concerns” [31,34,40,44,47,49,50,55,58,59,60,62,64,71,72,73,74,79,83,85,86,93,101,104,107,108,109,123], and 16 (18.0%) as having a “high” risk of bias [46,48,54,65,70,76,77,82,87,90,98,99,100,102,103,110] (Figure 2a). The main sources of potential bias were related to outcome measurement, particularly the use of non-standard or inadequately described methods, and to the randomization process.

Among NRCTs, two (22.0%) were classified as having a “low” risk of bias [114,120], four (45.0%) as having a “moderate” risk of bias [115,116,118,119], and three (33.0%) as having a “serious” risk of bias [113,117,121] (Figure 2b). The main factors contributing to the risk of bias were potential confounding factors, including differences in age (i.e., younger age, <50 years), sex distribution (e.g., a higher proportion of men, >50.0%), and concomitant pathological conditions that could worsen the severity of DED and potentially affect the study results.

### 3.4. Results of the Meta-Analysis

#### 3.4.1. Efficacy

##### Tear Production

**Figure 3:**
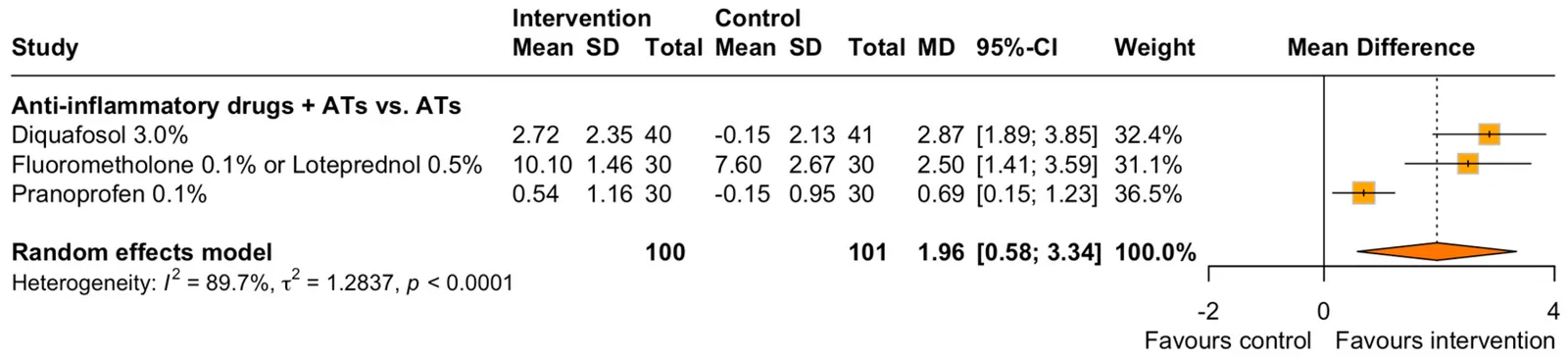
Forest plot of the mean difference (MD) with 95% Confidence Interval (CI) of Schirmer’s test results (mm) for the combination of anti-inflammatory drugs with artificial tears (ATs) vs. ATs.

Tear production was evaluated using Schirmer’s test in all the included studies. The meta-analysis of studies investigating artificial tears showed no significant effect on tear production compared with saline solution, seawater spray or no treatment (Figure S1 and Table 1). Similarly, the meta-analysis of studies investigating anti-inflammatory drugs showed no significant increase in tear production compared with saline solution or vehicle (Figure S2 and Table 1), or compared with artificial tears (Figure S3 and Table 1). This finding was confirmed in the sensitivity analysis excluding studies on diquafosol and rebamipide, which are not primarily classified as anti-inflammatory drugs. Notably, the combination of anti-inflammatory drugs with artificial tears significantly increased tear production compared with artificial tears alone (3 studies; MD, mm: 1.96, 95% CI [0.58; 3.34]; I^2^ = 89.7%) (Figure 3 and Table 1). This finding was no longer statistically significant in the sensitivity analysis excluding the study on diquafosol (MD, mm: 1.53, 95% CI [−0.24; 3.30]; I^2^ = 88.3%; Figure S4). However, this analysis was based on only two studies and therefore warrants confirmation. Finally, heterogeneity was no longer observed in the analysis stratified by follow-up duration, which showed a greater effect of the combination therapy in studies with a 12-week follow-up than in those with a 4-week follow-up (Table 2 and Figure S5).

**Figure 4:**
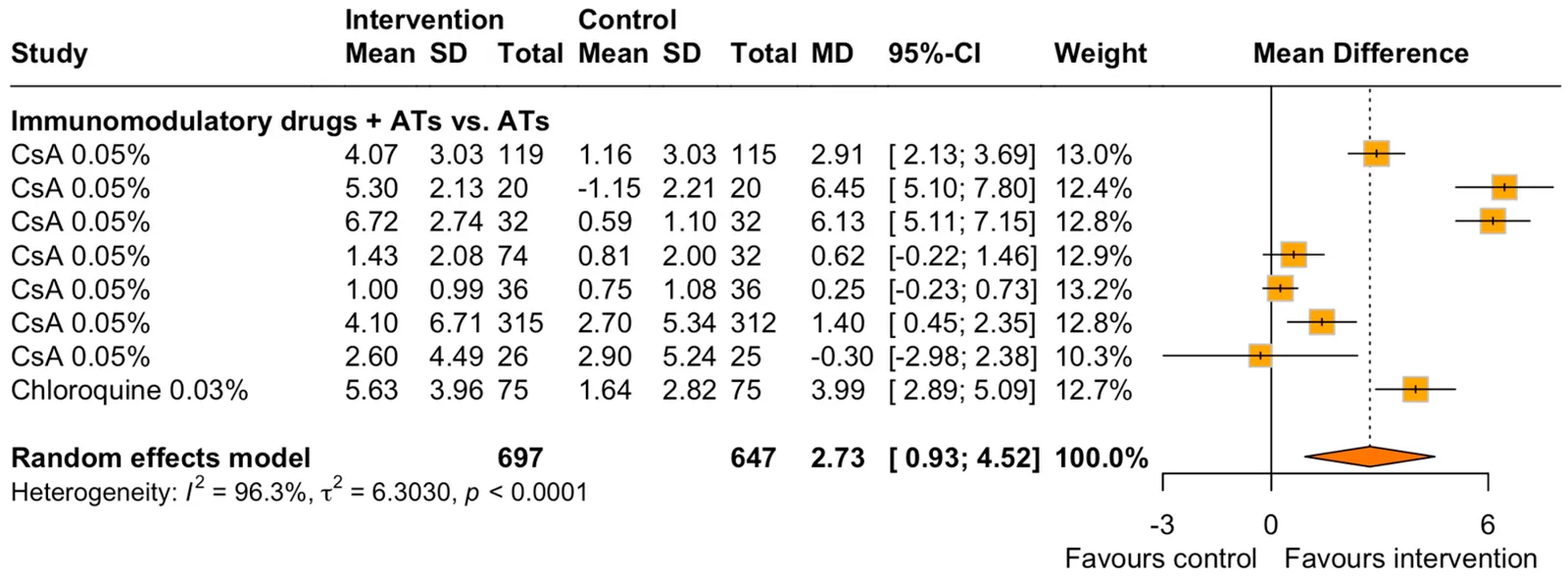
Forest plot of the mean difference (MD) with 95% Confidence Interval (CI) of Schirmer’s test results (mm) of the sensitivity analysis for the combination of immunomodulatory drugs with artificial tears (ATs) vs. ATs. Other abbreviations: CsA, cyclosporine.

The meta-analysis of studies investigating immunomodulatory drugs showed no significant effect on tear production compared with saline solution or vehicle (Figure S6 and Table 1), or compared with artificial tears (Figure S7 and Table 1). In addition, the combination of immunomodulatory drugs with artificial tears did not significantly increase tear production compared with artificial tears alone (9 studies; MD, mm: 2.03, 95% CI [−0.07; 4.12]) (Figure S8). One study was identified as a potential outlier [34]; therefore, a sensitivity analysis was performed by removing one study at a time. The results became statistically significant only when the study by Altiparmak and colleagues [34]—which reported findings in the opposite direction to those of all other studies—was excluded (MD, mm: 2.73; 95% CI [0.93; 4.52]; I^2^ = 96.3%) (Figure 4 and Table 1). Heterogeneity remained considerable for this comparison in the analyses stratified by follow-up duration and DED severity (Table 2 and Figures S9 and S10). Overall, the sensitivity analysis suggests a possible beneficial effect of combining immunomodulatory drugs with artificial tears in patients with DED.

##### Tear Film Stability

**Figure 5:**
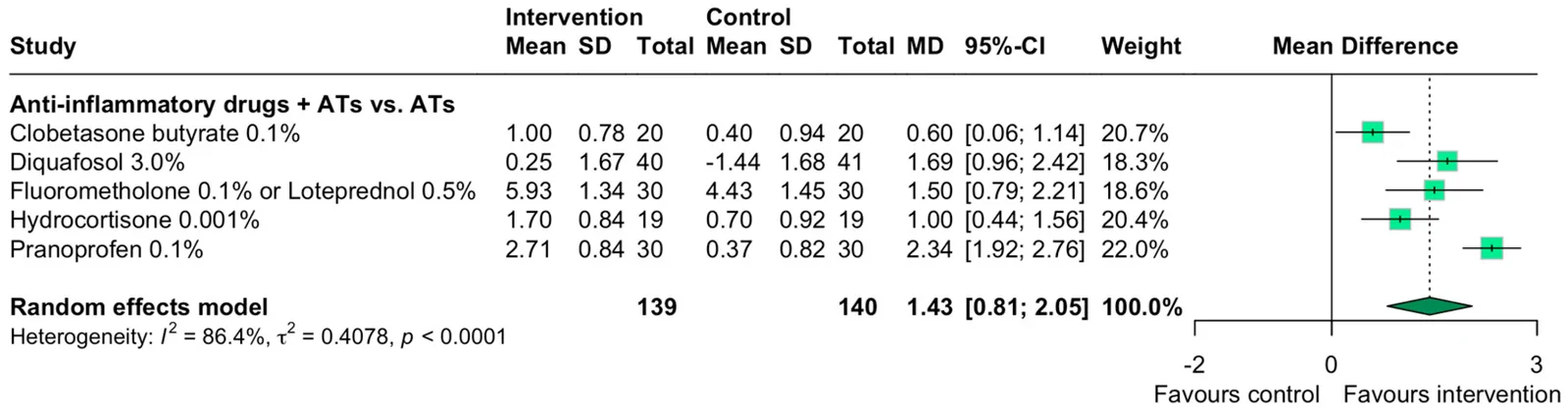
Forest plot of the mean difference (MD) with 95% Confidence Interval (CI) of TBUT results (s) for the combination of anti-inflammatory drugs with artificial tears (ATs) vs. ATs.

This parameter was assessed using TBUT in all the included studies. The meta-analysis of studies investigating artificial tears showed no significant effect on tear film stability compared with saline solution or seawater spray (Figure S11 and Table 3). In addition, anti-inflammatory drugs did not significantly improve this clinical parameter compared with artificial tears (Figure S12 and Table 3). This finding was confirmed in the sensitivity analysis excluding studies on diquafosol and rebamipide, which are not primarily classified as anti-inflammatory drugs. Conversely, the combination of anti-inflammatory drugs and artificial tears significantly increased tear film stability compared with artificial tears alone (5 studies; MD, s: 1.43, 95% CI [0.81; 2.05]; I^2^ = 86.4%) (Figure 5 and Table 3). This result was confirmed in the sensitivity analysis excluding the study on diquafosol (MD, s: 1.37, 95% CI [0.61; 2.13]; I^2^ = 89.8%; Figure S13). Heterogeneity was reduced in the analyses stratified by follow-up duration and DED severity, with combination therapy showing a significant effect after a 12-week follow-up, as well as in patients with unspecified DED severity, mild-to-moderate DED, or DED associated with Sjögren’s syndrome or Meibomian gland dysfunction (Table 4 and Figures S14 and S15).

**Figure 6:**
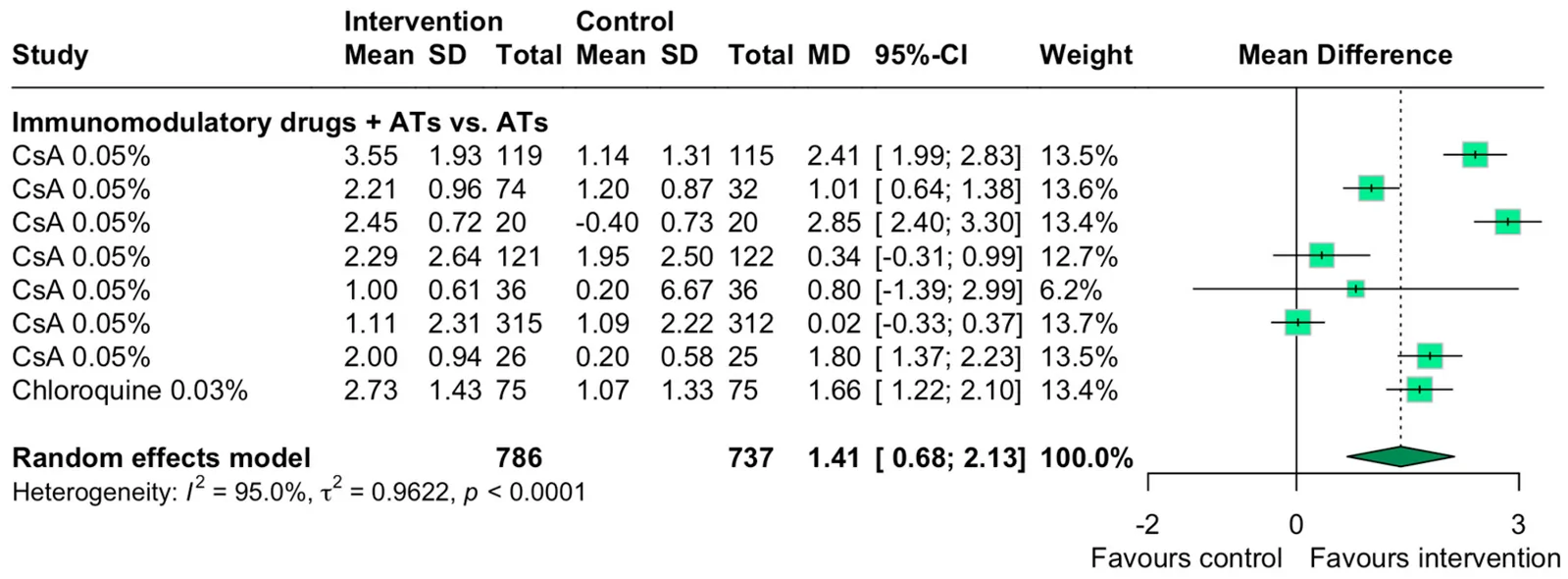
Forest plot of the mean difference (MD) with 95% Confidence Interval (CI) of TBUT results (s) for the combination of immunomodulatory drugs with artificial tears (ATs) vs. ATs. Other abbreviations: CsA, cyclosporine.

As concerns immunomodulatory drugs, the meta-analysis showed no significant effect on tear film stability compared with saline solution or vehicle (Figure S16 and Table 3). In contrast, the immunomodulatory agent CsA significantly increased this parameter compared with artificial tears (6 studies; MD, s: 1.19, 95% CI [0.13; 2.25]; I^2^ = 93.9%) (Figure S17 and Table 3). Finally, the combination of immunomodulatory drugs and artificial tears did not significantly improve tear film stability compared with artificial tears alone (10 studies; MD, s: 1.89, 95% CI [−0.09; 3.87]) (Figure S18a). The analysis was repeated by replacing one study arm from the study by Lee and colleagues [67] with the study arm assessing another combination therapy against the same control group, but the results remained non-significant (10 studies; MD, s: 1.84, 95% CI [−0.17; 3.84]; I^2^ = 98.5%) (Figure S18b). Two studies were identified as potential outliers [34,56]. As described in the Tear Production Section, a sensitivity analysis was therefore performed by removing one study at a time. The results became statistically significant when either the study by Altiparmak and colleagues [34] (MD, s: 2.33; 95% CI [0.35; 4.32]; I^2^ = 98.5%), or the study by Guzey and colleagues [56] (MD, s: 1.02; 95% CI [0.05; 1.99]; I^2^ = 95.6%), or both (MD, s: 1.41; 95% CI [0.68; 2.13]; I^2^ = 95.0%) were excluded (Figure 6 and Table 3). The findings were confirmed when the other available study arm from the study by Lee and colleagues [67] was included (Figure S19). Although heterogeneity remained considerable for this comparison in the analyses stratified by follow-up duration and DED severity (Table 4 and Figures S20 and S21), the sensitivity analyses suggest a possible beneficial effect of combining immunomodulatory drugs with artificial tears on tear film stability in patients with DED.

##### OSDI Score

**Figure 7:**
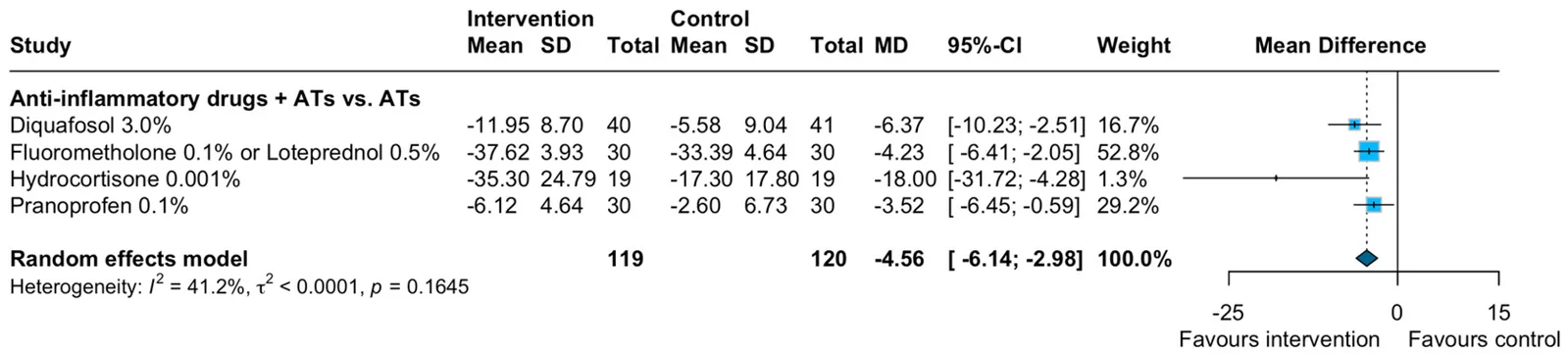
Forest plot of the mean difference (MD) with 95% Confidence Interval (CI) of OSDI scores for the combination of anti-inflammatory drugs with artificial tears (ATs) vs. ATs.

The OSDI questionnaire is commonly used to assess the impact of DED on patients’ quality of life [129]. The meta-analysis of studies investigating artificial tears showed no significant effect on OSDI scores compared with saline solution, seawater spray, or no treatment (Figure S22 and Table 5). Similarly, the meta-analysis of studies investigating anti-inflammatory drugs showed no significant reduction in OSDI scores compared with artificial tears (Figure S23 and Table 5). This finding was confirmed in the sensitivity analysis excluding the study on diquafosol, which is not primarily classified as an anti-inflammatory drug. As observed for tear production and tear film stability, the combination of anti-inflammatory drugs and artificial tears significantly reduced OSDI scores compared with artificial tears alone (4 studies; MD, score: −4.56, 95% CI [−6.14; −2.98]; I^2^ = 41.2%) (Figure 7 and Table 5). This result was confirmed in the sensitivity analysis excluding the study on diquafosol (MD, score: −4.20, 95% CI [−5.43; −2.47]; I^2^ = 51.1%; Figure S24).

**Figure 8:**
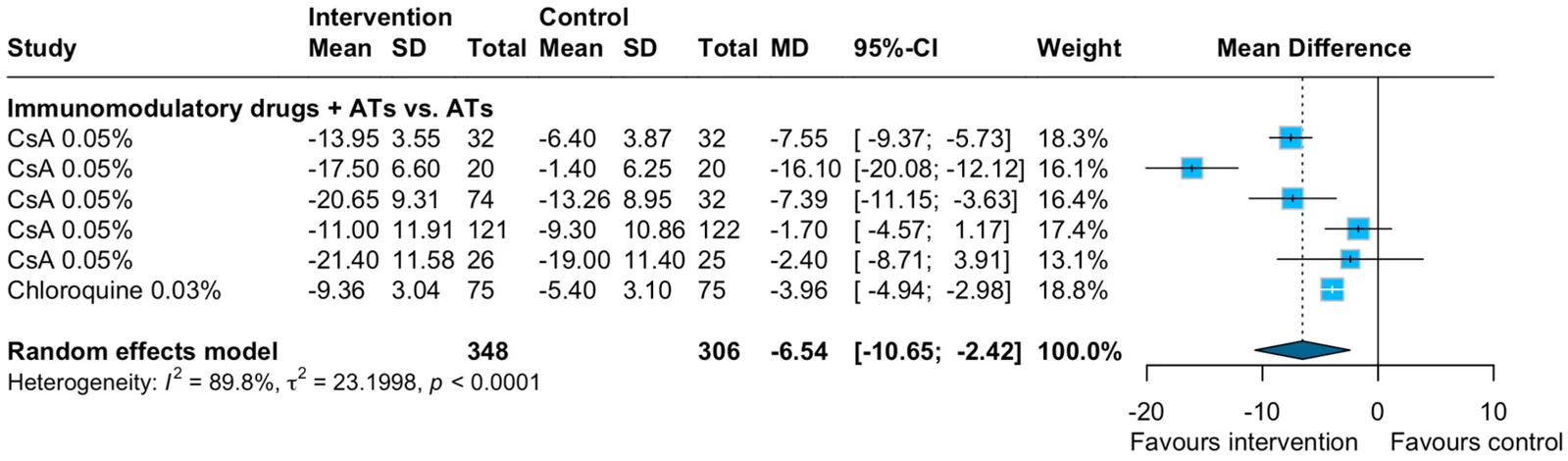
Forest plot of the mean difference (MD) with 95% Confidence Interval (CI) of OSDI scores for the combination of immunomodulatory drugs with artificial tears (ATs) vs. ATs.

The meta-analysis of studies investigating immunomodulatory drugs showed no significant effect on OSDI scores compared with vehicle or artificial tears (Figures S25 and S26 and Table 5). In contrast, the combination of immunomodulatory drugs and artificial tears significantly reduced OSDI scores compared with artificial tears alone (6 studies; MD, score: −6.54, 95% CI [−10.65; −2.42]; I^2^ = 89.8%) (Figure 8 and Table 5). In the analyses stratified by follow-up duration and DED severity, heterogeneity remained considerable (Table 6 and Figures S27 and S28). The results of the main analysis were confirmed when the other available study arm from the study by Lee and colleagues [67] was included (Figure S29). Overall, these findings suggest a potential beneficial effect of combining immunomodulatory drugs with artificial tears on quality of life in patients with DED.

#### 3.4.2. Safety

##### Any AEs

The meta-analysis of 6 studies showed that use of artificial tears did not significantly increase the risk of any AEs compared with saline solution or seawater spray (RD: 0.00, 95% CI [−0.03; 0.03]; I^2^ = 0.0%) (Figure S30). Similarly, no significant increase in the risk of any AEs was observed in patients receiving anti-inflammatory drugs compared with vehicle (5 studies; RD: 0.02, 95% CI [−0.03; 0.08]; I^2^ = 0.0%) or artificial tears (3 studies; RD: 0.00, 95% CI [−0.05; 0.05]; I^2^ = 0.0%) (Figures S31 and S32). This finding was confirmed in the sensitivity analysis excluding the study on diquafosol, which is not primarily classified as an anti-inflammatory drug. Finally, no significant increase in the risk of any AEs was observed in patients receiving a combination of anti-inflammatory drugs and artificial tears compared with artificial tears alone (3 studies; RD: 0.06, 95% CI [−0.10; 0.22]; I^2^ = 70.6%) (Figure S33). These findings were supported by the analyses of general and non-ocular AEs that were not included in the meta-analysis, which showed no increase in the percentage of patients experiencing AEs among those receiving anti-inflammatory drugs (Table S2) or those receiving anti-inflammatory drugs combined with artificial tears (Table S3) compared with controls.

Regarding immunomodulatory drugs, an 8-percentage-point increase was observed in the risk of any AEs compared with vehicle or placebo (12 studies; RD: 0.08, 95% CI [0.03; 0.14]; I^2^ = 82.5%) (Figure S34a), with lifitegrast being the main contributor to this result. Conversely, no significant increase in the risk of any AEs was observed for the combination of immunomodulatory drugs and artificial tears compared with artificial tears alone (6 studies; RD: 0.02, 95% CI [−0.02; 0.06]; I^2^ = 37.0%) (Figure S35a). These findings were confirmed in the analyses including the additional available study arm from the study by Liew and colleagues [70] (12 studies; RD: 0.07, 95% CI [0.01; 0.13]; I^2^ = 84.2%) (Figure S34b) and by Lee and colleagues [67] (6 studies; RD: 0.02, 95% CI [−0.02; 0.06]; I^2^ = 35.1%) (Figure S35b).

The analysis of general and non-ocular AEs not included in the meta-analysis showed higher percentages of patients receiving immunomodulatory drugs who experienced mild AEs (29.8% vs. 19.9%), moderate AEs (9.1% vs. 4.0%), non-ocular AEs (19.9% vs. 13.8%), mild non-ocular AEs (15.8% vs. 6.7%), moderate non-ocular AEs (7.4% vs. 3.1%), discontinuation due to AEs (4.3% vs. 2.0%), and withdrawal due to AEs (5.8% vs. 2.3%), or drug intolerance (11.1% vs. 0.0%) (Table S2), compared with the control group. Conversely, among patients receiving a combination of immunomodulatory drugs and artificial tears, higher percentages were observed only for clinical AEs (6.5% vs. 4.2%) and non-ocular AEs (18.6% vs. 13.9%) (Table S3).

*Any treatment-related AEs*

The use of artificial tears was not associated with a significantly higher risk of treatment-related AEs compared with saline solution or seawater spray (5 studies; RD: 0.00, 95% CI [−0.02; 0.02]; I^2^ = 0.0%) (Figure S36). Similarly, anti-inflammatory drugs did not significantly increase the risk of treatment-related AEs compared with vehicle (3 studies; RD: 0.00, 95% CI [−0.05; 0.05]; I^2^ = 0.0%) (Figure S37) or artificial tears (4 studies; RD: 0.00, 95% CI [−0.04; 0.04]; I^2^ = 0.0%) (Figure S38). Finally, no significant increase in the risk of treatment-related AEs was observed in patients receiving a combination of anti-inflammatory drugs and artificial tears compared with artificial tears alone (3 studies; RD: 0.00, 95% CI [−0.05; 0.05]; I^2^ = 0.0%) (Figure S39). These findings were supported by the analyses of general and non-ocular AEs not included in the meta-analysis, which showed no increase in the percentage of patients experiencing AEs among those receiving anti-inflammatory drugs (Table S2) or those receiving anti-inflammatory drugs combined with artificial tears (Table S3), compared with controls.

For immunomodulatory drugs, an 11-percentage-point increase in the risk of any treatment-related AEs was observed compared with vehicle or placebo (7 studies; RD: 0.11, 95% CI [0.02; 0.20]; I^2^ = 94.0%) (Figure S40), with lifitegrast being the main contributor to this result. No statistically significant increase in the risk of treatment-related AEs was observed for the combination of immunomodulatory drugs and artificial tears compared with artificial tears alone (4 studies; RD: 0.04, 95% CI [0.00; 0.08]; I^2^ = 72.5%) (Figure S41).

The analysis of general and non-ocular AEs not included in the meta-analysis showed higher percentages of patients receiving immunomodulatory drugs who experienced treatment-related non-ocular AEs (17.0% vs. 1.3%), treatment-related mild non-ocular AEs (10.9% vs. 1.4%), treatment-related moderate non-ocular AEs (7.8% vs. 0.3%), and withdrawal due to treatment-related AEs (3.5% vs. 0.5%) (Table S2), compared with the control group. Conversely, no increase in treatment-related AEs was observed among patients receiving a combination of immunomodulatory drugs and artificial tears (Table S3).

##### Ocular AEs

The meta-analysis of 4 studies showed that the use of artificial tears does not significantly increase the risk of any ocular AEs compared with saline solution or seawater spray (RD: 0.00, 95% CI [−0.03; 0.03]; I^2^ = 0.0%) (Figure S42). Similarly, no significant increase in the risk of ocular AEs was observed in patients receiving anti-inflammatory drugs compared with vehicle (3 studies; RD: 0.01, 95% CI [−0.05; 0.06]; I^2^ = 0.0%) (Figure S43). This finding was confirmed in the sensitivity analysis excluding the study on diquafosol, which is not primarily classified as an anti-inflammatory drug. Only one study was potentially eligible for the meta-analysis evaluating the safety of a combination of anti-inflammatory drugs and artificial tears compared with artificial tears alone, and it reported no ocular AEs in either group [42].

The analysis of ocular AEs not included in the meta-analysis showed higher rates of conjunctivitis/conjunctival redness (4.2% vs. 0.0%), eye pruritus (2.2% vs. 0.0%), burning/stinging (4.8% vs. 2.0%), and dry eye (2.2% vs. 0.0%) among patients receiving anti-inflammatory drugs compared with those receiving vehicle or saline solution (Table S4). The combination of anti-inflammatory drugs and artificial tears was more frequently associated with mild conjunctival hyperemia (4.5% vs. 1.5%) and photophobia (4.1% vs. 0.0%) than artificial tears alone (Table S5). However, these findings are based on a limited number of studies and therefore warrant confirmation.

The use of immunomodulatory drugs was associated with a 12-percentage-point increase in the risk of ocular AEs compared with vehicle or placebo (12 studies; RD: 0.12, 95% CI [0.05; 0.19]; I^2^ = 88.0%) (Figure S44a), with lifitegrast being the main contributor to this result. This finding was confirmed in the analysis including the additional available study arm from the study by Liew and colleagues [70] (12 studies; RD: 0.11, 95% CI [0.04; 0.18]; I^2^ = 88.4%) (Figure S44b). The combination of immunomodulatory drugs with artificial tears did not significantly increase the risk of ocular AEs compared with artificial tears alone (5 studies; RD: 0.02, 95% CI [−0.02; 0.05]; I^2^ = 0.0%) (Figure S45).

The analysis of ocular AEs not included in the meta-analysis revealed higher rates of ocular AEs among patients receiving immunomodulatory drugs than among those receiving vehicle or saline solution. The use of immunomodulatory drugs was associated with higher rates of severe ocular AEs (5.2% vs. 3.1%), moderate ocular symptoms (13.2% vs. 2.0%), moderate ocular AEs (7.1% vs. 3.5%), mild ocular AEs (33.5% vs. 16.7%), eye disorders (16.9% vs. 12.2%), discontinuation due to instillation site pain (2.4% vs. 0.0%), and withdrawal due to ocular AEs (4.6% vs. 1.0%). In addition, higher rates of instillation site-related AEs were reported, including pruritus (4.1% vs. 0.9%), discomfort (3.3% vs. 0.0%), reaction (9.2% vs. 2.1%), pain (11.5% vs. 2.6%), and irritation (16.1% vs. 2.8%). Discontinuation due to ocular AEs was also more common with immunomodulatory drugs than with artificial tears alone (5.1% vs. 0.0%) (Table S4). The combination of immunomodulatory drugs and artificial tears was more frequently associated with discontinuation due to intense burning sensation (5.6% vs. 0.0%), burning (4.4% vs. 0.9%), and ocular pain (6.1% vs. 1.4%) compared with artificial tears. No other differences in AEs between groups were reported (Table S5).

*Treatment-related ocular AEs*

The use of artificial tears did not significantly increase the risk of treatment-related ocular AEs compared with saline solution or seawater spray (4 studies; RD: 0.00, 95% CI [−0.03; 0.03]; I^2^ = 0.0%) (Figure S46). Anti-inflammatory drugs were investigated in fewer than three studies for each comparison and were therefore not included in the meta-analysis. Importantly, the available studies did not suggest an increased risk of treatment-related ocular AEs compared with vehicle [54,83] or artificial tears [88,107]. Finally, only one study was potentially eligible for the meta-analysis evaluating the safety of a combination of anti-inflammatory drugs and artificial tears compared with artificial tears alone, and it reported no treatment-related ocular AEs in either group [42].

The analysis of treatment-related ocular AEs not included in the meta-analysis showed that a slightly higher proportion of patients receiving anti-inflammatory agents experienced treatment-related blurred vision (3.8% vs. 0.0%), redness (5.7% vs. 1.9%), and ocular pain/eye pain (3.1% vs. 0.0%) compared with vehicle or saline solution, as well as treatment-related grittiness compared with artificial tears (2.5% vs. 0.0%) (Table S4). Interestingly, the combination of anti-inflammatory drugs and artificial tears was not associated with an increased risk of treatment-related ocular AEs (Table S5).

Regarding immunomodulatory drugs, the meta-analysis of 6 studies showed a 17-percentage-point increase in the risk of experiencing treatment-related ocular AEs compared with vehicle or placebo (RD: 0.17, 95% CI [0.11; 0.22]; I^2^ = 59.8%) (Figure S47a). This result was confirmed in the analysis including the additional available study arm from the study by Liew and colleagues [70] (6 studies; RD: 0.15, 95% CI [0.08; 0.21]; I^2^ = 78.0%) (Figure S47b). Notably, no statistically significant increase in the risk of treatment-related ocular AEs was observed in patients receiving a combination of immunomodulatory drugs and artificial tears compared with artificial tears alone (4 studies; RD: 0.04, 95% CI [−0.01; 0.08]; I^2^ = 73.6%) (Figure S48).

The analysis of treatment-related ocular AEs not included in the meta-analysis showed that a higher proportion of patients receiving immunomodulatory agents experienced mild (13.6% vs. 7.5%), moderate (6.1% vs. 2.0%) and severe (9.2% vs. 3.7%) treatment-related ocular AEs compared with those receiving vehicle or saline solution. Treatment-related ocular AEs with a higher incidence among patients receiving immunomodulatory drugs included blurred vision (16.4% vs. 8.9%), stinging (3.8% vs. 1.2%), burning (17.2% vs. 8.5%), ocular discomfort (29.0% vs. 15.3%), eye irritation (16.1% vs. 2.4%), ocular pain/eye pain (4.7% vs. 2.4%), increased lacrimation (8.7% vs. 1.5%), and instillation site pain (11.5% vs. 4.4%) (Table S4). In contrast, the combination of immunomodulatory drugs and artificial tears was not associated with an increased risk of treatment-related ocular AEs (Table S5).

## 4. Discussion

**Figure 9:**
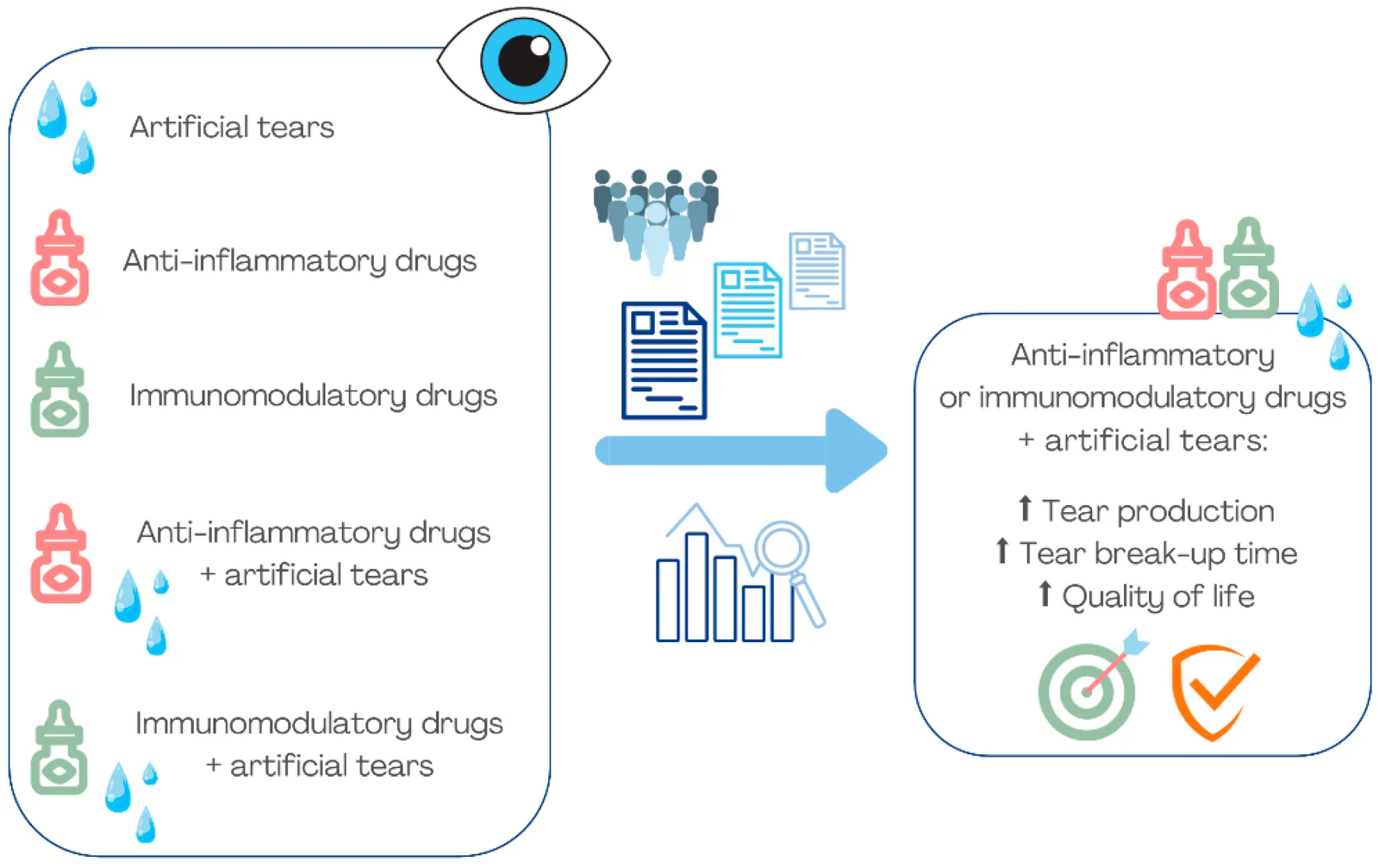
Summary of findings. Studies on the efficacy and safety of topical anti-inflammatory and immunomodulatory drugs, artificial tears or their combination in patients with DED were included in the systematic review with meta-analysis (left panel). The results showed that combining anti-inflammatory or immunomodulatory drugs with artificial tears significantly improve tear production, tear break-up time and quality of life, with an acceptable safety profile (right panel). These findings offer new insights into the clinical management of DED. Symbols: ↑ increase.

DED is a multifactorial syndrome that substantially affects patients’ quality of life [3] and requires complex, personalized, and patient-centered clinical management. Despite the availability of several therapeutic options, there is no universally standardized treatment approach, and evidence supporting the optimal therapeutic strategy remains limited [130,131]. This systematic review and meta-analysis aimed to provide a comprehensive overview of current topical treatments for DED, with a particular focus—examined here for the first time—on the combined use of anti-inflammatory or immunomodulatory drugs and artificial tears. The safety profiles of these interventions were also evaluated. The main findings are summarized in Figure 9 and discussed below.

First, the efficacy and safety of artificial tears were investigated. These products are generally recommended for patients with mild or episodic forms of DED or as adjunctive therapy in more complex cases [132,133]. However, in this meta-analysis, artificial tears did not significantly improved tear production, tear film stability, or patients’ quality of life compared with saline solution, seawater spray, or no treatment. These findings suggest that, although artificial tears remain an important component of symptomatic management, their effects on objective measures of tear production and tear film stability may be limited [14,16]. The safety profile was acceptable, with no increased risk of AEs reported in the included studies.

Topical anti-inflammatory drugs, which are recommended for patients with moderate to severe DED [133], did not significantly improve tear production, tear film break-up time, or patients’ quality of life compared with saline solution, placebo, or artificial tears. In the primary studies, anti-inflammatory agents included NSAIDs and corticosteroids, as well as drugs with more recently recognized anti-inflammatory mechanisms, such as diquafosol [22] and rebamipide [23,24]. Notably, the combination of anti-inflammatory drugs with artificial tears significantly improved all three clinical outcomes—particularly after 12 weeks of treatment—compared with artificial tears alone, supporting the potential benefit of combining anti-inflammatory drugs with artificial tears in patients with DED. The safety profile was favorable, with no increased incidence of any AEs or treatment-related AEs in the intervention group compared with controls. Regarding ocular AEs, patients receiving combination therapy showed a slight increase in mild conjunctival hyperemia and photophobia compared with those receiving artificial tears. Importantly, combination therapy was not associated with an increased risk of treatment-related ocular AEs. Overall, these findings offer new insights into the potential role of combination therapy in the clinical management of DED and provide a basis for future studies in this field.

Topical immunomodulatory drugs, such as CsA, are particularly recommended for patients with moderate DED and an inflammatory or autoimmune background who do not adequately respond to artificial tears [133,134]. In this meta-analysis, immunomodulatory drugs did not significantly improve tear production or patients’ quality of life compared with saline solution, vehicle, or artificial tears. Moreover, their effects on tear film stability were inconsistent, reaching statistical significance when compared with artificial tears but not when compared with saline solution or vehicle, and therefore remain inconclusive. Notably, the combination of immunomodulatory drugs with artificial tears significantly improved all three clinical outcomes compared with artificial tears alone. Although the results for Schirmer’s test and TBUT were obtained from sensitivity analyses excluding outlier studies [34,56], these findings suggest a potential benefit of this therapeutic strategy in patients with DED. The excluded studies involved specific patient populations, namely individuals with thyroid orbitopathy [34] and trachomatous dry eye [56], which may partially explain their divergent findings.

Immunomodulatory drugs are generally recommended for use under clinical supervision due to the potential risk of AEs [134]. In this meta-analysis, immunomodulatory agents were associated with a significantly higher risk of AEs (8.0%) and treatment-related AEs (11.0%) compared with vehicle or placebo. In contrast, no significant increase in these outcomes was observed among patients receiving a combination of immunomodulatory drugs and artificial tears, and combination therapy was not associated with an increased risk of discontinuations or withdrawals due to AEs. A similar pattern was observed for ocular AEs and treatment-related ocular AEs: monotherapy, but not combination therapy, was associated with an increased risk compared with the control group (12.0% for ocular AEs and 17.0% for treatment-related ocular AEs). Overall, these findings suggest that combination therapy is a safe option and support its consideration in clinical practice. Noteworthy, the results for AEs, treatment-related AEs, and ocular AEs (but not treatment-related ocular AEs) in the monotherapy group were largely influenced by lifitegrast, which was not included in the combination therapy analysis. Therefore, the apparently lower risk of AEs observed with combination therapy should not be interpreted as evidence that this approach is safer than monotherapy and should be interpreted with caution.

This study has some limitations. First, the exclusion of observational studies increased internal validity but prevented an assessment of the long-term effectiveness and safety of the interventions. Second, it was not possible to compare the efficacy of preservative-free formulations with that of formulations containing preservatives, which may have different effects on clinical outcomes, since very few studies have reported relevant data. Furthermore, the heterogeneity among the artificial tear formulations examined, combined with the low number of studies included in the meta-analysis, limits the generalizability of our results and warrants caution in their interpretation. Finally, the inclusion of zero events in the safety analyses, although necessary to provide a comprehensive assessment of the safety profile of the drugs under consideration, may have influenced the overall estimates. Further research is needed to overcome these limitations.

## 5. Conclusions

This is the first meta-analysis to demonstrate the efficacy and safety of anti-inflammatory or immunomodulatory drugs in combination with artificial tears in patients with DED, providing new insights for the clinical management of this multifactorial syndrome. Combination therapy may improve tear production, prolong tear film stability, and enhance quality of life compared with artificial tears alone, without an increased risk of AEs. These findings, although preliminary, provide a basis for future comparative studies of monotherapy and combination therapy, which are essential to establish whether the proposed therapeutic approach provides additional benefits in patients with DED.

In this context, the baseline DED phenotype is relevant when considering treatment response, as aqueous-deficient and evaporative DED are characterized by distinct predominant pathophysiological mechanisms and may therefore respond differently to combination therapy [132]. However, further studies are needed to determine whether the baseline phenotype can predict treatment response and guide the selection of combination therapy. In addition, the impact of combination therapy on treatment adherence should also be evaluated, as the use of multiple separate eye drops can negatively affect patient adherence [135]. This could be assessed by monitoring adherence or by evaluating combination products containing both artificial tears and the chosen medication, which could simplify dosing regimens. Finally, costs and accessibility should also be considered, as these factors may influence the feasibility and adoption of combination therapy in clinical practice.

## Figures and Tables

**Table 1:** Results of the meta-analyses on the efficacy of topical anti-inflammatory and immunomodulatory drugs, artificial tears or their combination on tear production, as assessed by Schirmer’s test. Abbreviations: ATs, artificial tears; CI, confidence interval; CsA, cyclosporine; MD: mean difference. Letters and symbols: ^a^ CsA; ^b^ CsA, tacrolimus, or tofacitinib; * results of the sensitivity analysis.

Intervention vs. Control	N. of Studies	MD (mm)	95% CI (mm)	I^2^ (%)
ATs vs. saline solution, seawater spray or no treatment	4	0.57	−0.04; 1.18	2.3
Anti-inflammatory drugs vs. saline solution or placebo	4	0.85	−0.02; 1.71	0.0
Anti-inflammatory drugs vs. ATs	5	−0.26	−0.63; 0.12	0.0
Anti-inflammatory drugs + ATs vs. ATs	3	1.96	0.58; 3.34	89.7
Immunomodulatory drugs vs. saline solution or vehicle	4 ^a,b^	1.63 ^a^ 0.75 ^b^	−0.34; 3.60 ^a^ −0.39; 1.90 ^b^	86.4 ^a^ 66.3 ^b^
Immunomodulatory drugs (CsA) vs. ATs	5	1.62	−0.60; 3.84	95.6
Immunomodulatory drugs + ATs vs. ATs *	8	2.73	0.93; 4.52	96.3

**Table 2:** Results of the meta-analyses on the efficacy of anti-inflammatory or immunomodulatory drugs combined with artificial tears, stratified by potential sources of heterogeneity, on tear production as assessed by Schirmer’s test. Abbreviations: ATs, artificial tears; CI, confidence interval; DED, dry eye disease; MD, mean difference; MGD, Meibomian gland dysfunction; SS, Sjögren’s syndrome.

Potential Source of Heterogeneity	N. of Studies	MD (mm)	95% CI (mm)	I^2^ (%)
Anti-inflammatory drugs + ATs vs. ATs				
Follow-up				
4 weeks	1	0.69	0.15; 1.23	-
12 weeks	2	2.70	1.98; 3.43	0.0
Immunomodulatory drugs + ATs vs. ATs				
Follow-up				
6–8 weeks	1	0.25	−0.23; 0.73	-
12 weeks	6	2.59	0.68; 4.50	92.8
24 weeks	1	6.13	5.11; 7.15	-
DED severity				
Mild to moderate	1	3.99	2.89; 5.09	-
Moderate to severe	4	2.65	0.16; 5.14	97.4
SS or MGD	3	2.33	−1.83; 6.49	96.4

**Table 3:** Results of the meta-analyses on the efficacy of topical anti-inflammatory and immunomodulatory drugs, artificial tears or their combination on tear film stability, as assessed by TBUT. Abbreviations: ATs, artificial tears; CI, confidence interval; CsA, cyclosporine; MD: mean difference. Letters and symbols: ^a^ CsA or lifitegrast; ^b^ CsA, lifitegrast, tacrolimus, or tofacitinib; * results of the sensitivity analysis.

Intervention vs. Control	N. of Studies	MD (s)	95% CI (s)	I^2^ (%)
ATs vs. saline solution or seawater spray	3	0.31	−0.16; 0.78	0.0
Anti-inflammatory drugs vs. ATs	8	0.58	−0.05; 1.20	88.3
Anti-inflammatory drugs + ATs vs. ATs	5	1.43	0.81; 2.05	86.4
Immunomodulatory drugs vs. saline solution or vehicle	6 ^a,b^	0.30 ^a^ 0.13 ^b^	−0.11; 0.72 ^a^ −0.10; 0.36 ^b^	58.9 ^a^ 17.6 ^b^
Immunomodulatory drugs (CsA) vs. ATs	6	1.19	0.13; 2.25	93.9
Immunomodulatory drugs + ATs vs. ATs *	8	1.41	0.68; 2.13	95.0

**Table 4:** Results of the meta-analyses on the efficacy of anti-inflammatory or immunomodulatory drugs combined with artificial tears, stratified by potential sources of heterogeneity, on tear film stability as assessed by TBUT. Abbreviations: ATs, artificial tears; CI, confidence interval; DED, dry eye disease; MD, mean difference; MGD, Meibomian gland dysfunction; SS, Sjögren’s syndrome.

Potential Source of Heterogeneity	N. of Studies	MD (s)	95% CI (s)	I^2^ (%)
Anti-inflammatory drugs + ATs vs. ATs				
Follow-up				
4 weeks	2	1.48	−0.23; 3.18	96.0
12 weeks	3	1.34	0.91; 1.78	19.7
DED severity				
Mild to moderate	2	1.30	0.63; 1.98	53.7
SS or MGD	1	0.60	0.06; 1.14	-
Unspecified	2	1.97	1.15; 2.79	75.1
Immunomodulatory drugs + ATs vs. ATs				
Follow-up				
6–8 weeks	1	0.80	−1.39; 2.99	-
12 weeks	7	1.45	0.67; 2.22	95.7
DED severity				
Mild to moderate	1	1.66	1.22; 2.10	-
Moderate to severe	4	0.91	−0.29; 2.11	96.0
SS or MGD	3	1.88	0.84; 2.93	94.8

**Table 5:** Results of the meta-analyses on the efficacy of topical anti-inflammatory and immunomodulatory drugs, artificial tears or their combination on quality of life, as assessed by OSDI questionnaire. Abbreviations: ATs, artificial tears; CI, confidence interval; CsA, cyclosporine; MD: mean difference. Letters: ^a^ carboxymethylcellulose (0.5% or not specified), hyaluronic acid (0.2%), or sodium hyaluronate (0.2% or 0.3%); ^b^ carboxymethylcellulose (0.5% or not specified), hyaluronic acid (0.2%), sodium hyaluronate (0.3%), or sodium hyaluronate (0.15%) + hydroxypropyl guar (0.175%); ^c^ CsA or lifitegrast; ^d^ CsA, lifitegrast, or tofacitinib; ^e^ CsA 0.05% + sodium hyaluronate (0.15%); ^f^ CsA 0.05% + carboxymethylcellulose (0.5%).

Intervention/Control	N. of Studies	MD (Score)	95% CI (Score)	I^2^ (%)
ATs vs. saline solution, seawater spray, or no treatment	5 ^a,b^	−4.72 ^a^ −4.48 ^b^	−12.40; 2.95 ^a^ −12.18; 3.23 ^b^	90.0 ^a^ 89.7 ^b^
Anti-inflammatory drugs vs. ATs	3	−3.28	−9.85; 3.29	68.8
Anti-inflammatory drugs + ATs vs. ATs	4	−4.56	−6.14; −2.98	41.2
Immunomodulatory drugs vs. vehicle	3 ^c,d^	−1.81 ^c^ −0.55 ^d^	−13.13; 9.51 ^c^ −4.38; 3.28 ^d^	69.9 ^c^ 51.6 ^d^
Immunomodulatory drugs (CsA) vs. ATs	4	−6.23	−14.02; 1.55	94.9
Immunomodulatory drugs + ATs vs. ATs	6 ^e,f^	−6.54 ^e^ −5.96 ^f^	−10.65; −2.42 ^e^ −10.74; −1.19 ^f^	89.8 ^e^ 92.5 ^f^

**Table 6:** Results of the meta-analyses on the efficacy of immunomodulatory drugs combined with artificial tears, stratified by potential sources of heterogeneity, on quality of life as assessed by OSDI questionnaire. Abbreviations: ATs, artificial tears; CI, confidence interval; DED, dry eye disease; MD, mean difference; MGD, Meibomian gland dysfunction; SS, Sjögren’s syndrome.

Potential Source of Heterogeneity	N. of Studies	MD (Score)	95% CI (Score)	I^2^ (%)
Immunomodulatory drugs + ATs vs. ATs				
Follow-up				
12 weeks	5	−6.32	−11.44; −1.19	90.0
24 weeks	1	−7.55	−9.37; −5.73	-
DED severity				
Mild to moderate	1	−3.96	−4.94; −2.98	-
Moderate to severe	2	−4.73	−10.46; 0.99	91.2
SS or MGD	3	−8.89	−16.64; −1.14	87.8

## Data Availability

Data are available upon request.

## Supplementary Materials

The following supporting information can be downloaded at https://www.mdpi.com/article/10.3390/jcm15207801/s1, S1: Search strategy, Table S1: Characteristics of the studies included in the systematic review, Figure S1: Forest plot of the mean difference (MD) with 95% Confidence Interval (CI) of Schirmer’s test results for artificial tears (ATs) vs. saline solution, seawater spray, or no treatment, Figure S2: Forest plot of the MD with 95% CI of Schirmer’s test results for anti-inflammatory drugs vs. saline solution or vehicle, Figure S3: Forest plot of the MD with 95% CI of Schirmer’s test results for anti-inflammatory drugs vs. ATs, Figure S4: Forest plot of the mean difference (MD) with 95% Confidence Interval (CI) of Schirmer’s test results (mm) for the combination of anti-inflammatory drugs and artificial tears (ATs) vs. ATs in the sensitivity analysis excluding the study on diquafosol, Figure S5: Forest plot of the MD with 95% CI of Schirmer’s test results for the combination of anti-inflammatory drugs with ATs vs. ATs, stratified by follow-up, Figure S6: Forest plot of the MD with 95% CI of Schirmer’s test results for immunomodulatory drugs vs. saline solution or vehicle, Figure S7: Forest plot of the MD with 95% CI of Schirmer’s test results for immunomodulatory drugs vs. AT, Figure S8: Forest plot of the MD with 95% CI of Schirmer’s test results for the combination of immunomodulatory drugs with ATs vs. ATs, Figure S9: Forest plot of the MD with 95% CI of Schirmer’s test results for the combination of immunomodulatory drugs with ATs vs. ATs, stratified by follow-up, Figure S10: Forest plot of the MD with 95% CI of Schirmer’s test results for the combination of immunomodulatory drugs with ATs vs. ATs, stratified by DED severity, Figure S11: Forest plot of the MD with 95% CI of tear break-up time (TBUT) results for ATs vs. saline solution or seawater spray, Figure S12: Forest plot of the MD with 95% CI of TBUT results for anti-inflammatory drugs vs. ATs, Figure S13: Forest plot of the mean difference (MD) with 95% Confidence Interval (CI) of TBUT results (s) for the combination of anti-inflammatory drugs and artificial tears (ATs) vs. ATs in the sensitivity analysis excluding the study on diquafosol, Figure S14: Forest plot of the MD with 95% CI of TBUT results for the combination of anti-inflammatory drugs with ATs vs. ATs, stratified by follow-up, Figure S15: Forest plot of the MD with 95% CI of TBUT results for the combination of anti-inflammatory drugs with ATs vs. ATs, stratified by DED severity, Figure S16: Forest plot of the MD with 95% CI of TBUT results for immunomodulatory drugs vs. saline solution or vehicle, Figure S17: Forest plot of the MD with 95% CI of TBUT results for immunomodulatory drugs vs. ATs, Figure S18: Forest plot of the MD with 95% CI of TBUT results for the combination of immunomodulatory drugs with ATs vs. ATs, Figure S19: Forest plot of the MD with 95% CI of TBUT results for the combination of immunomodulatory drugs with ATs vs. ATs, Figure S20: Forest plot of the MD with 95% CI of TBUT results for the combination of immunomodulatory drugs with ATs vs. ATs, stratified by follow-up, Figure S21: Forest plot of the MD with 95% CI of TBUT results for the combination of immunomodulatory drugs with ATs vs. ATs, stratified by DED severity, Figure S22: Forest plot of the MD with 95% CI of Ocular Surface Disease Index (OSDI) results for ATs vs. saline solution, seawater spray, or no treatment, Figure S23: Forest plot of the MD with 95% CI of OSDI scores for anti-inflammatory drugs vs. ATs, Figure S24: Forest plot of the mean difference (MD) with 95% Confidence Interval (CI) of Ocular Surface Disease Index (OSDI) results (score) for the combination of anti-inflammatory drugs and artificial tears (ATs) vs. ATs in the sensitivity analysis excluding the study on diquafosol, Figure S25: Forest plot of the MD with 95% CI of OSDI scores for immunomodulatory drugs vs. vehicle, Figure S26: Forest plot of the MD with 95% CI of OSDI scores for immunomodulatory drugs vs. AT, Figure S27: Forest plot of the MD with 95% CI of OSDI scores for the combination of immunomodulatory drugs with ATs vs. ATs, stratified by follow-up, Figure S28: Forest plot of the MD with 95% CI of OSDI scores for the combination of immunomodulatory drugs with ATs vs. ATs, stratified by DED severity, Figure S29: Forest plot of the MD with 95% CI of OSDI scores for the combination of immunomodulatory drugs with ATs vs. ATs, Figure S30: Forest plot of the risk difference (RD) with 95% CI for any adverse events (AEs) in studies comparing ATs with saline solution or seawater spray, Figure S31: Forest plot of the RD with 95% CI for any AEs in studies comparing anti-inflammatory drugs with vehicle, Figure S32: Forest plot of the RD with 95% CI for any AEs in studies comparing anti-inflammatory drugs with ATs, Figure S33: Forest plot of the RD with 95% CI for any AEs in studies comparing a combination of anti-inflammatory drugs and ATs with ATs alone, Figure S34: Forest plot of the RD with 95% CI for any AEs in studies comparing immunomodulatory drugs with vehicle or placebo, Figure S35: Forest plot of the RD with 95% CI for any AEs in studies comparing a combination of immunomodulatory drugs and ATs with ATs alone, Figure S36: Forest plot of the RD with 95% CI for treatment-related AEs in studies comparing ATs with saline solution or seawater spray, Figure S37: Forest plot of the RD with 95% CI for treatment-related AEs in studies comparing anti-inflammatory drugs with vehicle, Figure S38: Forest plot of the RD with 95% CI for treatment-related AEs in studies comparing anti-inflammatory drugs with AT, Figure S39: Forest plot of the RD with 95% CI for treatment-related AEs in studies comparing a combination of anti-inflammatory drugs and ATs with ATs alone, Figure S40: Forest plot of the RD with 95% CI for treatment-related AEs in studies comparing immunomodulatory drugs with vehicle or placebo, Figure S41: Forest plot of the RD with 95% CI for treatment-related AEs in studies comparing a combination of immunomodulatory drugs and ATs with ATs alone, Table S2: General and non-ocular AEs not included in the meta-analysis in patients treated with ATs, anti-inflammatory, or immunomodulatory drugs, Table S3: General and non-ocular AEs not included in the meta-analysis in patients treated with a combination of anti-inflammatory or immunomodulatory drugs and ATs, Figure S42: Forest plot of the RD with 95% CI for any ocular AEs in studies comparing ATs with saline solution or seawater spray, Figure S43: Forest plot of the RD with 95% CI for any ocular AEs in studies comparing anti-inflammatory drugs with vehicle, Table S4: Ocular AEs not included in the meta-analysis in patients treated with ATs, anti-inflammatory, or immunomodulatory drugs, Table S5: Ocular AEs not included in the meta-analysis in patients treated with a combination of anti-inflammatory or immunomodulatory drugs and AT, Figure S44: Forest plot of the RD with 95% CI for any ocular AEs in studies comparing immunomodulatory drugs with vehicle or placebo, Figure S45: Forest plot of the RD with 95% CI for any ocular AEs in studies comparing a combination of immunomodulatory drugs and ATs with ATs alone, Figure S46: Forest plot of the RD with 95% CI for treatment-related ocular AEs in studies comparing ATs with saline solution or seawater spray, Figure S47: Forest plot of the RD with 95% CI for treatment-related ocular AEs in studies comparing immunomodulatory drugs with vehicle or placebo, Figure S48: Forest plot of the RD with 95% CI for treatment-related ocular AEs in studies comparing a combination of immunomodulatory drugs and ATs with ATs alone.
