# Supplementary material for: Optimizing Dry Eye Disease Management with Topical Anti-Inflammatory or Immunomodulatory Drugs Combined with Artificial Tears: A Systematic Review and Meta-Analysis of Clinical Trials
Source: J Clin Med. 2026 Oct 9;15(20):7801. doi: 10.3390/jcm15207801 (PMC13705430; doi:10.3390/jcm15207801)
Supplement: Supplementary file 1 [file jcm-15-07801-s001.zip › jcm-4535689-supplementary.pdf]

S1. Search strategy.

MEDLINE (via PubMed)

(dry eye syndrome OR Sjögren's syndrome OR keratoconjunctivitis sicca OR keratitis sicca OR sicca syndrome OR xerophthalmia OR dry eye disease OR ocular surface disease OR dysfunctional tear syndrome OR dry eye*) AND (anti-inflammatory drug* OR ciclosporin A OR N-acetylcysteine OR NAC OR cysteine OR corticosteroid* OR NSAID* OR non-steroidal anti-inflammatory drug* OR diclofenac OR ketorolac OR lifitegrast OR artificial tear* OR anakinra OR pranoprofen OR tanfanercept OR isunakinra OR prednisolone OR loteprednol etabonate OR fluorometholone)

Scopus

TITLE-ABS-KEY ((dry AND eye AND syndrome) OR (Sjögren's AND syndrome) OR (keratoconjunctivitis AND sicca) OR (keratitis AND sicca) OR (sicca AND syndrome) OR xerophthalmia OR (dry AND eye AND disease) OR (ocular AND surface AND disease) OR (dysfunctional AND tear AND syndrome) OR (dry AND eye*)) AND TITLE-ABS-KEY ((anti-inflammatory AND drug*) OR ciclosporin A OR N-acetylcysteine OR NAC OR cysteine OR corticosteroid* OR NSAID* OR (non-steroidal AND anti-inflammatory AND drug*) OR diclofenac OR ketorolac OR lifitegrast OR (artificial AND tear*) OR anakinra OR pranoprofen OR tanfanercept OR isunakinra OR prednisolone OR (loteprednol AND etabonate) OR fluorometholone)

First author, year	Study design	Study population	Intervention	Control	Dosage frequency	Age, years (mean ± SD or median and range IQR), intervention/ control or total	Men (%), intervention/ control or total	N. of patients or eyes (intervention/ control)	Follow-up	Meta-analysis efficacy/ safety
Absolon, 1968	Randomized cross-over study	Patients with KCS	Acetilcysteine solution 20.0%	AT (composition unknown)	-	64.0 (43.0-77.0)	4.0	13 (period I), 15 (period II) / 17 (period I), 12 (period II)	-	No/No
Aguilar, 2014	RCT	Patients with lipid-deficient dry eye	AT (Systane® Balance ocular emulsion; propylene glycol 0.6%, hydroxypropyl-guar, borate, sorbitol, dimyristoylphosphati-dylglycerol and mineral oil)	Saline solution (Larmabak®; NaCl 0.9%)	4 times/day (both)	46.0 ± 20.0/ 43.0 ± 18.0	28.0/37.5	25/24	4 weeks	No/Yes
Ahmadi, 2024	RCT	Patients with DED after cataract surgery	CsA 0.05% (Lacrosporin®)	AT (TearLose®; HMC 0.3 g and dextran 0.1 g)	4 times/day (both)	64.2 ± 9.2	47.0	30/30	4 weeks	No/No
Akpek, 2023	RCT	Patients with moderate to severe DED	CsA 0.1% solution (CyclASol®)§	Vehicle§	2 times/day (both)	57.6 ± 15.4/ 56.6 ± 16.3	27.7/26.3	423/411	29 days	No/Yes
Altiparmak, 2010	RCT	Patients with severe DED and thyroid orbitopathy	CsA 0.05% emulsion (Restasis®) + AT (Refresh Tears®; HMC and dextran 0.1 g)	AT (Refresh Tears®; HMC and dextran 0.1 g)	• Drug: 2 times/day<br>• AT: 4 times/day	40.9 ± 8.8/ 41.0 ± 1.1	16.0/16.6	25/48*	Mean: 6 months	Yes/No
Amparo, 2013	RCT	Patients with refractory DED	Anakinra 2.5 or 5.0% solution	AT (Refresh Liquigel®; CMC 1.0%)	3 times/day (both)	52.0 ± 14.7	37.8/47.0	45/30	16 weeks (12 weeks + 4 weeks after stop)	Yes/No
Aragona, 2002	RCT	Patients with moderate to severe dry eye	AT (NaHA 0.15%)	Saline solution (NaCl 0.9%)	4-8 times/day (both)	50.2 ± 15.1/ 50.7 ± 15.0	21.0/20.0	19/25	3 months	No/Yes
Aragona, 2013	RCT	Patients with SS and moderate to severe DED	Clobetasone butyrate 0.1% suspension (Visucloben®) + AT (Wet-Comod®; PVP 2.0%)	AT (Wet-Comod®; PVP 2.0%) + vehicle	• Drug/vehicle: 2 times/day<br>• AT: 5-8 times/day	58.8 ± 6.7/ 60.1 ± 5.1	5.0/5.0	20/20	30 days	Yes/No
Avunduk, 2003	RCT	Patients with KCS	Flurbiprofen 0.03% solution (Ocufen®) or fluorometholone 0.1% suspension (Allergan®) + AT (Refresh®; CMC 0.5%)	AT (Refresh®; CMC 0.5%)	• Drugs: 4 times/day<br>• AT: 4-8 times/day	52.7 ± 11.0/ 51.2 ± 12.4	40.0/37.5	20/8	30 days	No/Yes
Baiza-Duran, 2010	RCT	Patients with moderate to severe DED	CsA 1.0% or 0.05% solution + AT (methylcellulose)	Vehicle + AT (methylcellulose)	• Drug/vehicle: 2 times/day<br>• AT: at least 8 times/day	57.0 ± 13.0	39.0	183 (in total)	14 weeks	No/No
Barabino, 2022	RCT	Patients with DED	Loteprednol etabonate 0.5% suspension (Lotemax®)^	Saline solution (Hydrabak®; NaCl 0.9%)^	Twice daily for 14 days, once a day for the next 14 days, and twice weekly for 28 days (both)	30-69	-/-	10/10	56 days	Yes/No
Baudouin, 2017	RCT	Patients with moderate to severe DED	CsA emulsion 0.1%#	Vehicle#	Once daily (both)	57.6 ± 12.9/ 58.8 ± 12.7	14.9/16.1	241/248	6 months	Yes/Yes
Borroni, 2023	RCT	Patients with moderate DED	Hydrocortisone 0.001% + AT (NaHA 0.2%)	AT (NaHA 0.15%) + vehicle (trehalose 3.0%)	4 times/day (both)	59.6 ± 7.5/ 60.2 ± 8.1	36.8/26.3	19/19	3 months	Yes/Yes
Chen, 2010	RCT	Patients with mild to moderate DED	CsA 0.05% emulsion#	Vehicle#	2 times/day (both)	46.6 ± 11.1/ 46.0 ± 12.1	28.5/24.5	116/117	8 weeks	Yes/Yes
Chen, 2014	RCT	Patients with mild to moderate DED	Pranoprofen 0.1% + AT (NaHA 0.1%)	AT (NaHA 0.1%)	3 times/day (both)	-/-	25.5/21.7	55/60	28 days	No/Yes
Chen, 2019	RCT	Patients with moderate to severe DED	CsA 0.05% emulsion + AT (hypomellose)	Placebo (vehicle) + AT (hypomellose)	• Drug/vehicle: 2 times/day<br>• AT: 3 times/day	46.3 ± 12.5/ 45.0 ± 12.4	18.5/22.6	119/115	12 weeks	Yes/Yes
Chun, 2018	RCT	Patients with DED undergoing allo-HSCT	CsA 0.05% emulsion (Restasis®)^	No treatment^	4 times/day	40 (16.0-61.0)/ 50.5 (25.0-64.0)	64.3/53.3	28/30	3 months	No/No
Chung, 2013	RCT	Patients with DED after cataract surgery	CsA 0.05% emulsion (Restasis®)^	Saline solution (NaCl 0.9%)^	2 times/day (both)	68.3 ± 6.0	-/-	32/32*	3 months	No/No

Demiryay, 2011	RCT	Patients with DED	CsA 0.05% emulsion (Restasis®) + AT (Tears Naturale® Free; HMC 0.3% and dextran 0.1%)	AT (Tears Naturale® Free; HMC 0.3% and dextran 0.1%)	• Drug: 2 times/day<br>• AT: 4 times/day	46.6 ± 12.3/ 44.3 ± 14.4	9.0/0.0	22/20	4 months	No/Yes
Deveci, 2014	NRCT	Patients with SS and DED	CsA 0.05% emulsion (Restasis®)^	Saline solution (NaCl 0.9%)^	2 times/day (both)	47.5 ± 10.2/ 45.3	26.9/-	26/20	1 month	No/No
Diaz-Llopis, 2019	RCT	Patients with DED	AT (Viscofresh®; CMC 0.5%)	Seawater spray (Quinton®)	5 times/day (both)	66.8 ± 8.4/ 68.1 ± 6.3	-/-	60/60	12 weeks	Yes/Yes
Dong, 2022	RCT	Patients with DED	Tanfanercept 0.25% solution^	Placebo (vehicle)^	2 times/day (both)	42.2 ± 9.8/ 43.1 ± 10.8	40.0/46.0	50/50	8 weeks	Yes/Yes
Fukuoka, 2019	RCT	Patients with dry eye and MGD	Diquafosol 3.0% solution (Diquas®)	AT (Soft Santear®; NaCl 0.4% and KCl 0.1%)	-	48.1 ± 13.0	70.2	47/47*	4 weeks	No/No
Gao, 2022_a	RCT	Patients with SS and DED	CsA 0.05% nanoemulsion	AT (NaHA)	• Drug: 2 times/day<br>• AT: 4 times/day	51.4 ± 12.4/ 58.4 ± 9.6	10.0/5.0	20/20	12 weeks	Yes/Yes
Gao, 2022_b	RCT	Patients with SS and DED	CsA 0.05% nanoemulsion + AT (NaHA)	AT (NaHA)	• Drug: 2 times/day<br>• AT: 4 times/day	56.4 ± 12.4/ 58.4 ± 9.6	5.0/5.0	20/20	12 weeks	Yes/Yes
Goldberg, 2019	RCT	Patients with DED	CsA 0.09% nanomicellar solution§	Vehicle§	2 times/day (both)	58.4 ± 14.1/ 59.5± 14.7	15.9	371/373	12 weeks	No/Yes
Goldstein, 2017	RCT	Patients with moderate to severe DED	Isunakinra 5 or 20 mg/ml solution#	Placebo (vehicle)#	3 times/day (both)	64.1 (28.6-90.5)/ 59.4 (23.7-86.3)	20.0/23.0	44/30	6 weeks	No/Yes
Gündüz, 1994	RCT	Patients with SS and KCS	CsA 2.0% in olive oil + AT (carboxypolymethylene gel)	Placebo (vehicle) + AT (carboxypolymethylene gel)	4 times/day (both)	52.1 ± 1.1	10.0	15/15	2 months	No/Yes
Guzey, 2009	RCT	Patients with severe trachomatous dry eye	CsA 0.05% emulsion (Restasis®) + AT (Tears Naturale® Free; HMC 0.3% and dextran 0.1%)	Vehicle + AT (Tears Naturale® Free; HMC 0.3% and dextran 0.1%)	• Drug/vehicle: 2 times/day<br>• AT: 5 times/day	61.5 ± 6.9/ 60.5 ± 8.2	43.8/46.9	32/32	6 months	Yes/No
Holland, 2017	RCT	Patients with DED	Lifitegrast 5.0% solution§	Placebo§	2 times/day (both)	58.8 ± 14.1/ 58.6 ± 14.8	24.5/24.4	355/356	12 weeks	No/Yes
Hom, 2006	RCT	Patients with contact lens-related dry eye	CsA 0.05% emulsion (Restasis®)	AT (Refresh Contacts®; CMC 0.5%)	2 times/day (both)	-/-	-/-	17 (in total)	5 weeks	No/No
Igarashi, 2015	RCT	Patients with dry eye after corneal surgery	Rebamipide suspension	AT	4 times/day (both)	34.4 ± 10.8/ 33.7 ± 10.8	13.3/10.0	15/10	4 weeks	Yes/No
Jeon, 2021	RCT	Patients with DED in contact lens wearers	AT (CMC)	No treatment	As often as needed	21.0 ± 2.2/ 21.5 ± 1.6	15.4/7.7	26/26	4 weeks	Yes/No
Jo, 2022_a	RCT	Patients with DED and MGD	CsA 0.05% emulsion (Restasis®) + AT (Hyal Q®; NaHA 0.1%)	AT (Hyal Q®; NaHA 0.1%)	• Drug: 2 times/day<br>• AT: 4 times/day	58.2 ± 14.4/ 64.1 ± 9.9	29.2/47.4	24/19	12 weeks	No/No
Jo, 2022_b	RCT	Patients with DED and MGD	CsA 0.05% nanoemulsion (Cyporin N®) + AT (Hyal Q®; NaHA 0.1%)	AT (Hyal Q®; NaHA 0.1%)	• Drug: 2 times/day<br>• AT: 4 times/day	57.2 ± 15.7/ 64.1 ± 9.9	9.5/47.4	21/19	12 weeks	No/No
Johnson, 2006	Randomized cross-over study	Patients with moderate DED	AT (NaHA 0.1% or 0.3%)	Saline solution (Minims®; NaCl 0.9%)	-	21-34	38.5	13/13	6 hours	No/No
Kaido, 2018	RCT	Patients with short TBUT dry eye	Diquafosol 3.0% solution (Diquas®)	AT (Soft Santear®; NaCl 0.4% and KCl 0.1%)	6 times/day (both)	42.6 ± 9.3/ 36.4 ± 6.7	29.6	12/15	5 weeks	No/No
Kheirkhah, 2015	RCT	Patients with DED and MGD	Loteprednol etabonate 0.5% suspension (Lotemax®)	AT (Advanced Eye Relief Dry Eye®; glycerin 0.2% and propylene glycol 0.6%)	2 times/day (both)	52.0 ± 12.0/ 57.0 ± 12.0	47.1/25.0	17/20	4 weeks	Yes/No
Kim, 2017	RCT	Patients with short TBUT dry eye	Diquafosol 3.0% solution (Diquas®) + AT (Hyaluni® – NaHA 0.15%; Liposic EDO Gel® – Carbomer 2 mg/ml)	AT (Hyaluni® – NaHA 0.15%; Liposic EDO Gel® – Carbomer 2 mg/ml)	4 times/day (both)	50.7 ± 11.1/ 51.2 ± 11.8	18.0/23.9	50/46	3 months	No/No
Kim, 2018	RCT	Patients with DED and MGD	CsA 0.05% emulsion (Restasis®) + AT (NaHA 0.1%)	AT (NaHA 0.1%)	• Drug: 2 times/day<br>• AT: 4 times/day	60.4 ± 10.8/ 57.9 ± 9.5	27.0/43.8	74/32	3 months	Yes/No
Laibovitz, 1993	Randomized cross-over study	Patients with KCS	CsA 1.0% ointment + AT (Refresh®; CMC 0.5%)	Placebo (vehicle) + AT (Refresh®; CMC 0.5%)	• Drug/placebo: 2 times/day<br>• AT: 4 times/day	60 (24-79)	7.7	25 (in total)	6 weeks	No/Yes

Lee, 2006	RCT	Patients with KCS	Prednisolone 0.1%	AT (HA 0.1%)	3 times/day (both)	49.9 ± 13.0	26.8	41/41*	28 days	Yes/No
Lee, 2014_a	NRCT	Patients with level 1 and 2 DED	Fluorometholone 0.1% (Ocumetholone®) + CsA 0.05% emulsion (Restasis®) + isotonic AT (Kynex®; 0.1% NaHA)	Isotonic AT (Kynex®; 0.1% NaHA)	• Drugs: 2 times/day<br>• AT: 4 times/day	51.5 ± 14.2/ 56.9 ± 8.6	46.7	15/15	3 months	No/No
Lee, 2014_b	NRCT	Patients with level 1 and 2 DED	Fluorometholone 0.1% (Ocumetholone®) + CsA 0.05% emulsion (Restasis®) + hypotonic AT (Kynex 2®; 0.18% NaHA)	Hypotonic AT (Kynex 2®; 0.18% NaHA)	• Drugs: 2 times/day<br>• AT: 4 times/day	52.1 ± 16.8/ 54.1 ± 9.9	43.3	15/15	3 months	No/No
Lee, 2022_a	RCT	Patients with moderate to severe DED	CsA 0.05% emulsion (Restasis®) + AT (New Hyaluni®; NaHA 0.15%)	AT (New Hyaluni®; NaHA 0.15%)	• Drug: 2 times/day<br>• AT: 6 times/day	-/-	-/-	121/122	12 weeks	Yes/Yes
Lee, 2022_b	RCT	Patients with moderate to severe DED	CsA 0.05% emulsion (Restasis®) + AT (Refresh plus®; CMC 0.5%)	AT (New Hyaluni®; NaHA 0.15%)	• Drug: 2 times/day<br>• AT: 6 times/day	-/-	-/-	121/122	12 weeks	Yes/Yes
Lekhanont, 2014	RCT	Patients with moderate and severe DED	Hypotonic AT (Vislube/Vismed®; NaHA 0.18%)	Saline solution (NaCl 0.9%)	-	49.1 ± 14.7	12.0	50/50*	120 min after instillation	No/No
Leonardi, 2016	RCT	Patients with severe DED	CsA 0.1% emulsion (Ikervis®)[§]	Vehicle[§]	Once daily (both)	60.8 ± 13.5/ 62.1 ± 11.8	18.2/8.8	254/91	6 months	Yes/Yes
Liew, 2012_a	RCT	Patients with DED	CsA 0.05% emulsion[#]	Vehicle[#]	2 times/day	58.9 ± 14.3/ 62.3 ± 12.5	19.1/31.9	47/47	8 weeks	Yes/Yes
Liew, 2012_b	RCT	Patients with DED	Tofacitinib 0.0003%, 0.001%, 0.003% or 0.005% solution[#]	Vehicle[#]	• Drug/vehicle: 2 times/day<br>• Drug 0.005%: once daily	59.7 ± 12.9/ 62.3 ± 12.5	20.6/31.9	233/47	8 weeks	Yes/Yes
Liu, 2012	RCT	Patients with DED	Pranoprofen 0.1% solution (Pranopulin®) + AT (Hialid®; NaHA 0.1%)	AT (Hialid®; NaHA 0.1%)	4 times/day (both)	41.5 ± 6.2 42.1 ± 7.9	40.0/33.3	30/30	30 days	Yes/Yes
Liu, 2023	RCT	Patients with mild to moderate DED	Diquafosol 3.0% solution (Diquas®) + AT (Hialid®; NaHA 0.3%)	AT (Hialid®; NaHA 0.3%)	• Drug: 6 times/day<br>• AT: 4 times/day	31.9 ± 6.7/ 31.4 ± 5.7	15.0/12.2	40/41	3 months	Yes/No
López-de la Rosa, 2017	Randomized cross-over study	Patients with moderate to severe dry eye	AT (Visaid®; NaHA 0.3%)	Saline solution (NaCl 0.9%)	3-8 times/day (both)	57.5 ± 12.8	25.0	8/8	1 month	Yes/Yes
Mah, 2008	RCT	Patients with allergic conjunctivitis and DED	Olopatadine hydrochloride 0.2% solution (Pataday®)^	Saline solution (Sensitive Eyes®; NaCl 0.9%)^	Once daily (both)	55.5	42.3	25/27	1 week	No/No
McDonald, 2014	RCT	Daily disposable soft contact lens wearers who experienced eye dryness	AT (Systane®; polyethylene glycol 400 0.4% and propylene glycol 0.3%)	No treatment	2 times/day	31.3 ± 10.6/ 30.7 ± 8.6	43.0/24.0	45/43	2 weeks	No/No
Miyake, 2016	RCT	Patients with DED	Diquafosol 3.0% solution (Diquas®)	AT (Soft Santear®; NaCl 0.4% and KCl 0.1%)	-	58.7 ± 17.4	14.0	25/25*	3h 40 min	No/No
Miyake, 2017	RCT (part II)	Patients with definite DED or probable DED after cataract surgery	Diquafosol 3.0% solution (Diquas®)	AT (Mytear®; CMC 0.5%)	6 times/day (both)	71.7 ± 7.4/ 70.3 ± 7.1	40.0/29.1	75/79	4 weeks	Yes/No
Moawad, 2021_a	RCT	Patients with SS and severe DED	Tacrolimus 0.03% nanoemulsion[#]	Saline solution[#]	2 times/day (both)	44.9 ± 12.6	10.0	30/30*	6 months	Yes/No
Moawad, 2021_b	RCT	Patients with SS and severe DED	CsA 0.05% emulsion[#]	Saline solution[#]	2 times/day (both)	49.4 ± 12.9	10.0	30/30*	6 months	Yes/No
Moon, 2007	NRCT	Patients with moderate to severe DED	CsA 0.05% microemulsion + AT (chondroitin sulfate 0.08% and NaHA 0.06%)	AT (chondroitin sulfate 0.08% and NaHA 0.06%)	4 times/day (both)	49.3 ± 11.4	19.1	36/36*	6-8 weeks	Yes/No
Moon, 2024	RCT	Patients with mild to moderate DED	CsA 0.05% nanoemulsion (Cyporin N®)	AT (New Hyaluni®; NaHA 0.15%)	2 times/day (both)	41.1 ± 11.4/ 47.9 ± 16.9	13.3/13.3	15/15	12 weeks	Yes/No

Ogami, 2021	NRCT	Patients with soft contact lens-related DED	Diquafosol 3.0% solution	AT (Soft Santear®; NaCl 0.4% and KCl 0.1%)	6 times/day (both)	37.6 ± 9.6	7.7	26/26*	8 weeks	Yes/Yes
Ogawa, 2010	NRCT	Patients with chronic GVHD and mild dry eye	Tranilast + AT (composition unknown)	AT (composition unknown), NaHA and vitamin A	4 times/day (both)	47.0/37.0	62.5/30.0	8/10	3 months	No/No
Park, 2014	RCT	Patients with DED	CsA 0.05% emulsion (Restasis®)	AT (Hyalein® – NaHA 0.1%; New Hyaluni® – NaHA 0.15%; Hyaluni® – NaHA 0.3%)	• Drug: 2 times/day<br>• AT: 5-6 times/day	45.2 ± 15.4/ 45.0 ± 14.6	24.4/13.7	45/131	12 weeks	Yes/No
Peng, 2022	RCT	Patients with moderate to severe DED	CsA 0.05% gel (CyclAGel®) + AT (hypromellose methylcellulose)	Vehicle + AT (hypromellose methylcellulose)	• Drug: once daily<br>• AT: 3 times/day	45.0 ± 13.7/ 44.5 ± 14.2	18.1/16.3	315/312	84 days	Yes/Yes
Peng, 2024	RCT	Patients with moderate to severe DED	CsA 0.1% solution§	Vehicle§	2 times/day (both)	46.7 ± 14.1/ 48.9 ± 14.4	13.6/6.8	103/103	29 days	No/Yes
Plugfelder, 2004	RCT	Patients with KCS and delayed tear clearance	Loteprednol etabonate 0.5% suspension (Lotemax®)#	Placebo (vehicle)#	4 times/day (both)	57.6/56.2	37.5/11.8	32/34	4 weeks (and 2 weeks after discontinuation)	Yes/Yes
Pinto-Fraga, 2016	RCT	Patients with moderate to severe DED	Fluorometholone 0.1% suspension (FML®)	Vehicle	4 times/day (both)	59.0 ± 8.8/ 60.3 ± 9.7	-/-	21/19	21 days	Yes/Yes
Pinto-Fraga, 2017	Randomized cross-over study	Patients with mild DED	AT (Visaid®; HA 0.2%)	Saline solution (NaCl 0.9%)	3-8 times/day (both)	58.0 ± 20.0	50.0	8/8	1 month	Yes/Yes
Prabhasawat, 2012	RCT	Patients with MGD and unstable tear film	CsA 0.05% emulsion (Restasis®)	AT (Cellufresh®; CMC 0.5%)	2 times/day (both)	48.1 ± 13.9/ 55.0 ± 13.0	19.4/17.6	21/33	3 months	Yes/No
Rao, 2010	RCT	Patients with DED	CsA 0.05% emulsion (Restasis®)	AT (Refresh Endura®; glycerin 1.0% and polysorbate 80 1.0%)	2 times/day (both)	47.5 ± 5.9/ 48.2 ± 6.3	31.0/27.0	36/22	12 months	Yes/No
Rao, 2011	RCT	Patients with DED	CsA 0.05% emulsion (Restasis®)	AT (Refresh Endura®; glycerin 1.0% and polysorbate 80 1.0%)	2 times/day (both)	-/-	-/-	40/8	12 months	No/No
Rhim, 2022	RCT	Patients with DED and obstructive MGD	CsA 0.05% nanoemulsion (Cyporin N®) + AT (New Hyaluni®; NaHA 0.15%)	AT (New Hyaluni®; NaHA 0.15%)	• Drug: 2 times/day<br>• AT: 4-6 times/day	36.9 ± 7.9/ 40.1 ± 8.7	3.8/16.0	26/25	3 months	Yes/No
Rolando, 2023	RCT	Patients with DED	Hydrocortisone 0.335 mg/ml solution (Idroflog®)	AT (Zerodue®; HA 0.2%)	4 times/day (both)	62.0 ± 14.0	12.5	20/20	6 months	No/Yes
Salib, 2006	RCT	Patients with DED undergoing laser in situ keratomileusis	CsA 0.05% emulsion (Restasis®)	AT (Refresh®; CMC 0.5%)	2 times/day (both)	47 (40-59)	10.0	14-22* (Schirmer's test), 7-11 (OSDI)	12 months	No/No
Sall, 2000	RCT	Patients with moderate to severe DED	CsA 0.05% or 0.1% emulsions#	Vehicle#	2 times/day (both)	59.4 ± 13.6/ 59.9 ± 14.3	17.6/20.2	585/292	6 months	No/Yes
Sall, 2006	RCT	Patients with aqueous deficient dry eye	CsA 0.05% emulsion (Restasis®) + AT (Systane®; polyethylene glycol 400 0.4% and propylene glycol 0.3%)	AT (Systane®; polyethylene glycol 400 0.4% and propylene glycol 0.3%)	• Drug: 2 times/day<br>• AT: 1-4 times/day	59.5	19.7	20/20	6 months	No/No
Schmidl, 2015_a	RCT	Patients with moderate DED	AT (Thealoz Duo®; NaHA 1.5 mg/ml + trehalose 30 mg/ml)	Saline solution (Hydrabak®; NaCl 0.9%)	-	42.7 ± 11.6	28.3	60 (in total)	240 min	No/No
Schmidl, 2015_b	RCT	Patients with moderate DED	AT (Hyabak®; NaHA 0.15%)	Saline solution (Hydrabak®; NaCl 0.9%)	-	42.7 ± 11.6	28.3	60 (in total)	240 min	No/No
Schmidl, 2017	RCT (cohort I)	Patients with DED	C-NAC 0.1% w/v^	Saline solution (NaCl 0.9%)^	Once daily (both)	-/-	-/-	21/21*	2 days	No/No
Semba, 2012	RCT	Patients with DED	Lifitegrast 0.1%, 1.0% or 5.0% solutions§	Placebo (vehicle)§	2 times/day (both)	63.0 ± 12.4/ 60.4 ± 12.9	22.1/22.4	172/58	12 weeks	No/Yes
Sharma, 2025	RCT	Patients with DED	Lifitegrast 5.0% solution	AT (CMC 0.5%)	2 times/day (both)	40.8 ± 12.7/ 42.0 ± 11.8	46.0/55.7	176/175	12 weeks	No/No

Sheppard, 2014	RCT	Patients with DED	Lifitegrast 5.0% solution§	Placebo (vehicle)§	2 times/day (both)	60.2 ± 12.2/ 61.1 ± 11.8	21.8/26.4	293/295	12 weeks	No/Yes
Sheppard, 2021	RCT	Patients with DED	CsA 0.1% solution (CyclASol®)§	Vehicle§	2 times/day (both)	61.5 ± 13.6/ 61.3 ± 12.7	28.4/28.3	162/166	12 weeks	No/Yes
Shettle, 2022	RCT	Patients with severe DED	Licaminlimab 60 mg/ml solution#	Vehicle#	3 times/day (both)	61.7 ± 13.1/ 58.8 ± 14.5	11.6/16.9	69/65	6 weeks	No/Yes
Shimazaki-Den, 2013	RCT (part II)	Patients with DED and unstable tear film	Diquafosol 3.0% solution (Diquas®)	AT (Soft Santear®; NaCl 0.4% and KCl 0.1%)	6 times/day (both)	44.2 ± 14.8	11.8	9/8	4 weeks	No/No
Sindhu, 2015	NRCT	Patients with DED	Fluorometholone 0.1% or loteprednol etabonate 0.5% + AT (CMC 0.5%)	AT (CMC 0.5%)	3 times/day (both)	46.0 ± 1.8	43.3	30/30	12 weeks	Yes/Yes
Stevenson, 2000	RCT	Patients with moderate to severe DED	CsA 0.05%, 0.1%, 0.2% or 0.4% emulsions#	Vehicle#	2 times/day (both)	59 (31-88)	16.0	74/16	12 weeks	No/Yes
Sullivan, 1997	Randomized cross-over study	Patients with moderate to severe DED	AT (Carbopol 934P®; carbomer 0.3% gel)	Placebo	-	59.7 ± 10.9/ 61.0 ± 14.4	24.2/8.2	62/61	8 weeks	No/No
Tan, 2024_a	RCT	Patients with moderate to severe DED	AT (Hylo-Forte®; NaHA 0.2%)	Saline solution (NaCl 0.9%)	4 times/day (both)	36.9 ± 8.7/ 42.6 ± 19.2	12.5/31.3	16/16	4 weeks	Yes/Yes
Tan, 2024_b	RCT	Patients with moderate to severe DED	AT (Systane Hydration®; NaHA 0.15% and hydroxypropyl guar 0.175%)	Saline solution (NaCl 0.9%)	4 times/day (both)	36.9 ± 11.0/ 42.6 ± 19.2	62.5/31.3	16/16	4 weeks	Yes/Yes
Tauber, 1998	RCT	Patients with moderate to severe KCS	CsA 0.05%, 0.1%, 0.2% or 0.4% emulsions#	Vehicle#	2 times/day (both)	-/-	-/-	133 (in total)	12 weeks	No/No
Tauber, 2004	RCT	Patients with DED	Diquafosol 1.0% or 2.0% solutions#	Placebo (vehicle)#	4 times/day (both)	61.1 ± 12.7 / 60.9 ± 13.0	18.2/20.0	351/176	24 weeks	No/Yes
Tauber, 2015	RCT	Patients with DED	Lifitegrast 5.0% solution^	Placebo^	2 times/day (both)	58.7 ± 13.9/ 58.9 ± 14.3	20.4/26.4	358/360	12 weeks	No/Yes
Tauber, 2018	RCT	Patients with DED	CsA 0.09% or 0.05% nanomicellar solution§	Placebo (vehicle)§	2 times/day (both)	60.7 ± 14.0/ 59.3 ± 13.8	20.5/21.1	303/152	12 weeks	No/Yes
Taylor, 2019	RCT	Patients with DED	TOP1630 0.1% solution§	Placebo (vehicle)§	3 times/day (both)	62.5 ± 11.4/ 65.3 ± 11.9	35.5/46.7	31/30	28 days	No/Yes
Teshigawara, 2021	RCT	Patients with DED	Rebamipide 2.0% suspension (Mucosta®)	AT (Mytear®; CMC 0.5%)	4 times/day (both)	66.6 ± 12.4/ 66.1 ± 12.4	23.3/28.6	30/28*	4 weeks	Yes/Yes
Tityal, 2019	RCT	Patients with mild to moderate DED	Cloroquine 0.03% + AT (CMC 0.5%)	AT (CMC 0.5%)	• Drug: 2 times/day<br>• AT: 3 times/day	58.5 ± 13.9/ 56.9 ± 13.5	60.0/66.7	75/75	3 months	Yes/Yes
Toda, 2014_a	RCT	Patients with DED after laser in situ keratomileusis	Diquafosol 3.0% solution (Diquas®) + AT (Hyalein®; NaHA 0.3%)	AT (Hyalein®; NaHA 0.3%)	6 times/day (both)	30.4 ± 9.2/ 31.4 ± 10.6	40.7/43.5	27/23	1 month	No/No
Toda, 2014_b	RCT	Patients with DED after laser in situ keratomileusis	Diquafosol 3.0% solution (Diquas®)	AT (Soft Santear®; NaCl 0.4% and KCl 0.1%)	6 times/day (both)	34.5 ± 11.2/ 37.0 ± 8.4	26.7/44.0	30/25	1 month	No/No
Toda, 2014_c	RCT	Patients with DED after laser in situ keratomileusis	Diquafosol 3.0% solution (Diquas®)	AT (Hyalein®; NaHA 0.3%)	6 times/day (both)	34.5 ± 11.2/ 31.4 ± 10.6	26.7/43.5	30/23	1 month	No/No
Uchiyama, 2008	Randomized cross-over study	Patients with KCS	AT (Systane®; polyethylene glycol 400 0.4% and propylene glycol 0.3%)	Saline solution (NaCl 0.9%)	-	51-83	25.0	12/12	60 min	No/No
Utsunomiya, 2017	NRCT	Patients with DED	Diquafosol 3.0% solution (Diquas®)	AT (Soft Santear®; NaCl 0.4% and KCl 0.1%)	6 times/day (both)	58.8 ± 14.6/ 52.1 ± 15.4	38.5/12.5	39/24	2 weeks	No/No
Wang, 2008	NRCT	Patients with DED and chronic GVHD	CsA 0.05% solution + AT (composition unknown)	AT (composition unknown)	• Drug: 4 times/day<br>• AT: 6 times/day	49.7 ± 15.6/ 39.5 ± 10.1	46.2	7/6	1 month	No/No
Willen, 2008	RCT	Patients with DED wearing contact lens	CsA 0.05% emulsion (Restasis®)	Placebo	2 times/day (both)	44.0 ± 12.6/ 42.2 ± 14.8	18.2/13.6	22/22	3 months	No/No
Wirta, 2019	RCT	Patients with moderate to severe DED not responding to AT	CsA 0.05% or 0.1% solution (CyclASol®) or CsA 0.05% emulsion (Restasis®)	Vehicle	2 times/day (both)	62.7 ± 11.7/ 61.3 ± 10.5	26.5/25.0	155/52	16 weeks	No/Yes

Wong, 2024	RCT	Patients with DED and chronic GVHD	Lifitegrast 5.0% solution§	Placebo (vehicle)§	-	59.0 ± 12.8	-/-	10/6	4 weeks	Yes/No
Xu, 2023_a	RCT	Patients with moderate to severe DED	CsA 0.05% emulsion (Restasis®)	AT (New Hyaluni®; NaHA 0.15%)	• Drug: 2 times/day<br>• AT: 2-3 times/day	20.0 ± 2.0/ 20.2 ± 1.3	22.3/23.4	94/94	12 weeks	Yes/Yes
Xu, 2023_b	RCT	Patients with moderate to severe DED	Diquafosol 3.0% solution (Diquas®)	AT (New Hyaluni®; NaHA 0.15%)	• Drug: 6 times/day<br>• AT: 2-3times/day	20.5 ± 1.4/ 20.2 ± 1.3	21.3/23.4	94/94	12 weeks	Yes/Yes

Table S1. Characteristics of the studies included in the systematic review. List of abbreviations: allo-HSCT, allogeneic hematopoietic stem cell transplantation; AT, artificial tears; CMC, carboxymethylcellulose; C-NAC, chitosan–N-acetylcysteine; CsA, cyclosporine; DED, dry eye disease; GVHD, graft-versus-host disease; HA, hyaluronic acid; HMC, hydroxy methylcellulose; KCS, keratoconjunctivitis sicca; MGD, meibomian gland dysfunction; NaHA, sodium hyaluronate; NRCT, nonrandomized controlled trial; OSDI, Ocular Surface Disease Index; PVP, polyvinylpyrrolidone; RCT, randomized controlled trial; SD, standard deviation; SS, Sjögren's syndrome. Symbol legend: * randomization and allocation were performed at the eye level, not the patient level; # use of AT was allowed, if needed; § use of AT was not allowed; ^ use of AT was not specified.

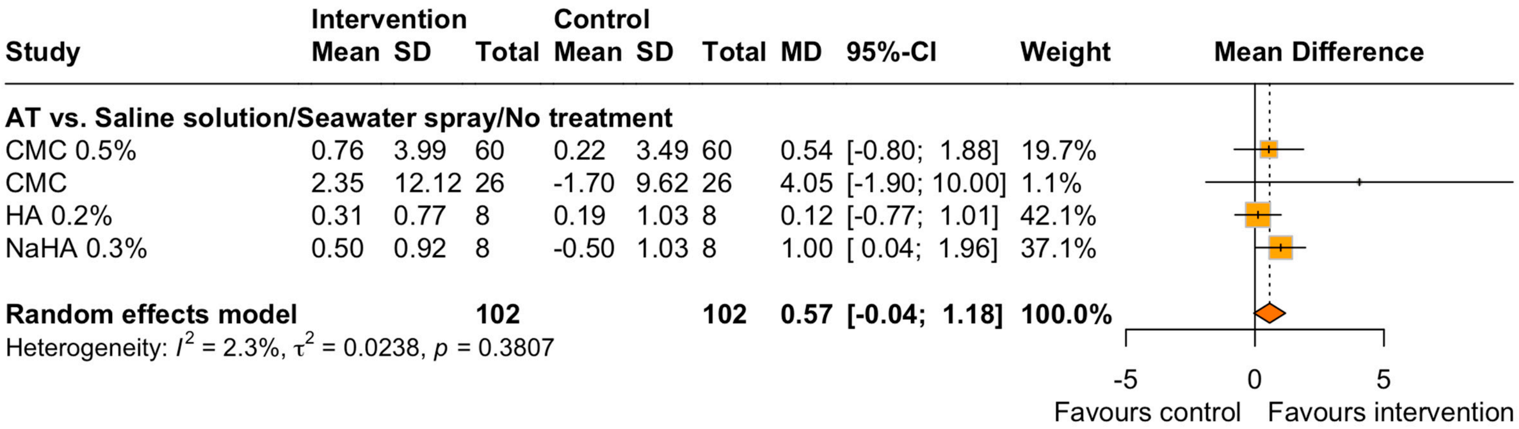

Figure S1. Forest plot of the mean difference (MD) with 95% Confidence Interval (CI) of Schirmer's test results (mm) for artificial tears (AT) vs. saline solution, seawater spray, or no treatment. Other abbreviations: CMC, carboxymethylcellulose; HA, hyaluronic acid; NaHA, sodium hyaluronate.

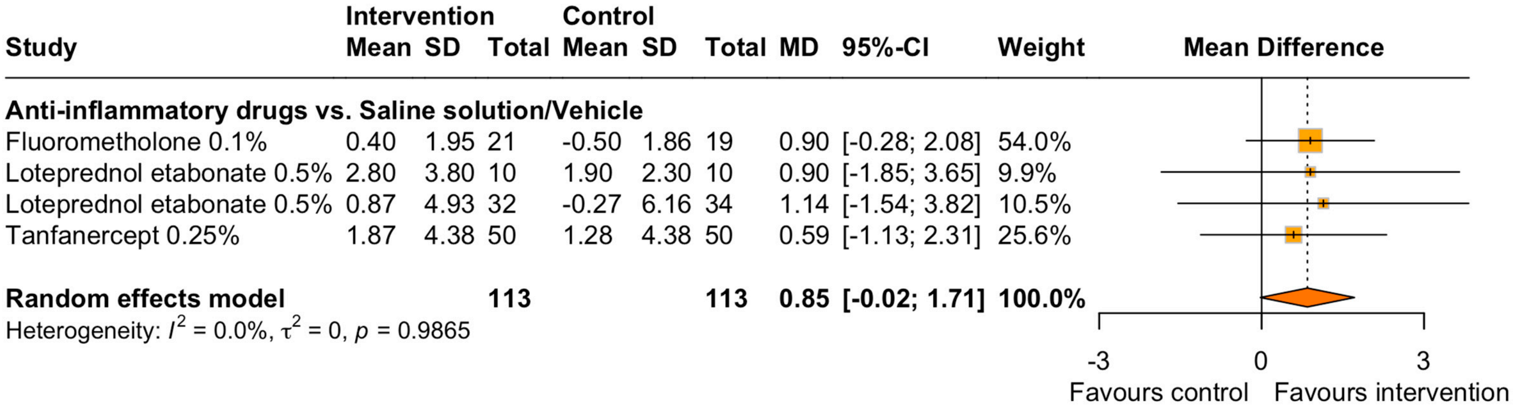

Figure S2. Forest plot of the mean difference (MD) with 95% Confidence Interval (CI) of Schirmer's test results (mm) for anti-inflammatory drugs vs. saline solution or vehicle.

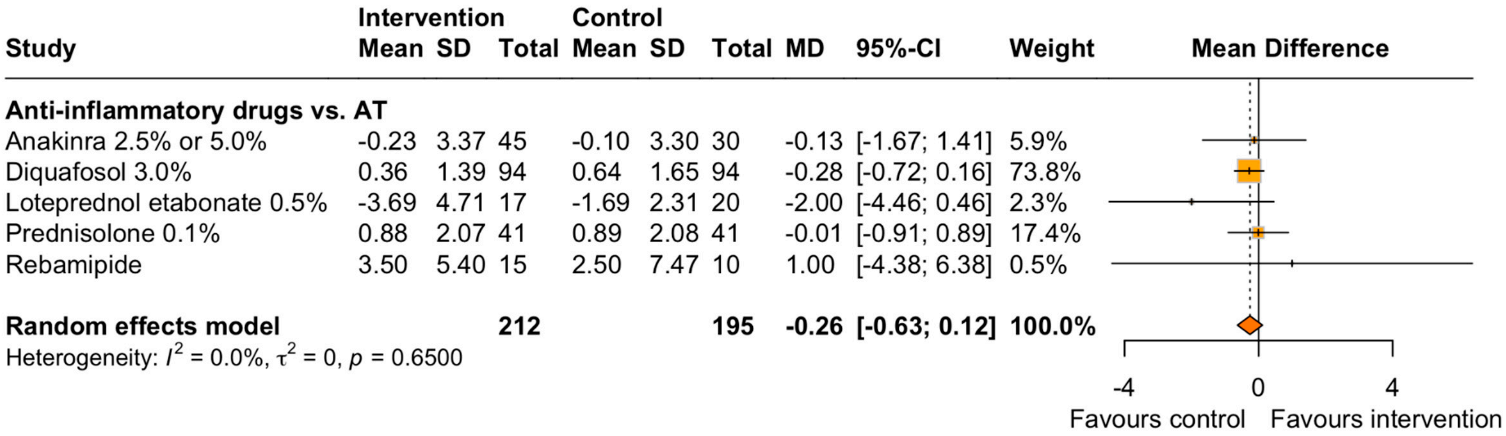

Figure S3. Forest plot of the mean difference (MD) with 95% Confidence Interval (CI) of Schirmer's test results (mm) for anti-inflammatory drugs vs. artificial tears (AT).

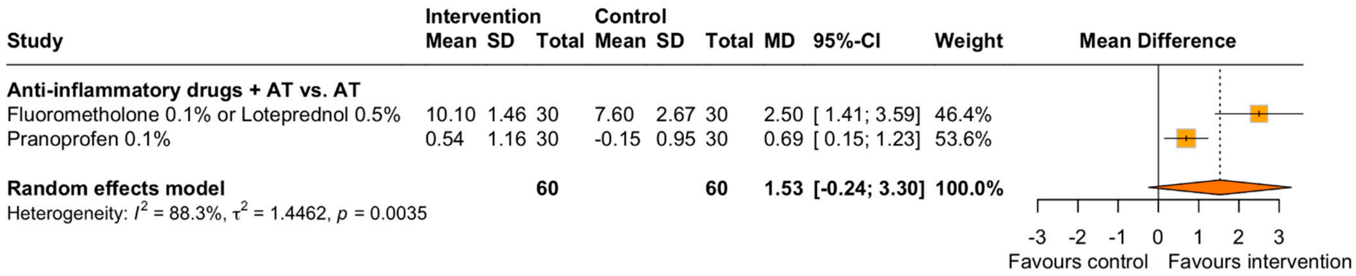

Figure S4. Forest plot of the mean difference (MD) with 95% Confidence Interval (CI) of Schirmer's test results (mm) for the combination of anti-inflammatory drugs and artificial tears (AT) vs. AT in the sensitivity analysis excluding the study on diquafosol.

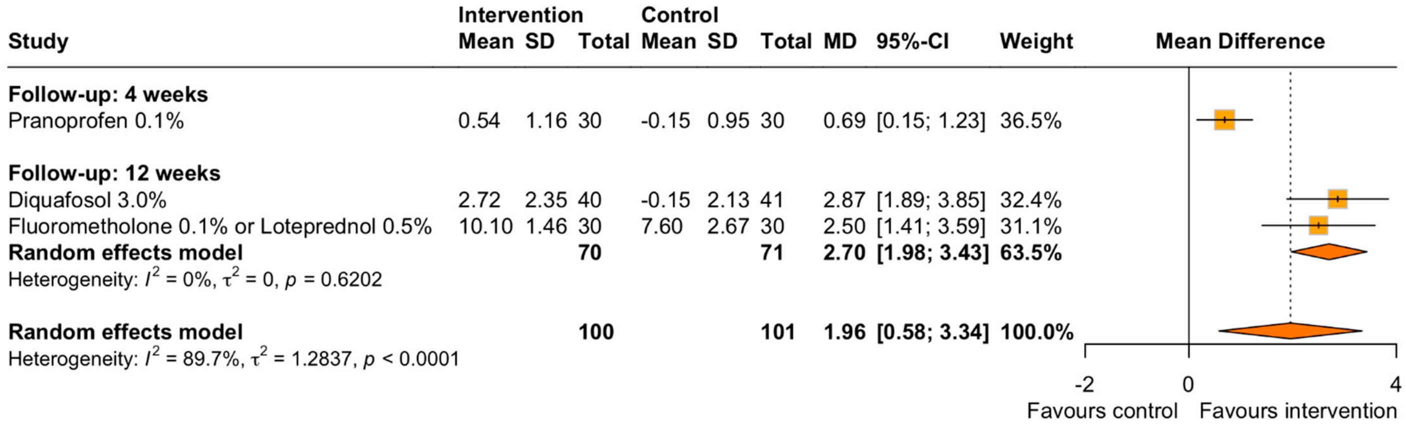

Figure S5. Forest plot of the mean difference (MD) with 95% Confidence Interval (CI) of Schirmer's test results (mm) for the combination of anti-inflammatory drugs with artificial tears (AT) vs. AT, stratified by follow-up.

a)

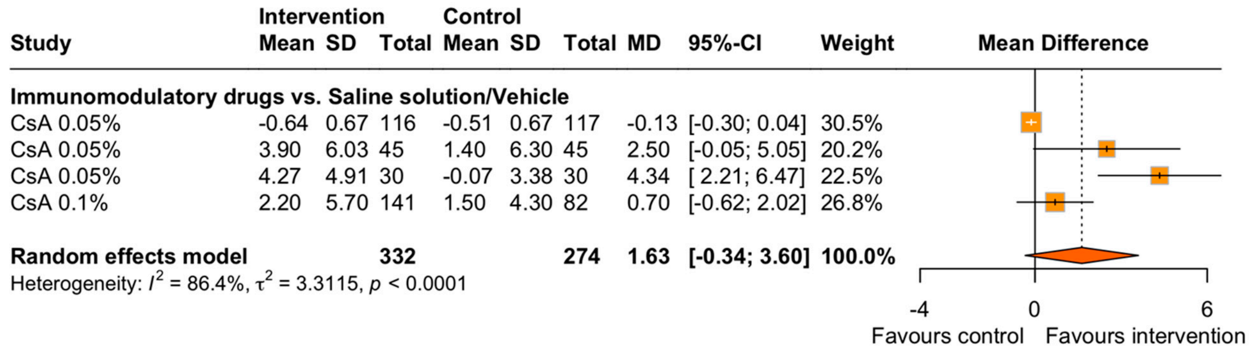

b)

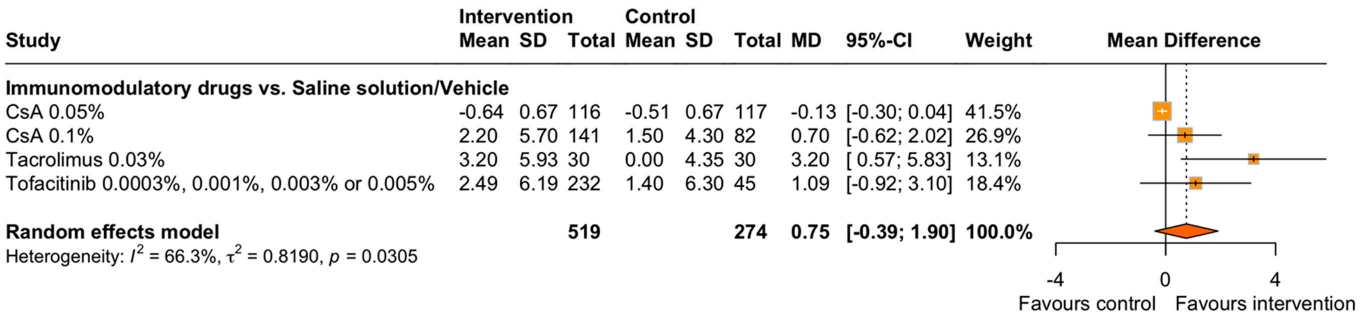

Figure S6. Forest plot of the mean difference (MD) with 95% Confidence Interval (CI) of Schirmer's test results (mm) for immunomodulatory drugs vs. saline solution or vehicle. a) Analysis including one of the two intervention arms that shared the same control group; b) analysis including the other intervention arm. Other abbreviations: CsA, cyclosporine.

Study	Intervention Mean	Intervention SD	Total	Control Mean	Control SD	Total	MD	95%-CI	Weight
**Immunomodulatory drugs vs. AT**									
CsA 0.05%	3.80	2.77	20	-1.15	2.21	20	4.95	[3.40; 6.50]	22.8%
CsA 0.05%	3.13	14.25	15	0.80	7.63	15	2.33	[-5.85; 10.51]	5.7%
CsA 0.05%	1.07	6.57	45	1.48	6.42	131	-0.41	[-2.62; 1.80]	20.5%
CsA 0.05%	1.90	0.72	36	-0.10	0.72	22	2.00	[1.62; 2.38]	25.5%
CsA 0.05%	0.37	1.14	94	0.64	1.65	94	-0.27	[-0.68; 0.14]	25.5%
**Random effects model**			**210**			**282**	**1.62**	**[-0.60; 3.84]**	**100.0%**

Heterogeneity: $I^2 = 95.6\%$, $\tau^2 = 5.0032$, $p < 0.0001$

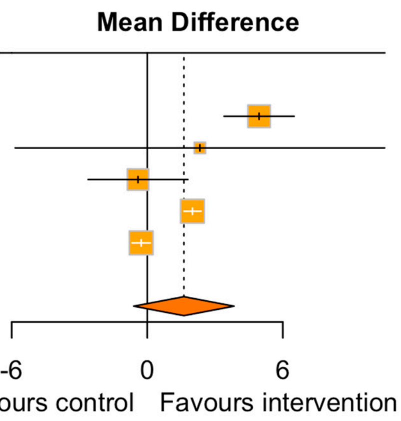

Figure S7. Forest plot of the mean difference (MD) with 95% Confidence Interval (CI) of Schirmer's test results (mm) for immunomodulatory drugs (cyclosporine, CsA) vs. artificial tears (AT). Other abbreviations: CsA, cyclosporine.

Study	Intervention Mean	SD	Total	Control Mean	SD	Total	MD	95%-CI	Weight
**Immunomodulatory drugs + AT vs. AT**									
CsA 0.05%	3.36	2.90	25	7.00	4.37	48	-3.64	[-5.32; -1.96]	10.8%
CsA 0.05%	4.07	3.03	119	1.16	3.03	115	2.91	[2.13; 3.69]	11.4%
CsA 0.05%	5.30	2.13	20	-1.15	2.21	20	6.45	[5.10; 7.80]	11.1%
CsA 0.05%	6.72	2.74	32	0.59	1.10	32	6.13	[5.11; 7.15]	11.3%
CsA 0.05%	1.43	2.08	74	0.81	2.00	32	0.62	[-0.22; 1.46]	11.4%
CsA 0.05%	1.00	0.99	36	0.75	1.08	36	0.25	[-0.23; 0.73]	11.6%
CsA 0.05%	4.10	6.71	315	2.70	5.34	312	1.40	[0.45; 2.35]	11.4%
CsA 0.05%	2.60	4.49	26	2.90	5.24	25	-0.30	[-2.98; 2.38]	9.8%
Chloroquine 0.03%	5.63	3.96	75	1.64	2.82	75	3.99	[2.89; 5.09]	11.3%
**Random effects model**			**722**			**695**	**2.03**	**[-0.07; 4.12]**	**100.0%**

Heterogeneity: $I^2 = 96.5\%$, $\tau^2 = 9.8303$, $p < 0.0001$

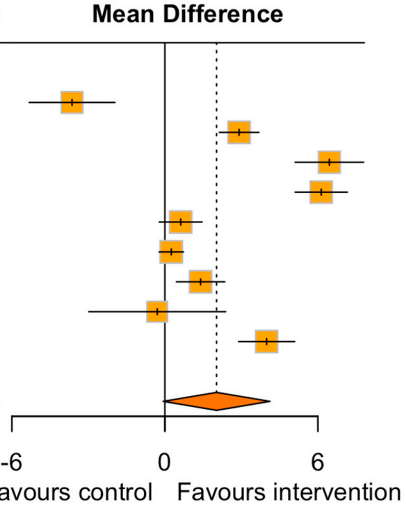

Figure S8. Forest plot of the mean difference (MD) with 95% Confidence Interval (CI) of Schirmer's test results (mm) for the combination of immunomodulatory drugs with artificial tears (AT) vs. AT. Other abbreviations: CsA, cyclosporine.

Study	Intervention Mean	Intervention SD	Intervention Total	Control Mean	Control SD	Control Total	MD	95%-CI	Weight
**Follow-up: 6-8 weeks**									
CsA 0.05%	1.00	0.99	36	0.75	1.08	36	0.25	[-0.23; 0.73]	13.2%
**Follow-up: 12 weeks**									
CsA 0.05%	4.07	3.03	119	1.16	3.03	115	2.91	[2.13; 3.69]	13.0%
CsA 0.05%	5.30	2.13	20	-1.15	2.21	20	6.45	[5.10; 7.80]	12.4%
CsA 0.05%	1.43	2.08	74	0.81	2.00	32	0.62	[-0.22; 1.46]	12.9%
CsA 0.05%	4.10	6.71	315	2.70	5.34	312	1.40	[0.45; 2.35]	12.8%
CsA 0.05%	2.60	4.49	26	2.90	5.24	25	-0.30	[-2.98; 2.38]	10.3%
Chloroquine 0.03%	5.63	3.96	75	1.64	2.82	75	3.99	[2.89; 5.09]	12.7%
**Random effects model**			**629**			**579**	**2.59**	**[0.68; 4.50]**	**74.1%**
Heterogeneity: $I^2 = 92.8\%$, $\tau^2 = 5.2131$, $p < 0.0001$									
**Follow-up: 24 weeks**									
CsA 0.05%	6.72	2.74	32	0.59	1.10	32	6.13	[5.11; 7.15]	12.8%
**Random effects model**			**697**			**647**	**2.73**	**[0.93; 4.52]**	**100.0%**
Heterogeneity: $I^2 = 96.3\%$, $\tau^2 = 6.3030$, $p < 0.0001$									

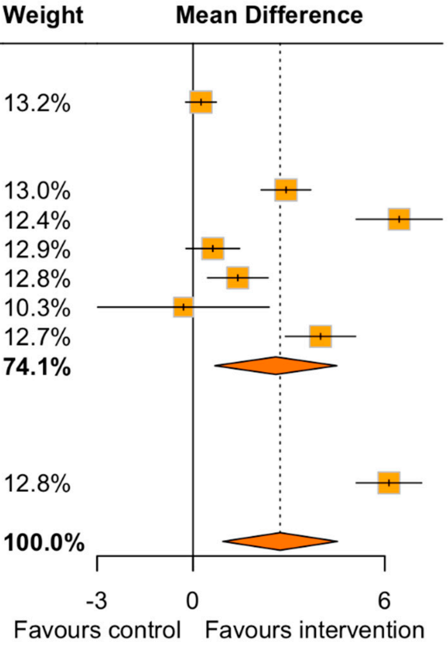

Figure S9. Forest plot of the mean difference (MD) with 95% Confidence Interval (CI) of Schirmer's test results (mm) for the combination of immunomodulatory drugs with artificial tears (AT) vs. AT, stratified by follow-up. Other abbreviations: CsA, cyclosporine.

Study	Intervention Mean	Intervention SD	Intervention Total	Control Mean	Control SD	Control Total	MD	95%-CI	Weight
**Mild to moderate DED**									
Chloroquine 0.03%	5.63	3.96	75	1.64	2.82	75	3.99	[2.89; 5.09]	12.7%
**Moderate to severe DED**									
CsA 0.05%	6.72	2.74	32	0.59	1.10	32	6.13	[5.11; 7.15]	12.8%
CsA 0.05%	4.07	3.03	119	1.16	3.03	115	2.91	[2.13; 3.69]	13.0%
CsA 0.05%	1.00	0.99	36	0.75	1.08	36	0.25	[-0.23; 0.73]	13.2%
CsA 0.05%	4.10	6.71	315	2.70	5.34	312	1.40	[0.45; 2.35]	12.8%
**Random effects model**			**502**			**495**	**2.65**	**[0.16; 5.14]**	**51.8%**
Heterogeneity: $I^2 = 97.4\%$, $\tau^2 = 6.2724$, $p < 0.0001$									
**DED + SS or MGD**									
CsA 0.05%	5.30	2.13	20	-1.15	2.21	20	6.45	[5.10; 7.80]	12.4%
CsA 0.05%	1.43	2.08	74	0.81	2.00	32	0.62	[-0.22; 1.46]	12.9%
CsA 0.05%	2.60	4.49	26	2.90	5.24	25	-0.30	[-2.98; 2.38]	10.3%
**Random effects model**			**120**			**77**	**2.33**	**[-1.83; 6.49]**	**35.6%**
Heterogeneity: $I^2 = 96.4\%$, $\tau^2 = 12.7122$, $p < 0.0001$									
**Random effects model**			**697**			**647**	**2.73**	**[0.93; 4.52]**	**100.0%**
Heterogeneity: $I^2 = 96.3\%$, $\tau^2 = 6.3030$, $p < 0.0001$									

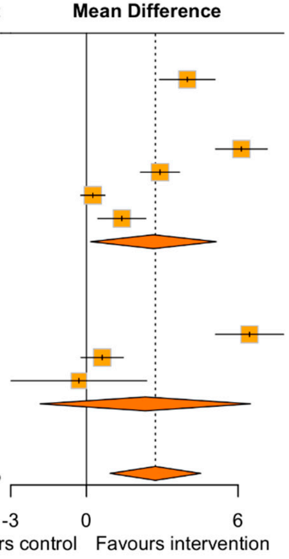

Figure S10. Forest plot of the mean difference (MD) with 95% Confidence Interval (CI) of Schirmer's test results (mm) for the combination of immunomodulatory drugs with artificial tears (AT) vs. AT, stratified by dry eye disease (DED) severity. Other abbreviations: CsA, cyclosporine; MGD, Meibomian gland dysfunction; SS, Sjögren's syndrome.

Study	Intervention Mean	Intervention SD	Intervention Total	Control Mean	Control SD	Control Total	MD	95%-CI	Weight
**AT vs. Saline solution/Seawater spray**									
CMC 0.5%	0.94	1.65	60	0.64	1.57	60	0.30	[-0.28; 0.88]	67.5%
HA 0.2%	0.21	1.09	8	-0.08	1.00	8	0.29	[-0.74; 1.32]	21.3%
NaHA 0.3%	0.38	1.37	8	-0.04	1.51	8	0.42	[-0.99; 1.83]	11.2%
**Random effects model**			**76**			**76**	**0.31**	**[-0.16; 0.78]**	**100.0%**
Heterogeneity: I^2 = 0.0%, τ^2 = 0, p = 0.9871									

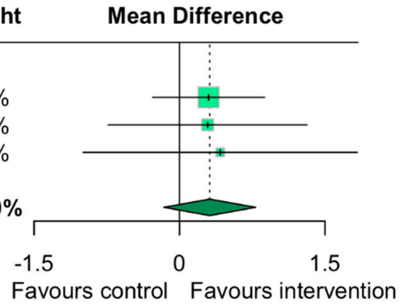

Figure S11. Forest plot of the mean difference (MD) with 95% Confidence Interval (CI) of tear break-up time (TBUT) results (s) for artificial tears (AT) vs. saline solution or seawater spray. Other abbreviations: CMC, carboxymethylcellulose; HA, hyaluronic acid; NaHA, sodium hyaluronate.

Study	Intervention Mean	SD	Total	Control Mean	SD	Total	MD	95%-CI	Weight
**Anti-inflammatory drugs vs. AT**									
Anakinra 2.5% or 5.0%	0.60	1.64	45	0.60	1.40	30	0.00	[-0.69; 0.69]	12.2%
Diquafosol 3.0%	0.60	1.13	75	0.35	1.07	79	0.25	[-0.10; 0.60]	13.7%
Diquafosol 3.0%	0.76	1.39	26	0.37	1.39	26	0.39	[-0.37; 1.15]	11.9%
Diquafosol 3.0%	2.10	2.48	94	1.69	1.95	94	0.41	[-0.23; 1.05]	12.5%
Loteprednol etabonate 0.5%	-0.39	1.51	17	0.55	1.67	20	-0.94	[-1.97; 0.09]	10.3%
Prednisolone 0.1%	2.12	1.06	41	1.65	0.72	41	0.47	[0.08; 0.86]	13.6%
Rebamipide 2.0%	1.67	0.78	30	0.14	0.68	28	1.53	[1.15; 1.91]	13.6%
Rebamipide	2.30	1.21	15	0.10	0.51	10	2.20	[1.51; 2.89]	12.2%
**Random effects model**			**343**			**328**	**0.58**	**[-0.05; 1.20]**	**100.0%**

Heterogeneity: I^2 = 88.3%, τ^2 = 0.7093, p < 0.0001

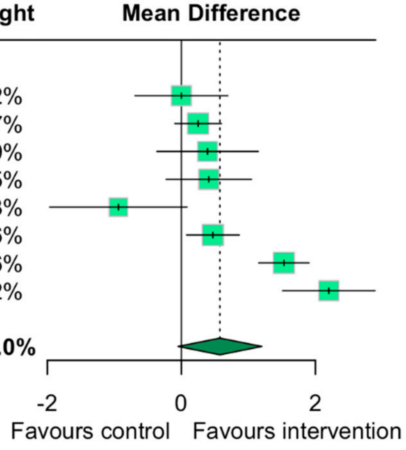

Figure S12. Forest plot of the mean difference (MD) with 95% Confidence Interval (CI) of TBUT results (s) for anti-inflammatory drugs vs. artificial tears (AT).

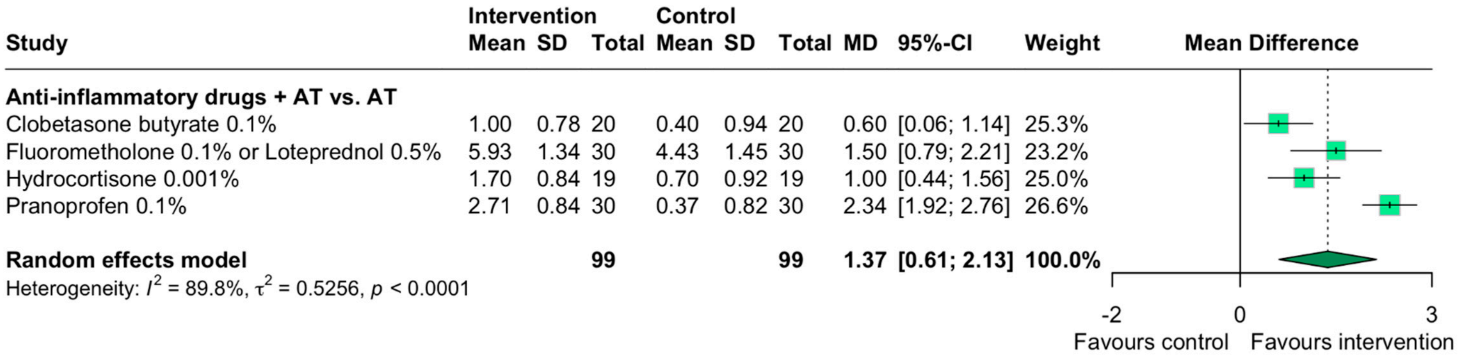

Figure S13. Forest plot of the mean difference (MD) with 95% Confidence Interval (CI) of TBUT results (s) for the combination of anti-inflammatory drugs and artificial tears (AT) vs. AT in the sensitivity analysis excluding the study on diquafosol.

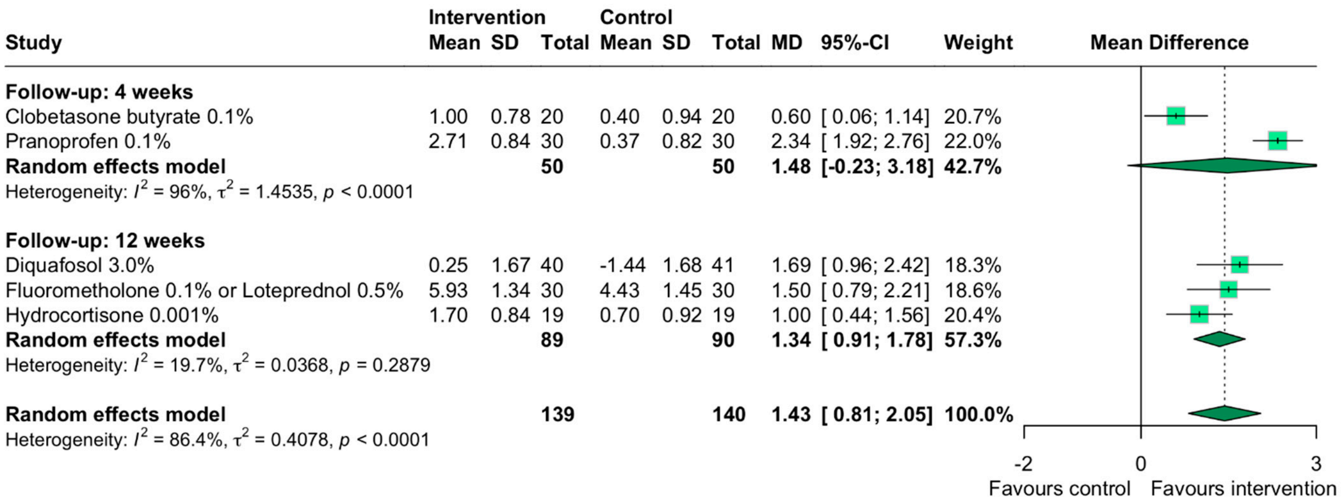

Figure S14. Forest plot of the mean difference (MD) with 95% Confidence Interval (CI) of TBUT results (s) for the combination of anti-inflammatory drugs with artificial tears (AT) vs. AT, stratified by follow-up.

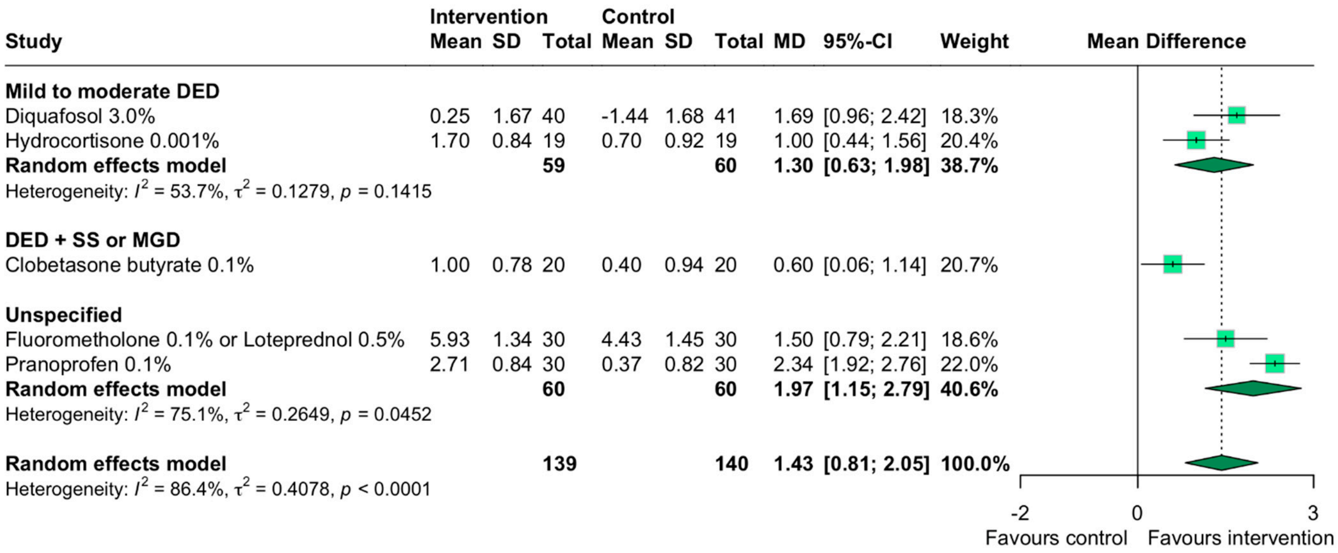

Figure S15. Forest plot of the mean difference (MD) with 95% Confidence Interval (CI) of TBUT results (s) for the combination of anti-inflammatory drugs with artificial tears (AT) vs. AT, stratified by dry eye disease (DED) severity. Other abbreviations: MGD, Meibomian gland dysfunction; SS, Sjögren's syndrome.

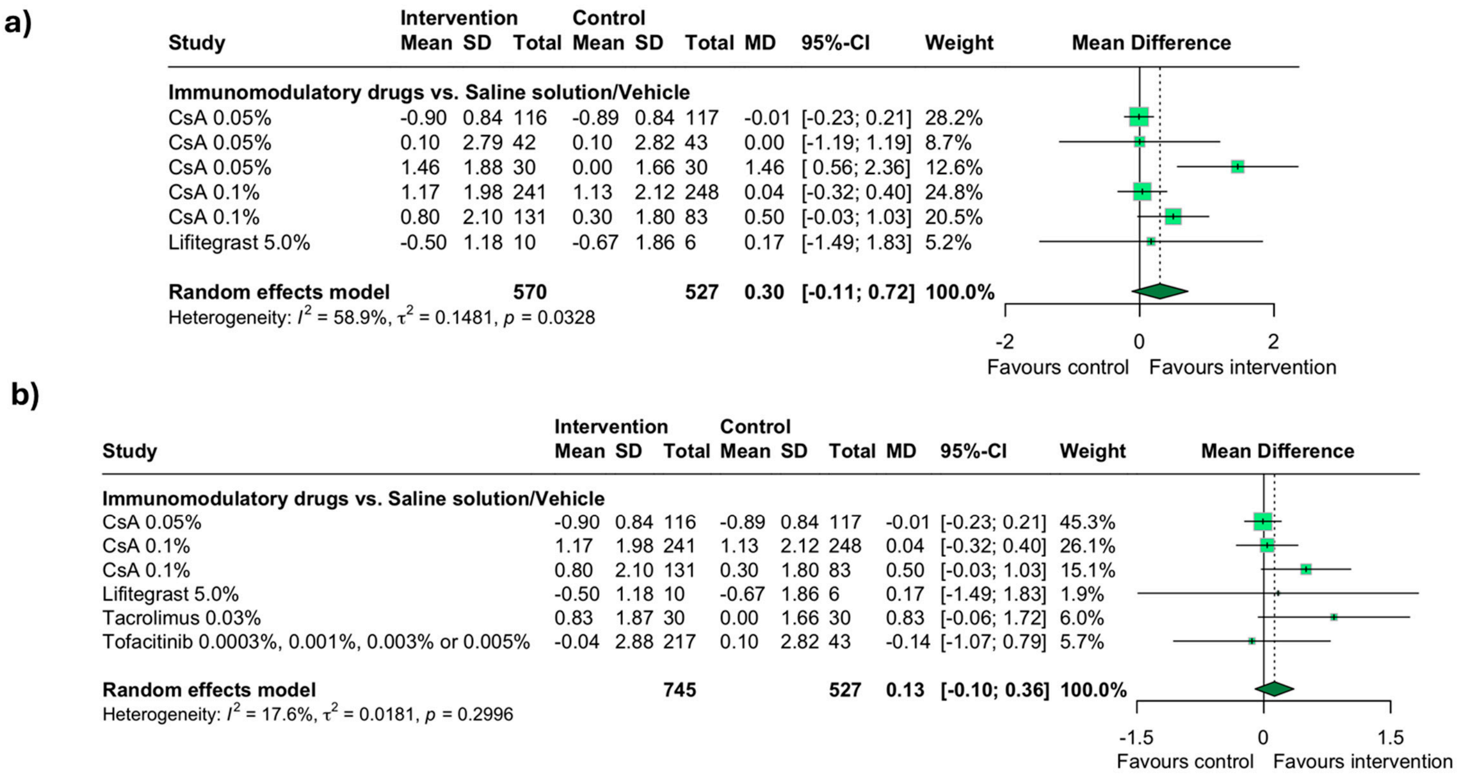

Figure S16. Forest plot of the mean difference (MD) with 95% Confidence Interval (CI) of TBUT results (s) for immunomodulatory drugs vs. saline solution or vehicle. a) Analysis including one of the two intervention arms that shared the same control group; b) analysis including the other intervention arm. Other abbreviations: CsA, cyclosporine.

Study	Intervention Mean	SD	Total	Control Mean	SD	Total	MD	95%-CI	Weight
**Immunomodulatory drugs vs. AT**									
CsA 0.05%	2.25	0.81	20	-0.40	0.73	20	2.65	[2.17; 3.13]	19.4%
CsA 0.05%	1.90	2.45	45	2.24	2.50	131	-0.34	[-1.17; 0.49]	18.0%
CsA 0.05%	1.29	0.18	36	-0.03	0.08	34	1.32	[1.26; 1.38]	20.2%
CsA 0.05%	1.60	0.66	36	-0.40	0.48	22	2.00	[1.71; 2.29]	19.9%
CsA 0.05%	1.64	2.58	94	1.69	1.95	94	-0.05	[-0.70; 0.60]	18.8%
CsA 0.05%	3.20	8.75	15	1.04	4.57	15	2.16	[-2.84; 7.16]	3.7%
**Random effects model**			**246**			**316**	**1.19**	**[0.13; 2.25]**	**100.0%**

Heterogeneity: $I^2 = 93.9\%$, $\tau^2 = 1.4500$, $p < 0.0001$

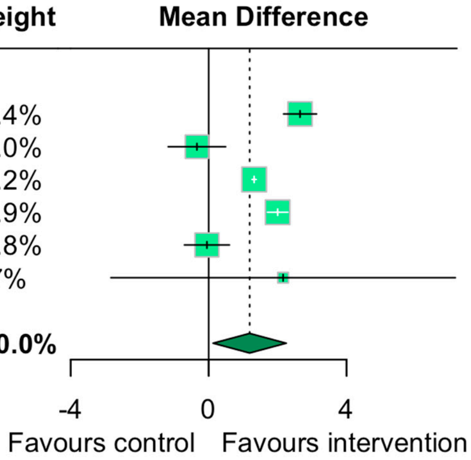

Figure S17. Forest plot of the mean difference (MD) with 95% Confidence Interval (CI) of TBUT results (s) for immunomodulatory drugs vs. artificial tears (AT). Other abbreviations: CsA, cyclosporine.

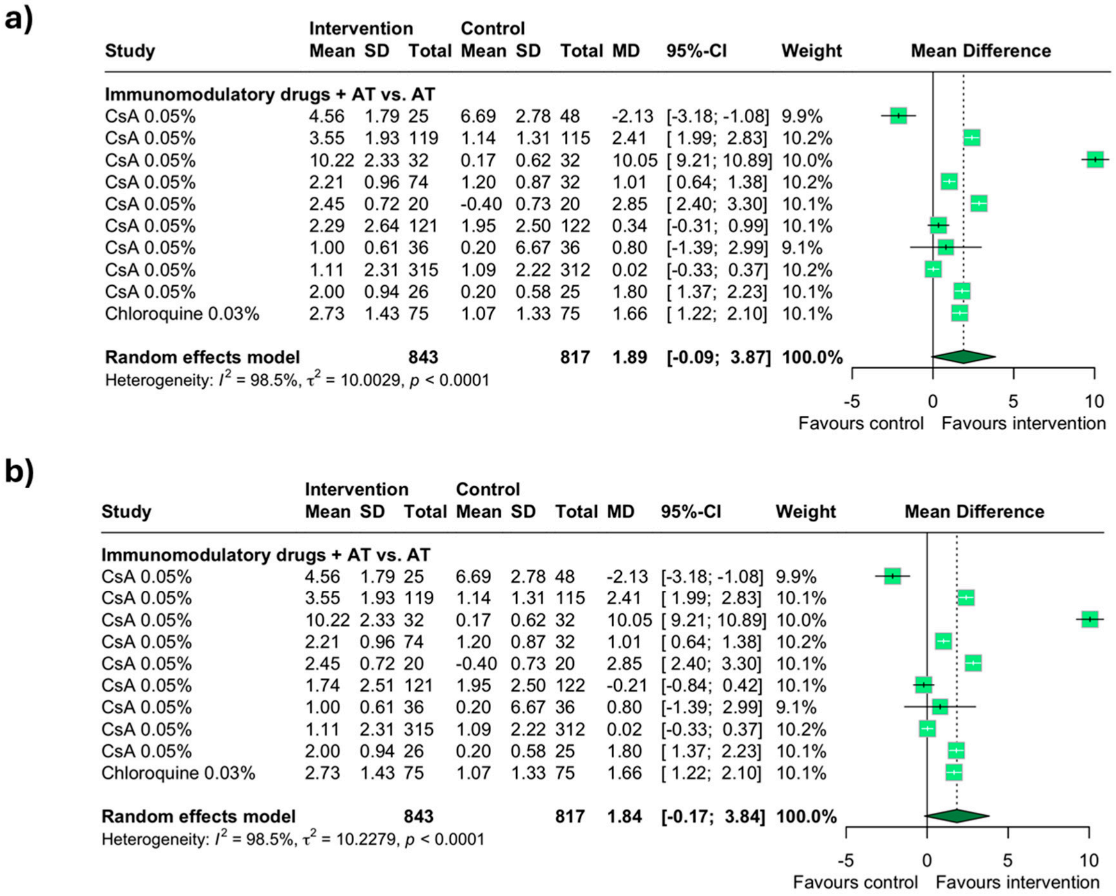

Figure S18. Forest plot of the mean difference (MD) with 95% Confidence Interval (CI) of TBUT results (s) for the combination of immunomodulatory drugs with artificial tears (AT) vs. AT. a) Analysis including one of the two intervention arms that shared the same control group; b) analysis including the other intervention arm. Other abbreviations: CsA, cyclosporine.

Study	Intervention Mean	Intervention SD	Intervention Total	Control Mean	Control SD	Control Total	MD	95%-CI	Weight
**Immunomodulatory drugs + AT vs. AT**									
CsA 0.05%	3.55	1.93	119	1.14	1.31	115	2.41	[1.99; 2.83]	11.3%
CsA 0.05%	10.22	2.33	32	0.17	0.62	32	10.05	[9.21; 10.89]	11.1%
CsA 0.05%	2.21	0.96	74	1.20	0.87	32	1.01	[0.64; 1.38]	11.3%
CsA 0.05%	2.45	0.72	20	-0.40	0.73	20	2.85	[2.40; 3.30]	11.3%
CsA 0.05%	1.74	2.51	121	1.95	2.50	122	-0.21	[-0.84; 0.42]	11.2%
CsA 0.05%	1.00	0.61	36	0.20	6.67	36	0.80	[-1.39; 2.99]	10.0%
CsA 0.05%	1.11	2.31	315	1.09	2.22	312	0.02	[-0.33; 0.37]	11.3%
CsA 0.05%	2.00	0.94	26	0.20	0.58	25	1.80	[1.37; 2.23]	11.3%
Chloroquine 0.03%	2.73	1.43	75	1.07	1.33	75	1.66	[1.22; 2.10]	11.3%
**Random effects model**			**818**			**769**	**2.27**	**[0.25; 4.29]**	**100.0%**

Heterogeneity: $I^2 = 98.6\%$, $\tau^2 = 9.3704$, $p < 0.0001$

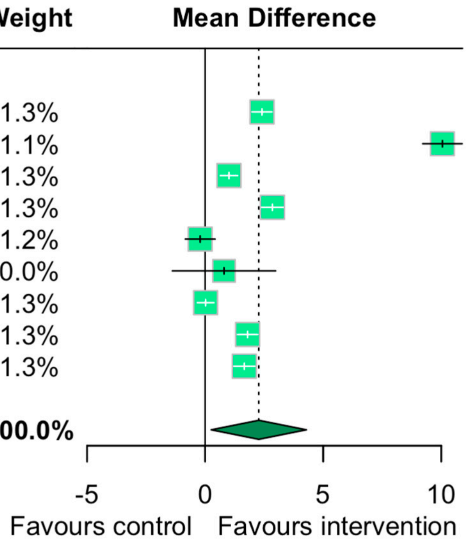

Figure S19. Forest plot of the mean difference (MD) with 95% Confidence Interval (CI) of TBUT results (s) for the combination of immunomodulatory drugs with artificial tears (AT) vs. AT. This analysis includes the intervention arm not incorporated in the main meta-analysis. Other abbreviations: CsA, cyclosporine.

Study	Intervention Mean	Intervention SD	Intervention Total	Control Mean	Control SD	Control Total	MD	95%-CI	Weight
**Follow-up: 6-8 weeks**									
CsA 0.05%	1.00	0.61	36	0.20	6.67	36	0.80	[-1.39; 2.99]	6.2%
**Follow-up: 12 weeks**									
CsA 0.05%	3.55	1.93	119	1.14	1.31	115	2.41	[1.99; 2.83]	13.5%
CsA 0.05%	2.21	0.96	74	1.20	0.87	32	1.01	[0.64; 1.38]	13.6%
CsA 0.05%	2.45	0.72	20	-0.40	0.73	20	2.85	[2.40; 3.30]	13.4%
CsA 0.05%	2.29	2.64	121	1.95	2.50	122	0.34	[-0.31; 0.99]	12.7%
CsA 0.05%	1.11	2.31	315	1.09	2.22	312	0.02	[-0.33; 0.37]	13.7%
CsA 0.05%	2.00	0.94	26	0.20	0.58	25	1.80	[1.37; 2.23]	13.5%
Chloroquine 0.03%	2.73	1.43	75	1.07	1.33	75	1.66	[1.22; 2.10]	13.4%
**Random effects model**			**750**			**701**	**1.45**	**[0.67; 2.22]**	**93.8%**
Heterogeneity: $I^2 = 95.7\%$, $\tau^2 = 1.0306$, $p < 0.0001$									
**Random effects model**			**786**			**737**	**1.41**	**[0.68; 2.13]**	**100.0%**
Heterogeneity: $I^2 = 95.0\%$, $\tau^2 = 0.9622$, $p < 0.0001$									

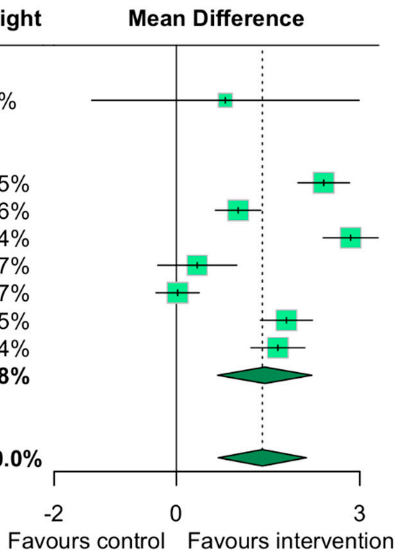

Figure S20. Forest plot of the mean difference (MD) with 95% Confidence Interval (CI) of TBUT results (s) for the combination of immunomodulatory drugs with artificial tears (AT) vs. AT, stratified by follow-up. Other abbreviations: CsA, cyclosporine.

Study	Intervention Mean	Intervention SD	Intervention Total	Control Mean	Control SD	Control Total	MD	95%-CI	Weight
**Mild to moderate DED**									
Chloroquine 0.03%	2.73	1.43	75	1.07	1.33	75	1.66	[1.22; 2.10]	13.4%
**Moderate to severe DED**									
CsA 0.05%	1.00	0.61	36	0.20	6.67	36	0.80	[-1.39; 2.99]	6.2%
CsA 0.05%	3.55	1.93	119	1.14	1.31	115	2.41	[1.99; 2.83]	13.5%
CsA 0.05%	2.29	2.64	121	1.95	2.50	122	0.34	[-0.31; 0.99]	12.7%
CsA 0.05%	1.11	2.31	315	1.09	2.22	312	0.02	[-0.33; 0.37]	13.7%
**Random effects model**			**591**			**585**	**0.91**	**[-0.29; 2.11]**	**46.0%**
Heterogeneity: I^2 = 96%, τ^2 = 1.2617, p < 0.0001									
**DED + SS or MGD**									
CsA 0.05%	2.21	0.96	74	1.20	0.87	32	1.01	[0.64; 1.38]	13.6%
CsA 0.05%	2.45	0.72	20	-0.40	0.73	20	2.85	[2.40; 3.30]	13.4%
CsA 0.05%	2.00	0.94	26	0.20	0.58	25	1.80	[1.37; 2.23]	13.5%
**Random effects model**			**120**			**77**	**1.88**	**[0.84; 2.93]**	**40.5%**
Heterogeneity: I^2 = 94.8%, τ^2 = 0.8072, p < 0.0001									
**Random effects model**			**786**			**737**	**1.41**	**[0.68; 2.13]**	**100.0%**
Heterogeneity: I^2 = 95.0%, τ^2 = 0.9622, p < 0.0001									

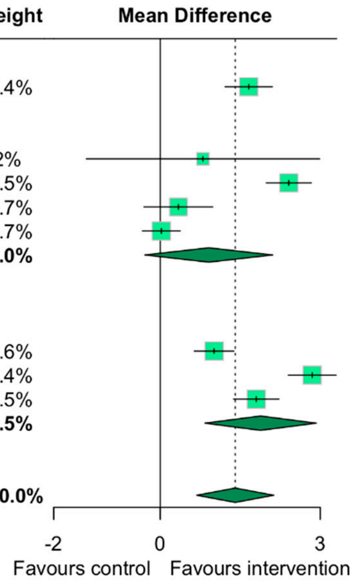

Figure S21. Forest plot of the mean difference (MD) with 95% Confidence Interval (CI) of TBUT results (s) for the combination of immunomodulatory drugs with artificial tears (AT) vs. AT, stratified by dry eye disease (DED) severity. Other abbreviations: CsA, cyclosporine; MGD, Meibomian gland dysfunction; SS, Sjögren's syndrome.

a)

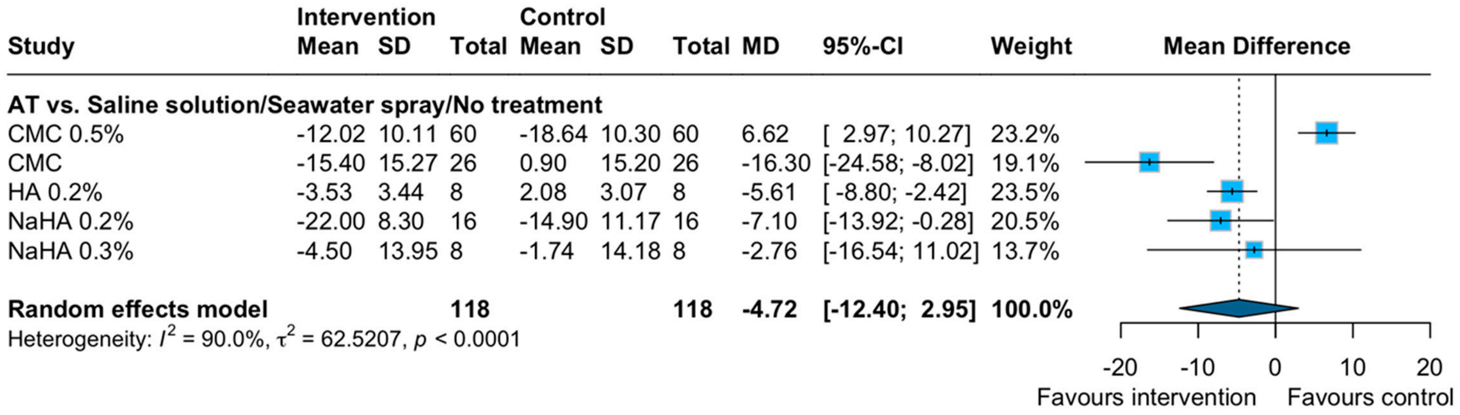

b)

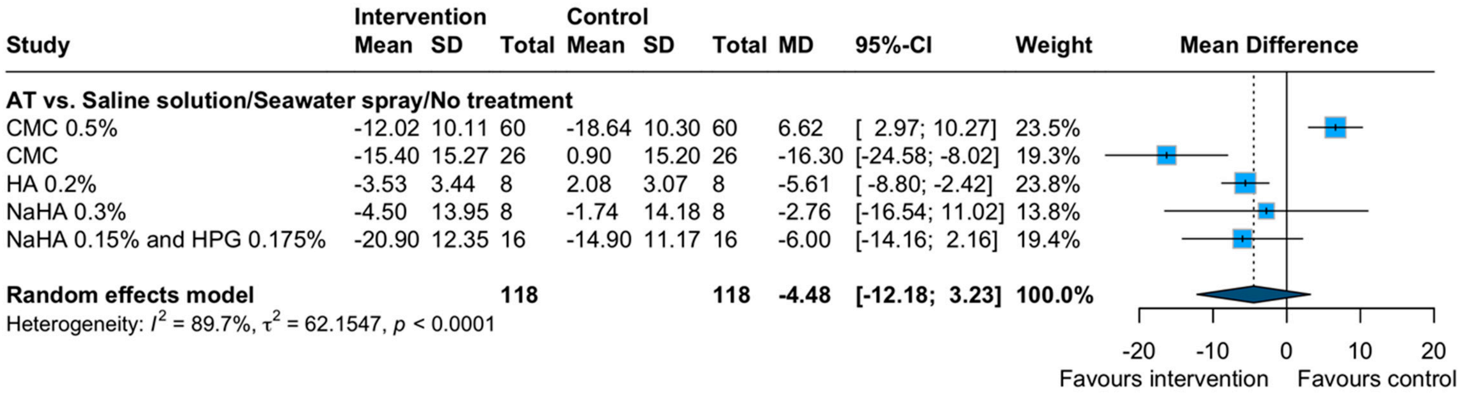

Figure S22. Forest plot of the mean difference (MD) with 95% Confidence Interval (CI) of Ocular Surface Disease Index (OSDI) results (score) for artificial tears (AT) vs. saline solution, seawater spray, or no treatment. a) Analysis including one of the two intervention arms that shared the same control group; b) analysis including the other intervention arm. Other abbreviations: CMC, carboxymethylcellulose; HA, hyaluronic acid; HPG, hydroxypropyl guar; NaHA, sodium hyaluronate.

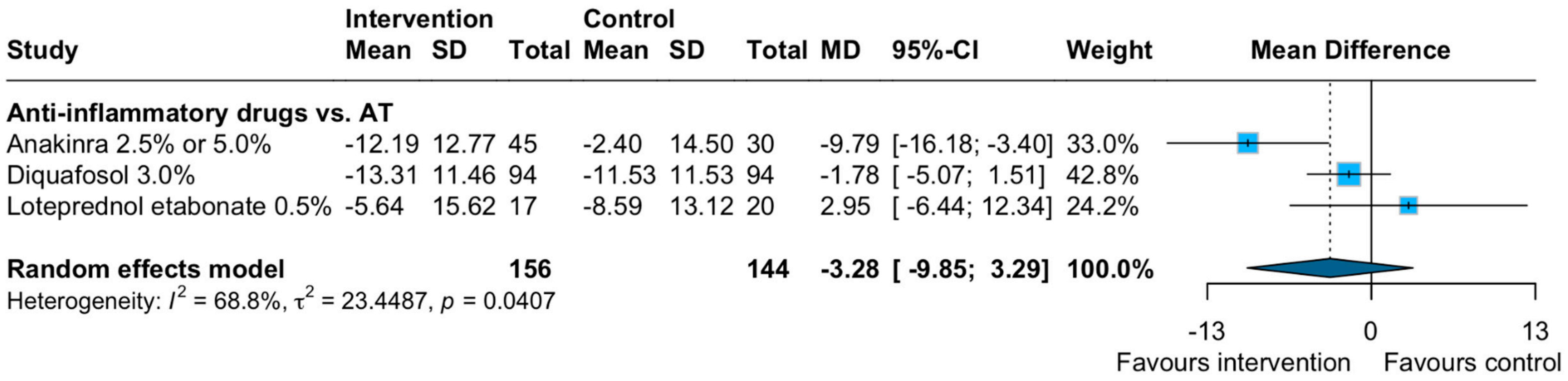

Figure S23. Forest plot of the mean difference (MD) with 95% Confidence Interval (CI) of OSDI scores for anti-inflammatory drugs vs. artificial tears (AT).

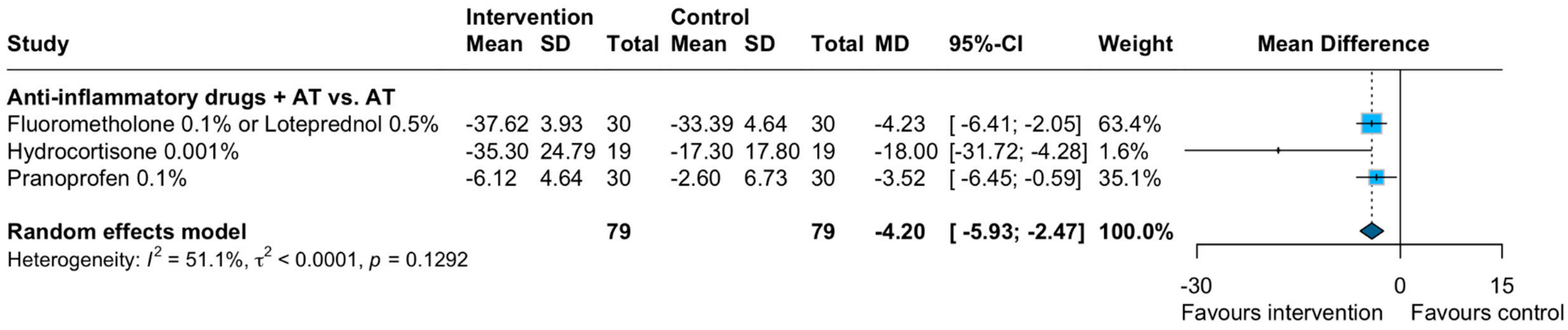

Figure S24. Forest plot of the mean difference (MD) with 95% Confidence Interval (CI) of Ocular Surface Disease Index (OSDI) results (score) for the combination of anti-inflammatory drugs and artificial tears (AT) vs. AT in the sensitivity analysis excluding the study on diquafosol.

a)

Study	Intervention Mean	Intervention SD	Intervention Total	Control Mean	Control SD	Control Total	MD	95%-CI	Weight
**Immunomodulatory drugs vs. Vehicle**									
CsA 0.05%	-4.90	17.38	44	-11.10	17.58	45	6.20	[-1.06; 13.46]	38.8%
CsA 0.1%	-14.40	21.10	131	-13.30	18.80	82	-1.10	[-6.54; 4.34]	41.7%
Lifitegrast 5.0%	-13.70	26.50	10	5.50	13.00	6	-19.20	[-38.64; 0.24]	19.5%
**Random effects model**			**185**			**133**	**-1.81**	**[-13.13; 9.51]**	**100.0%**

Heterogeneity: $I^2 = 69.9\%$, $\tau^2 = 72.3358$, $p = 0.0361$

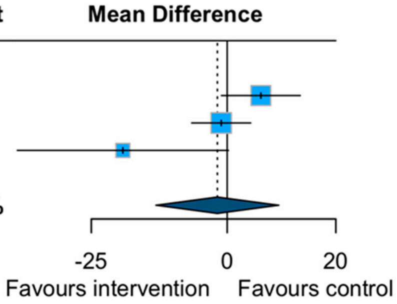

b)

Study	Intervention Mean	Intervention SD	Intervention Total	Control Mean	Control SD	Control Total	MD	95%-CI	Weight
**Immunomodulatory drugs vs. Vehicle**									
CsA 0.1%	-14.40	21.10	131	-13.30	18.80	82	-1.10	[-6.54; 4.34]	49.6%
Lifitegrast 5.0%	-13.70	26.50	10	5.50	13.00	6	-19.20	[-38.64; 0.24]	3.9%
Tofacitinib 0.0003%, 0.001%, 0.003% or 0.005%	-9.50	17.71	230	-11.10	17.58	45	1.60	[-4.02; 7.22]	46.5%
**Random effects model**			**371**			**133**	**-0.55**	**[-4.38; 3.28]**	**100.0%**

Heterogeneity: $I^2 = 51.6\%$, $\tau^2 < 0.0001$, $p = 0.1264$

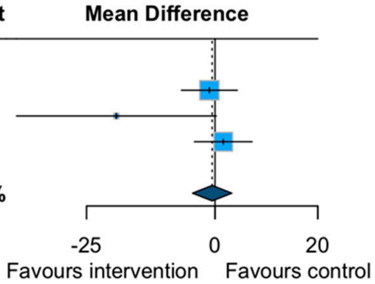

Figure S25. Forest plot of the mean difference (MD) with 95% Confidence Interval (CI) of OSDI scores for immunomodulatory drugs vs. vehicle. a) Analysis including one of the two intervention arms that shared the same control group; b) analysis including the other intervention arm. Other abbreviations: CsA, cyclosporine.

Study	Intervention Mean	SD	Total	Control Mean	SD	Total	MD	95%-CI	Weight
**Immunomodulatory drugs vs. AT**									
CsA 0.05%	-18.10	5.19	20	-1.40	6.25	20	-16.70	[-20.26; -13.14]	25.4%
CsA 0.05%	-17.93	20.58	45	-12.11	17.59	131	-5.82	[-12.55; 0.91]	22.4%
CsA 0.05%	-4.00	2.56	36	0.60	1.24	22	-4.60	[-5.58; -3.62]	26.7%
CsA 0.05%	-9.41	12.14	94	-11.53	11.53	94	2.12	[-1.26; 5.50]	25.5%
**Random effects model**			**195**			**267**	**-6.23**	**[-14.02; 1.55]**	**100.0%**

Heterogeneity: $I^2 = 94.9\%$, $\tau^2 = 58.8113$, $p < 0.0001$

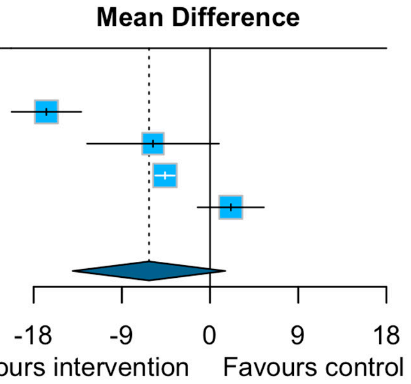

Figure S26. Forest plot of the mean difference (MD) with 95% Confidence Interval (CI) of OSDI scores for immunomodulatory drugs vs. artificial tears (AT). Other abbreviations: CsA, cyclosporine.

Study	Intervention Mean	Intervention SD	Intervention Total	Control Mean	Control SD	Control Total	MD	95%-CI	Weight
**Follow-up: 12 weeks**									
Chloroquine 0.03%	-9.36	3.04	75	-5.40	3.10	75	-3.96	[-4.94; -2.98]	18.8%
CsA 0.05%	-17.50	6.60	20	-1.40	6.25	20	-16.10	[-20.08; -12.12]	16.1%
CsA 0.05%	-20.65	9.31	74	-13.26	8.95	32	-7.39	[-11.15; -3.63]	16.4%
CsA 0.05%	-11.00	11.91	121	-9.30	10.86	122	-1.70	[-4.57; 1.17]	17.4%
CsA 0.05%	-21.40	11.58	26	-19.00	11.40	25	-2.40	[-8.71; 3.91]	13.1%
**Random effects model**			**316**			**274**	**-6.32**	**[-11.44; -1.19]**	**81.7%**
Heterogeneity: $I^2 = 90\%$, $\tau^2 = 30.3478$, $p < 0.0001$									
**Follow-up: 24 weeks**									
CsA 0.05%	-13.95	3.55	32	-6.40	3.87	32	-7.55	[-9.37; -5.73]	18.3%
**Random effects model**			**348**			**306**	**-6.54**	**[-10.65; -2.42]**	**100.0%**
Heterogeneity: $I^2 = 89.8\%$, $\tau^2 = 23.1998$, $p < 0.0001$									

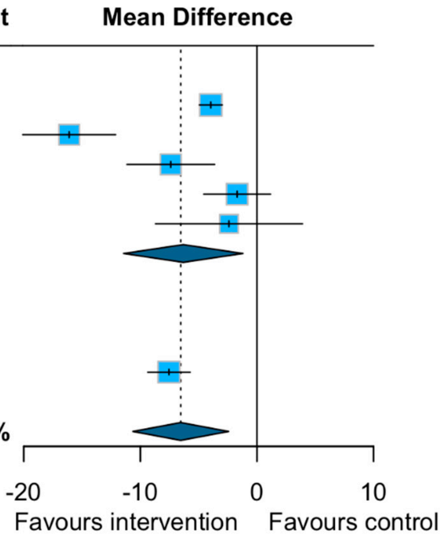

Figure S27. Forest plot of the mean difference (MD) with 95% Confidence Interval (CI) of OSDI scores for the combination of immunomodulatory drugs with artificial tears (AT) vs. AT, stratified by follow-up. Other abbreviations: CsA, cyclosporine.

Study	Intervention Mean	Intervention SD	Intervention Total	Control Mean	Control SD	Control Total	MD	95%-CI	Weight
**Mild to moderate DED**									
Chloroquine 0.03%	-9.36	3.04	75	-5.40	3.10	75	-3.96	[-4.94; -2.98]	18.8%
**Moderate to severe DED**									
CsA 0.05%	-11.00	11.91	121	-9.30	10.86	122	-1.70	[-4.57; 1.17]	17.4%
CsA 0.05%	-13.95	3.55	32	-6.40	3.87	32	-7.55	[-9.37; -5.73]	18.3%
**Random effects model**			**153**			**154**	**-4.73**	**[-10.46; 0.99]**	**35.7%**
Heterogeneity: $I^2 = 91.2\%$, $\tau^2 = 15.6108$, $p = 0.0007$									
**DED + SS or MGD**									
CsA 0.05%	-17.50	6.60	20	-1.40	6.25	20	-16.10	[-20.08; -12.12]	16.1%
CsA 0.05%	-20.65	9.31	74	-13.26	8.95	32	-7.39	[-11.15; -3.63]	16.4%
CsA 0.05%	-21.40	11.58	26	-19.00	11.40	25	-2.40	[-8.71; 3.91]	13.1%
**Random effects model**			**120**			**77**	**-8.89**	**[-16.64; -1.14]**	**45.6%**
Heterogeneity: $I^2 = 87.8\%$, $\tau^2 = 41.0313$, $p = 0.0003$									
**Random effects model**			**348**			**306**	**-6.54**	**[-10.65; -2.42]**	**100.0%**
Heterogeneity: $I^2 = 89.8\%$, $\tau^2 = 23.1998$, $p < 0.0001$									

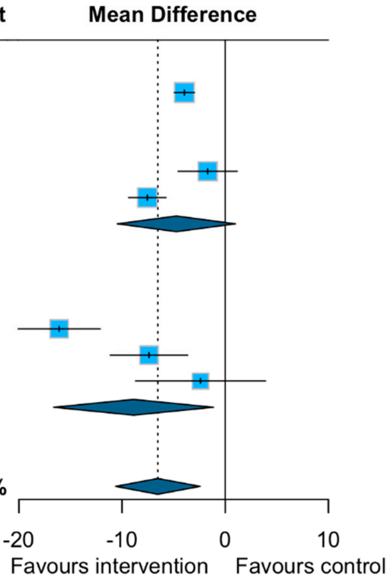

Figure S28. Forest plot of the mean difference (MD) with 95% Confidence Interval (CI) of OSDI scores for the combination of immunomodulatory drugs with artificial tears (AT) vs. AT, stratified by dry eye disease (DED) severity. Other abbreviations: CsA, cyclosporine; MGD, Meibomian gland dysfunction; SS, Sjögren's syndrome.

Study	Intervention Mean	Intervention SD	Intervention Total	Control Mean	Control SD	Control Total	MD	95%-CI	Weight
**Immunomodulatory drugs + AT vs. AT**									
CsA 0.05%	-13.95	3.55	32	-6.40	3.87	32	-7.55	[-9.37; -5.73]	17.9%
CsA 0.05%	-17.50	6.60	20	-1.40	6.25	20	-16.10	[-20.08; -12.12]	16.3%
CsA 0.05%	-20.65	9.31	74	-13.26	8.95	32	-7.39	[-11.15; -3.63]	16.5%
CsA 0.05%	-7.72	10.44	121	-9.30	10.86	122	1.58	[-1.10; 4.26]	17.3%
CsA 0.05%	-21.40	11.58	26	-19.00	11.40	25	-2.40	[-8.71; 3.91]	13.9%
Chloroquine 0.03%	-9.36	3.04	75	-5.40	3.10	75	-3.96	[-4.94; -2.98]	18.2%
**Random effects model**			**348**			**306**	**-5.96**	**[-10.74; -1.19]**	**100.0%**

Heterogeneity: $I^2 = 92.5\%$, $\tau^2 = 32.3766$, $p < 0.0001$

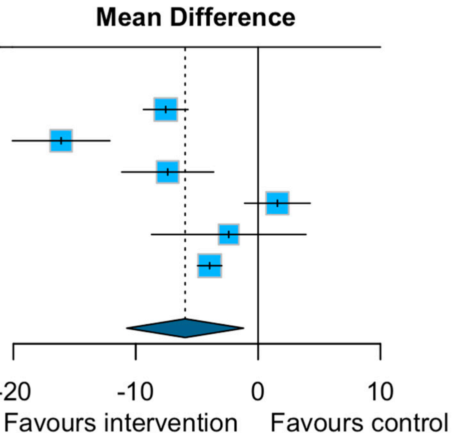

Figure S29. Forest plot of the mean difference (MD) with 95% Confidence Interval (CI) of OSDI scores for the combination of immunomodulatory drugs with artificial tears (AT) vs. AT. Other abbreviations: CsA, cyclosporine.

a)

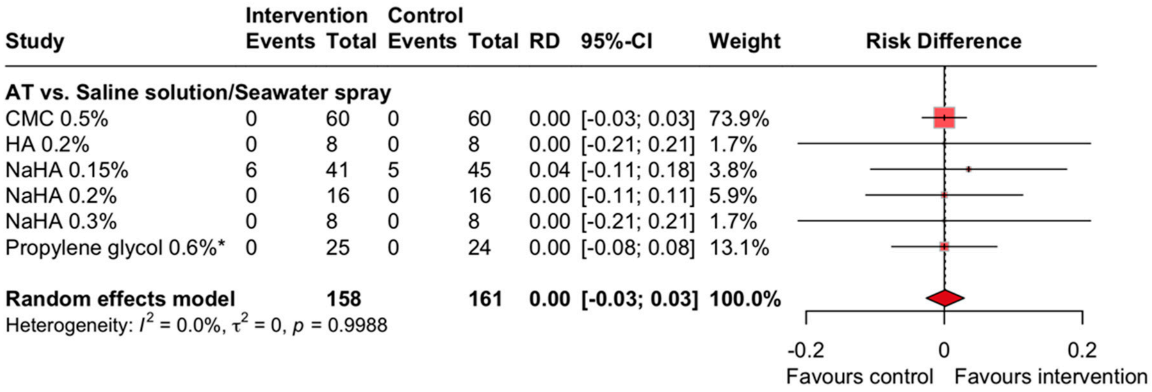

b)

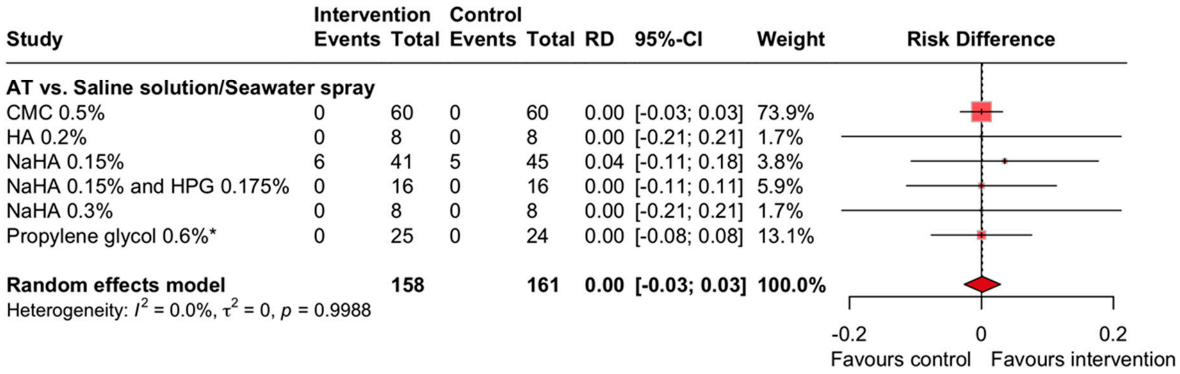

Figure S30. Forest plot of the risk difference (RD) with 95% Confidence Interval (CI) for any adverse events (AEs) in studies comparing artificial tears (AT) with saline solution or seawater spray. a) Analysis including one of the two intervention arms that shared the same control group; b) analysis including the other intervention arm. Other abbreviations: CMC, carboxymethylcellulose; HA, hyaluronic acid; HPG, hydroxypropyl guar; NaHA, sodium hyaluronate.

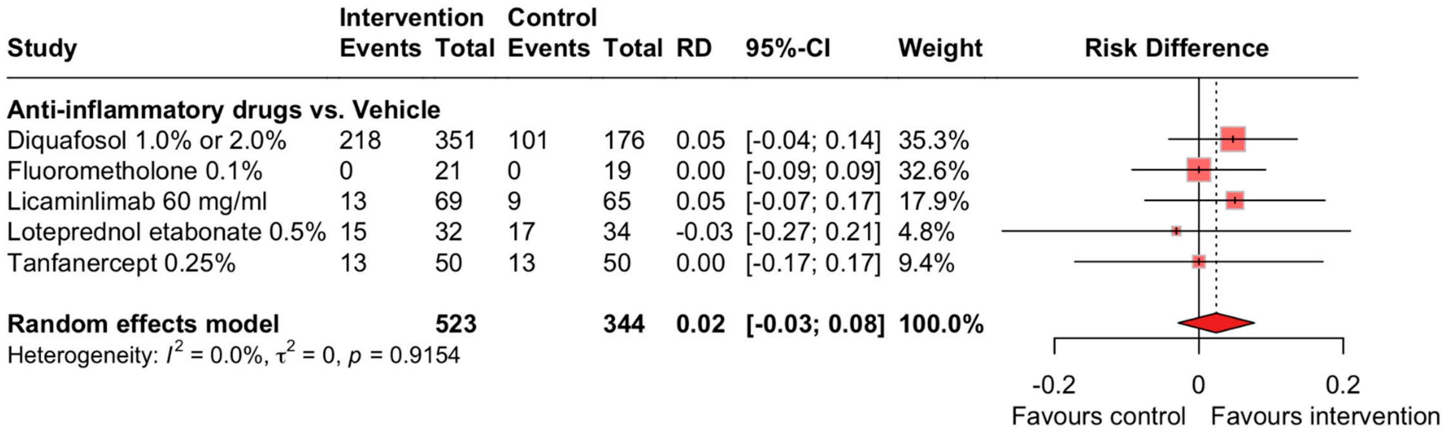

Figure S31. Forest plot of the risk difference (RD) with 95% Confidence Interval (CI) for any AEs in studies comparing anti-inflammatory drugs with vehicle.

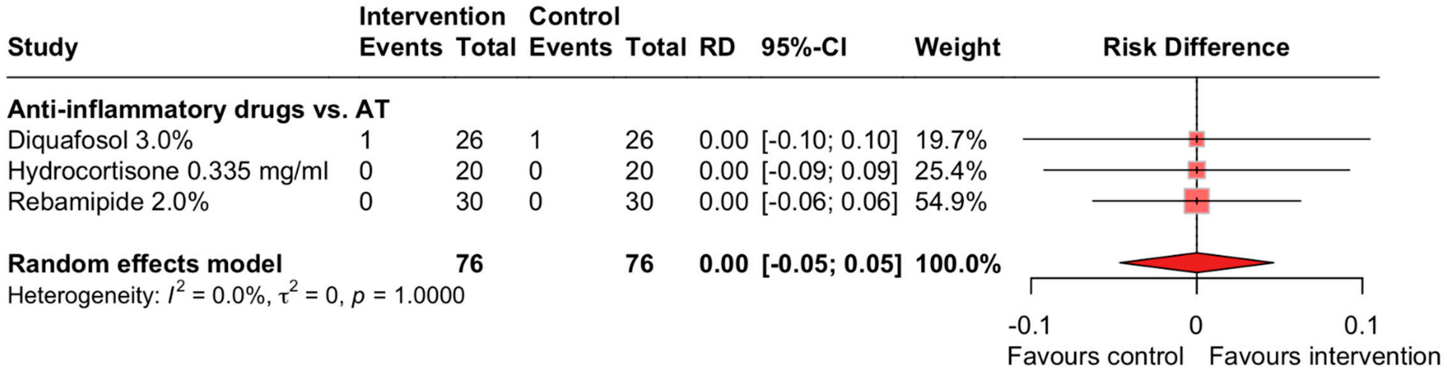

Figure S32. Forest plot of the risk difference (RD) with 95% Confidence Interval (CI) for any AEs in studies comparing anti-inflammatory drugs with artificial tears (AT).

Study	Intervention Events	Intervention Total	Control Events	Control Total	RD	95%-CI	Weight
**Anti-inflammatory drugs + AT vs. AT**							
Fluorometholone 0.1% or Loteprednol 0.5%	11	30	13	30	-0.07	[-0.31; 0.18]	22.4%
Hydrocortisone 0.001%	0	19	0	19	0.00	[-0.10; 0.10]	41.7%
Pranoprofen 0.1%	16	55	5	60	0.21	[0.07; 0.35]	35.8%
**Random effects model**		**104**		**109**	**0.06**	**[-0.10; 0.22]**	**100.0%**
Heterogeneity: $I^2 = 70.6\%$, $\tau^2 = 0.0132$, $p = 0.0333$							

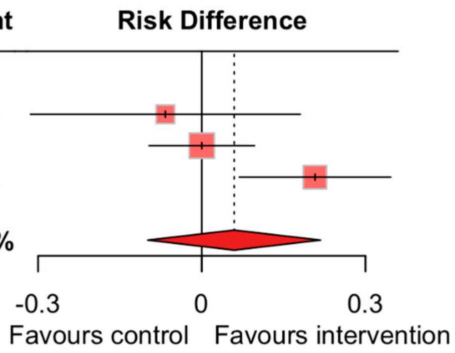

Figure S33. Forest plot of the risk difference (RD) with 95% Confidence Interval (CI) for any AEs in studies comparing a combination of anti-inflammatory drugs and artificial tears (AT) with AT alone.

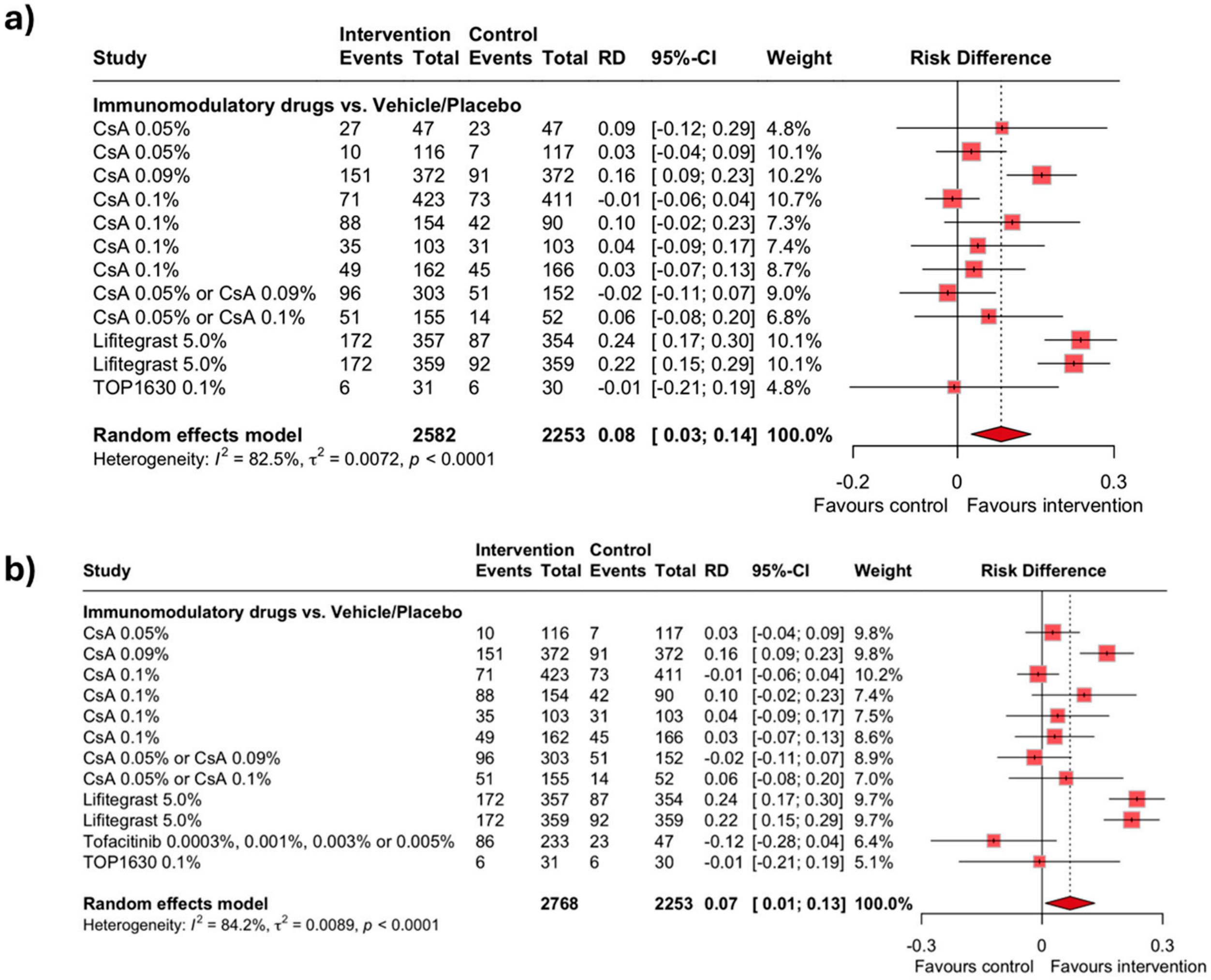

Figure S34. Forest plot of the risk difference (RD) with 95% Confidence Interval (CI) for any AEs in studies comparing immunomodulatory drugs with vehicle or placebo. a) Analysis including one of the two intervention arms that shared the same control group; b) analysis including the other intervention arm. Other abbreviations: CsA, cyclosporine.

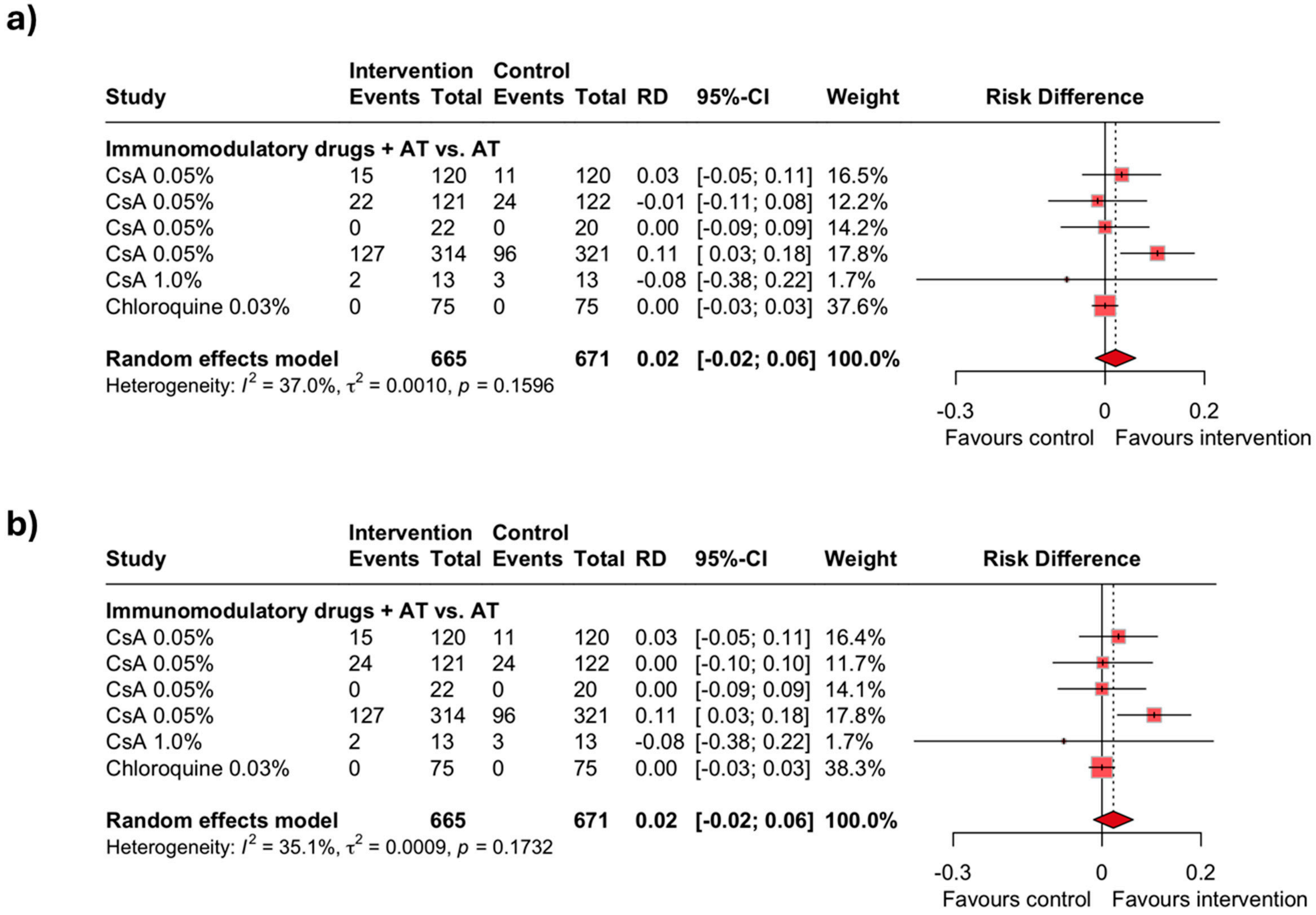

Figure S35. Forest plot of the risk difference (RD) with 95% Confidence Interval (CI) for any AEs in studies comparing a combination of immunomodulatory drugs and artificial tears (AT) with AT alone. a) Analysis including one of the two intervention arms that shared the same control group; b) analysis including the other intervention arm. Other abbreviations: CsA, cyclosporine.

a)

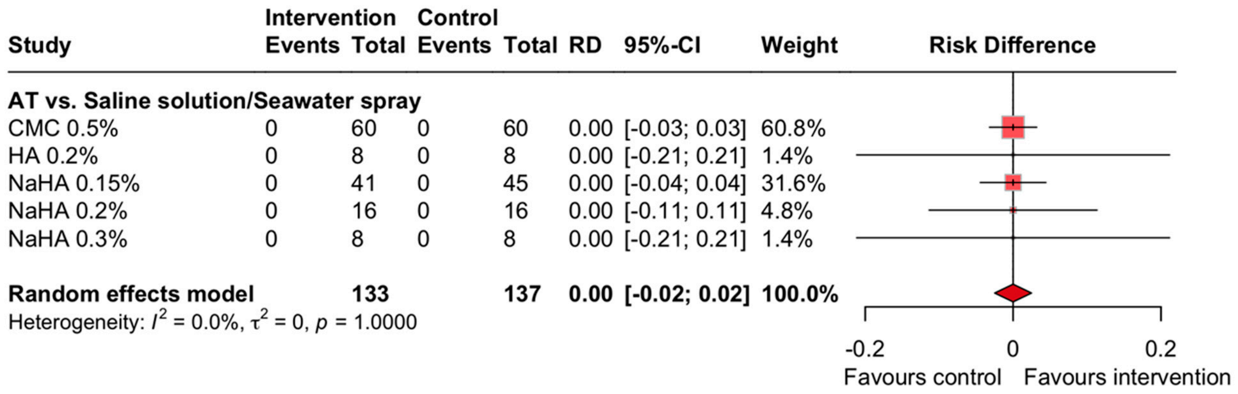

b)

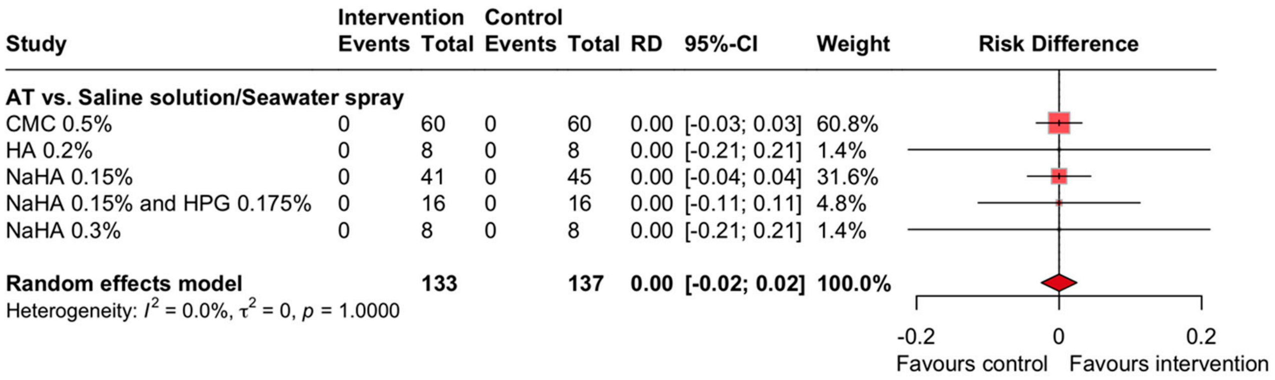

Figure S36. Forest plot of the risk difference (RD) with 95% Confidence Interval (CI) for treatment-related AEs in studies comparing artificial tears (AT) with saline solution or seawater spray. a) Analysis including one of the two intervention arms that shared the same control group; b) analysis including the other intervention arm. Other abbreviations: CMC, carboxymethylcellulose; HA, hyaluronic acid; HPG, hydroxypropyl guar; NaHA, sodium hyaluronate.

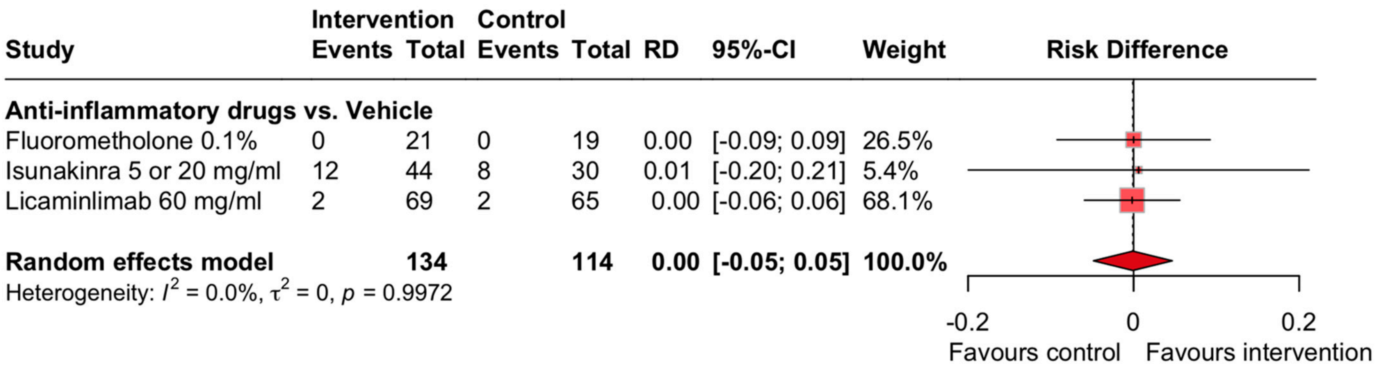

Figure S37. Forest plot of the risk difference (RD) with 95% Confidence Interval (CI) for treatment-related AEs in studies comparing anti-inflammatory drugs with vehicle.

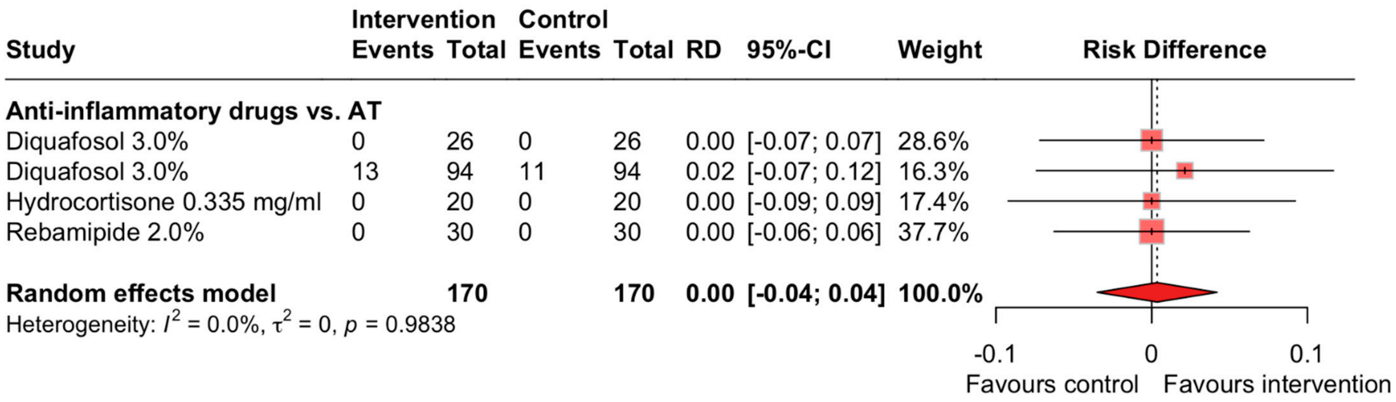

Figure S38. Forest plot of the risk difference (RD) with 95% Confidence Interval (CI) for treatment-related AEs in studies comparing anti-inflammatory drugs with artificial tears (AT).

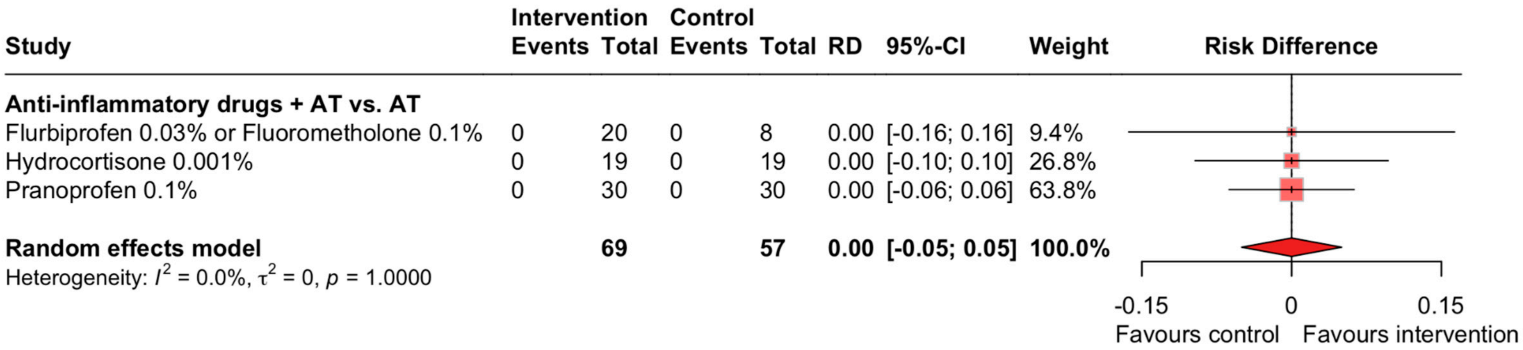

Figure S39. Forest plot of the risk difference (RD) with 95% Confidence Interval (CI) for treatment-related AEs in studies comparing a combination of anti-inflammatory drugs and artificial tears (AT) with AT alone.

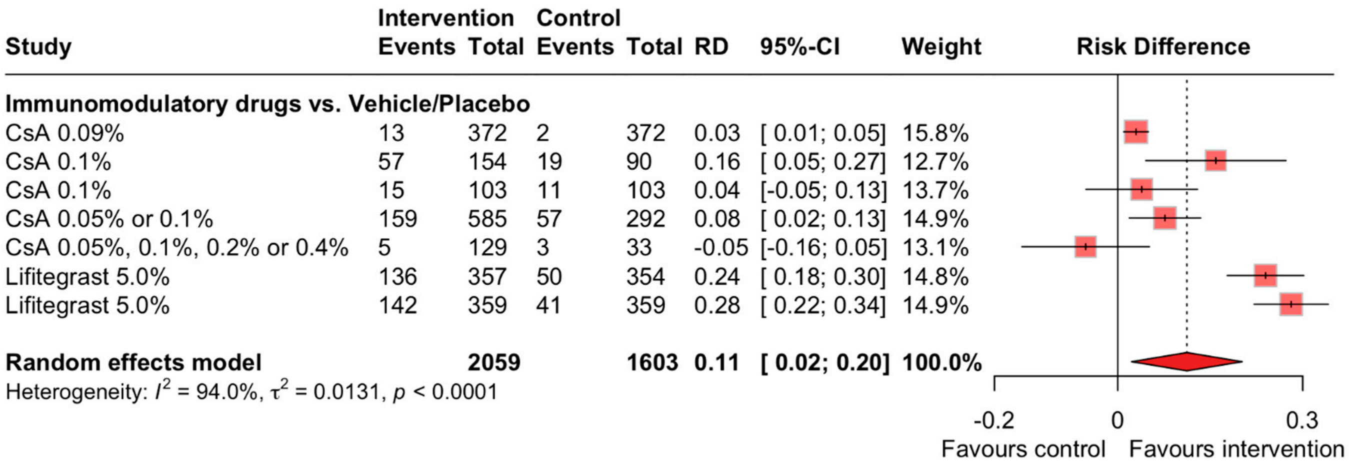

Figure S40. Forest plot of the risk difference (RD) with 95% Confidence Interval (CI) for treatment-related AEs in studies comparing immunomodulatory drugs with vehicle or placebo. Other abbreviations: CsA, cyclosporine.

a)

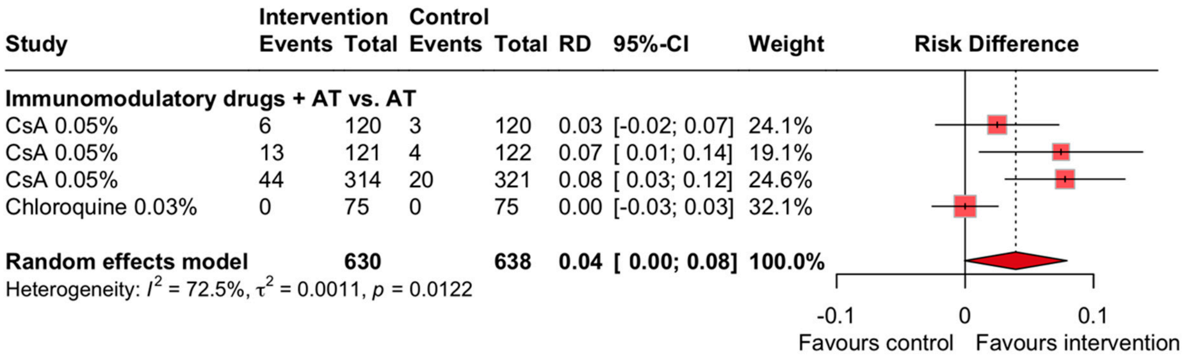

b)

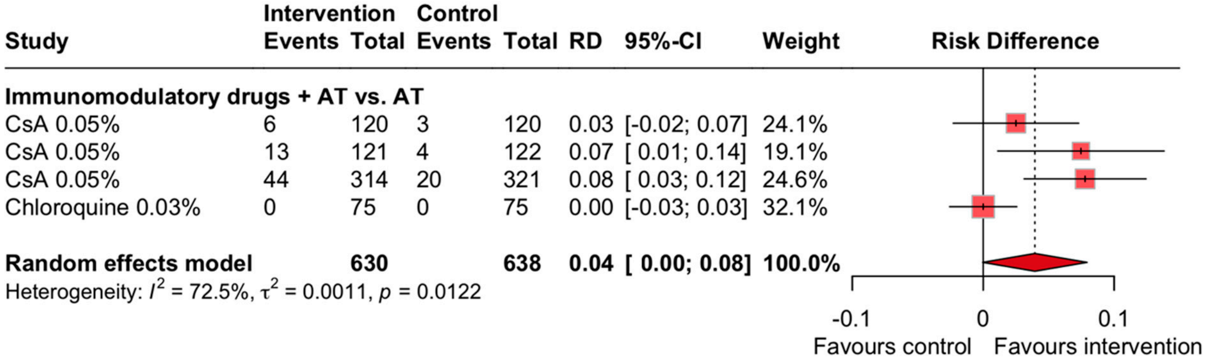

Figure S41. Forest plot of the risk difference (RD) with 95% Confidence Interval (CI) for treatment-related AEs in studies comparing a combination of immunomodulatory drugs and artificial tears (AT) with AT alone. a) Analysis including one of the two intervention arms that shared the same control group; b) analysis including the other intervention arm. Other abbreviations: CsA, cyclosporine.

Event	AT vs. vehicle/saline solution/placebo/ no treatment				Anti-inflammatory drugs vs. vehicle/saline solution				Anti-inflammatory drugs vs. AT				Immunomodulatory drugs vs. vehicle/saline solution				Immunomodulatory drugs vs. AT			
	Control		Intervention		Control		Intervention		Control		Intervention		Control		Intervention		Control		Intervention	
	N. of events/ patients (%*)	N. of studies	N. of events/ patients (%*)	N. of studies	N. of events/ patients (%*)	N. of studies	N. of events/ patients (%*)	N. of studies	N. of events/ patients (%*)	N. of studies	N. of events/ patients (%*)	N. of studies	N. of events/ patients (%*)	N. of studies	N. of events/ patients (%*)	N. of studies	N. of events/ patients (%*)	N. of studies	N. of events/ patients (%*)	N. of studies
Mild AEs	0/132	6	0/133	6	0/19	1	0/21	1	0/48	2	0/50	2	74/372 (19.9)	1	111/372 (29.8)	1	-	-	-	-
Moderate AEs	0/132	6	0/133	6	0/69	2	1/71 (1.4)	2	0/48	2	0/50	2	15/372 (4.0)	1	34/372 (9.1)	1	-	-	-	-
Severe AEs	0/132	6	0/133	6	1/49 (2.0)	2	1/65 (1.5)	2	0/166	4	0/183	4	0/52	1	0/155	1	0/94	1	0/94	1
Important AEs	0/132	6	0/133	6	0/19	1	0/21	1	0/48	2	0/50	2	-	-	-	-	-	-	-	-
Clinical AEs	0/132	6	0/133	6	0/19	1	0/21	1	0/48	2	0/50	2	-	-	-	-	-	-	-	-
Serious AEs	0/193	8	0/193	8	1/191 (0.5)	5	0/209	5	0/199	5	0/206	5	25/2257 (1.1)	12	30/2689 (1.1)	12	0/454	5	0/371	5
Treatment-related clinical AEs	0/227	8	0/224	8	0/19	1	0/21	1	0/74	3	0/76	3	-	-	-	-	-	-	-	-
Treatment-related serious AEs	0/349	11	0/346	11	0/317	6	0/560	6	0/249	7	0/277	7	1/2125 (0.1)	14	0/2743	14	0/454	5	0/371	5
Non-ocular AEs	0/132	6	0/133	6	10/69 (14.5)	2	9/71 (12.7)	2	2/72 (2.8)	3	2/95 (2.1)	3	154/1115 (13.8)	4	251/1261 (19.9)	4	-	-	-	-
Mild non-ocular AEs	0/132	6	0/133	6	0/19	1	0/21	1	0/48	2	0/50	2	48/713 (6.7)	2	133/716 (15.8)	2	-	-	-	-
Moderate non-ocular AEs	0/132	6	0/133	6	0/19	1	0/21	1	0/48	2	0/50	2	22/713 (3.1)	2	53/716 (7.4)	2	-	-	-	-
Severe non-ocular AEs	0/132	6	0/133	6	0/19	1	0/21	1	0/166	4	0/183	4	5/931 (0.5)	4	14/1033 (1.4)	4	0/94	1	0/94	1
Serious non-ocular AEs	0/193	8	0/193	8	1/191 (0.5)	5	0/209	5	0/199	5	0/206	5	11/1238 (0.9)	8	15/1454 (1.0)	8	0/454	5	0/371	5
Treatment-related non-ocular AEs	0/182	7	0/183	7	0/19	1	0/21	1	0/74	3	0/76	3	9/713 (1.3)	2	122/716 (17.0)	2	-	-	-	-
Mild treatment-related non-ocular AEs	0/227	8	0/224	8	0/19	1	0/21	1	0/74	3	0/76	3	5/359 (1.4)	1	39/359 (10.9)	1	-	-	-	-
Moderate treatment-related non-ocular AEs	0/227	8	0/224	8	0/19	1	0/21	1	0/74	3	0/76	3	1/359 (0.3)	1	28/359 (7.8)	1	-	-	-	-
Severe treatment-related non-ocular AE	0/227	8	0/224	8	0/19	1	0/21	1	0/192	5	0/209	5	0/411	2	3/214 (0.6)	2	0/94	1	0/94	1
Serious treatment-related non-ocular AEs	0/299	10	0/296	10	0/367	6	0/560	6	0/249	7	0/277	7	0/2093	14	0/2761	14	0/454	5	0/371	5

Discontinuation due to AEs	0/132	6	0/133	6	0/84	2	1/90 (1.1)	2	0/48	2	0/50	2	31/1549 (2.0)	9	91/2114 (4.3)	9	-	-	-	-
Discontinuation due to treatment-related AEs	0/227	8	0/224	8	0/84	2	0/90	2	0/74	3	0/76	3	-	-	-	-	-	-	-	-
Withdrawal due to AEs	2/193 (1.0)	7	4/195 (2.1)	7	8/275 (2.9)	4	11/466 (2.4)	4	3/220 (1.4)	5	4/214 (1.9)	5	31/1338 (2.3)	7	97/1669 (5.8)	7	0/175	1	1/176 (0.6)	1
Withdrawal due to non-ocular AEs	0/132	6	0/133	6	0/19	1	0/21	1	0/48	2	0/50	2	5/1008 (0.5)	3	15/1009 (1.5)	3	-	-	-	-
Withdrawal due to treatment-related AEs	0/227	8	0/224	9	0/19	1	0/21	1	0/74	3	0/76	3	2/372 (0.5)	1	13/372 (3.5)	1	-	-	-	-
Withdrawal due to drug intolerance	0/60	1	0/60	1	-		-	-	-	-	-	-	-	-	-	-	0/34	1	4/36 (11.1)	1
Intolerance/ poor tolerance	8/105 (7.6)	2	4/101 (4.0)	2	-	-	-	-	-	-	-	-	-	-	-	-	-	-	-	-
Death	0/132	6	0/133	6	0/84	2	0/90	2	0/142	3	0/144	3	0/976	9	0/1573	9	0/225	2	0/139	2

Table S2. General and non-ocular AEs not included in the meta-analysis in patients treated with artificial tears (AT), anti-inflammatory, or immunomodulatory drugs. Symbol legend: * percentage indicated only for values other than zero.

Event	Anti-inflammatory drugs vs. AT				Immunomodulatory drugs vs. AT			
	Control		Intervention		Control		Intervention	
	N. of events/ patients (%*)	N. of studies	N. of events/ patients (%*)	N. of studies	N. of events/ patients (%*)	N. of studies	N. of events/ patients (%*)	N. of studies
Mild AEs	0/19	1	0/19	1	0/95	2	0/97	2
Moderate AEs	0/19	1	0/19	1	0/95	2	0/97	2
Severe AEs	0/19	1	0/19	1	2/529 (0.4)	4	4/538 (0.7)	4
Important AEs	0/19	1	0/19	1	3/215 (1.4)	3	3/217 (1.4)	3
Clinical AEs	0/65	2	0/67	2	9/215 (4.2)	3	14/217 (6.5)	3
Serious AEs	0/89	3	0/82	3	4/693 (0.6)	7	6/700 (0.9)	7
Treatment-related clinical AEs	0/57	3	0/69	3	3/215 (1.4)	3	6/217 (2.8)	3
Treatment-related serious AEs	0/127	5	0/132	5	0/693	7	0/700	7
Non-ocular AEs	0/19	1	0/19	1	59/245 (13.9)	5	81/436 (18.6)	5
Mild non-ocular AEs	0/19	1	0/19	1	0/95	2	0/97	2
Moderate non-ocular AEs	0/19	1	0/19	1	0/95	2	0/97	2
Severe non-ocular AEs	0/19	1	0/19	1	1/529 (0.2)	4	2/538 (0.4)	4
Serious non-ocular AEs	0/89	3	0/82	3	2/465 (0.4)	5	6/476 (1.3)	5
Treatment-related non-ocular AEs	0/57	3	0/69	3	3/425 (0.7)	5	5/436 (1.2)	5
Mild treatment-related non-ocular AEs	0/57	3	0/69	3	0/95	2	0/97	2
Moderate treatment-related non-ocular AEs	0/57	3	0/69	3	0/95	2	0/97	2
Severe treatment-related non-ocular AE	0/57	3	0/69	3	0/215	3	0/217	3
Serious treatment-related	0/127	5	0/132	5	0/709	9	0/718	9

non-ocular AEs								
Discontinuation due to AEs	0/79	2	0/74	2	3/439 (0.7)	4	5/448 (1.1)	4
Discontinuation due to treatment-related AEs	0/57	3	0/69	3	0/125	3	0/127	3
Withdrawal due to AEs	0/19	1	0/19	1	2/497 (0.4)	7	4/504 (0.8)	7
Withdrawal due to non-ocular AEs	0/19	1	0/19	1	0/255	5	0/263	5
Withdrawal due to treatment-related AEs	0/57	3	0/69	3	0/95	2	0/97	2
Death	0/19	1	0/19	1	0/529	4	0/538	4

Table S3. General and non-ocular AEs not included in the meta-analysis in patients treated with a combination of anti-inflammatory or immunomodulatory drugs and artificial tears (AT). Symbol legend: * percentage indicated only for values other than zero.

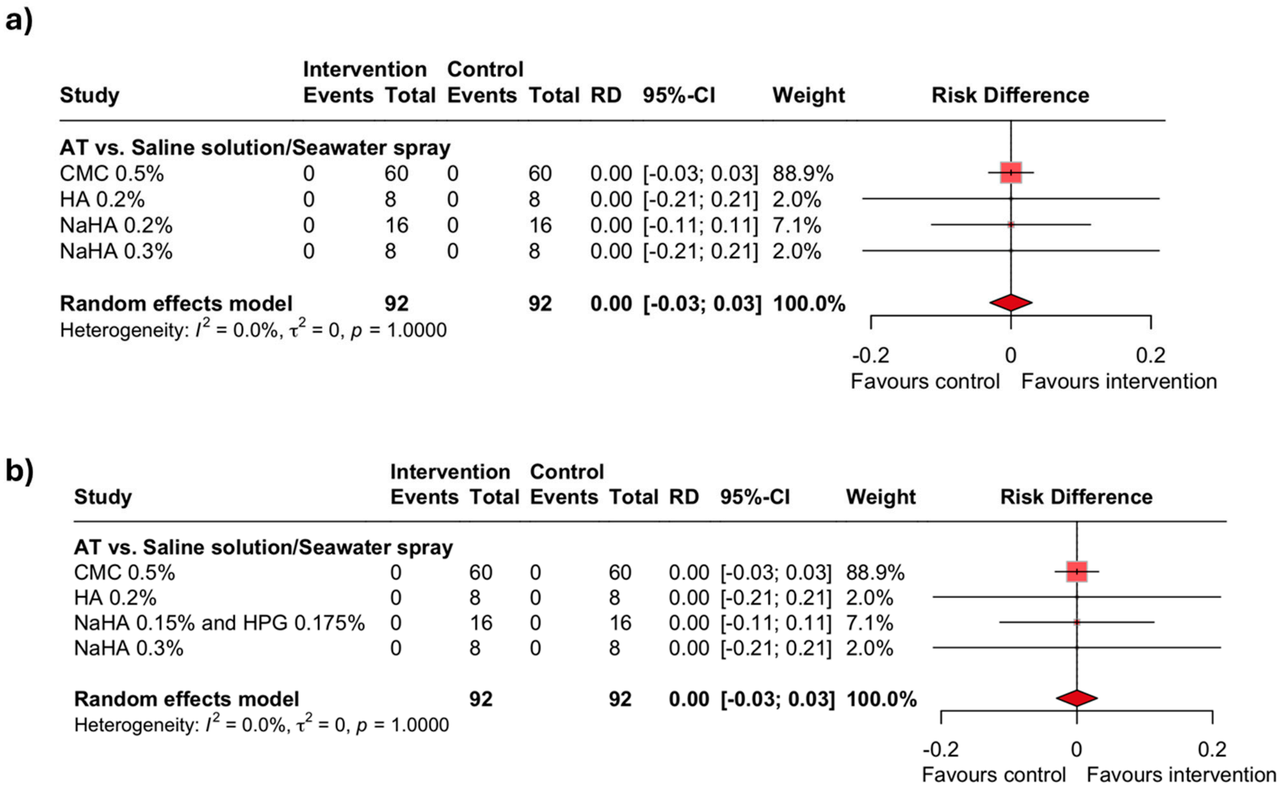

Figure S42. Forest plot of the risk difference (RD) with 95% Confidence Interval (CI) for any ocular AEs in studies comparing artificial tears (AT) with saline solution or seawater spray. a) Analysis including one of the two intervention arms that shared the same control group; b) analysis including the other intervention arm. Other abbreviations: CMC, carboxymethylcellulose; HA, hyaluronic acid; HPG, hydroxypropyl guar; NaHA, sodium hyaluronate.

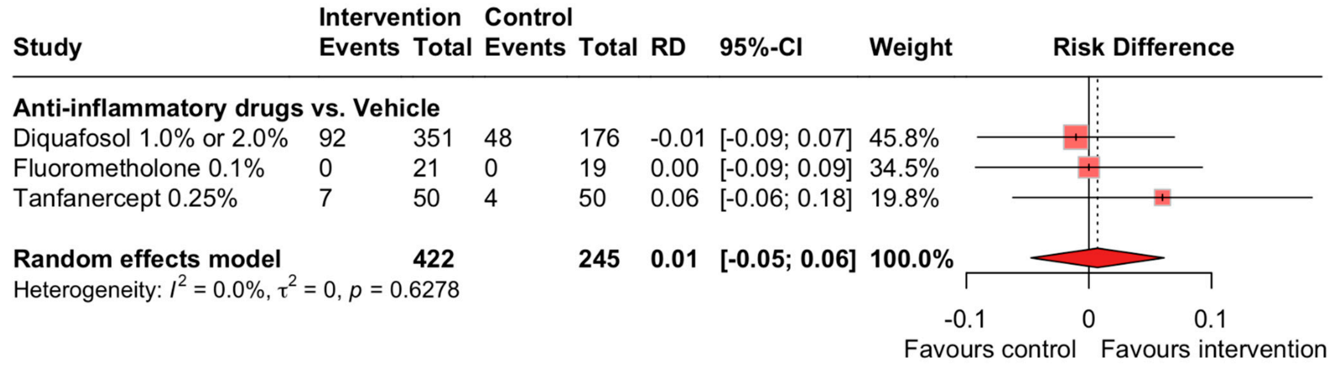

Figure S43. Forest plot of the risk difference (RD) with 95% Confidence Interval (CI) for any ocular AEs in studies comparing anti-inflammatory drugs with vehicle.

Event	AT vs. vehicle/saline solution/placebo/ no treatment				Anti-inflammatory drugs vs. vehicle/saline solution				Anti-inflammatory drugs vs. AT				Immunomodulatory drugs vs. vehicle/saline solution				Immunomodulatory drugs vs. AT			
	Control		Intervention		Control		Intervention		Control		Intervention		Control		Intervention		Control		Intervention	
	N. of events/ patients (%*)	N. of studies	N. of events/ patients (%*)	N. of studies	N. of events/ patients (%*)	N. of studies	N. of events/ patients (%*)	N. of studies	N. of events/ patients (%*)	N. of studies	N. of events/ patients (%*)	N. of studies	N. of events/ patients (%*)	N. of studies	N. of events/ patients (%*)	N. of studies	N. of events/ patients (%*)	N. of studies	N. of events/ patients (%*)	N. of studies
Mild ocular AEs	0/132	6	0/133	6	0/19	1	0/21	1	0/48	2	0/50	2	194/1160 (16.7)	6	489/1461 (33.5)	6	0/20	1	0/20	1
Moderate ocular AEs	0/132	6	0/133	6	0/19	1	0/21	1	0/48	2	0/50	2	40/1160 (3.5)	6	104/1461 (7.1)	6	0/20	1	0/20	1
Severe ocular AEs	0/132	6	0/133	6	0/19	1	0/21	1	0/166	4	0/183	4	49/1582 (3.1)	10	106/2043 (5.2)	10	0/114	2	0/114	2
Serious ocular AEs	0/193	8	0/193	8	0/367	6	0/560	6	0/199	5	0/206	5	1/2154	13	1/2680	13	0/454	5	0/371	5
Mild ocular treatment-related AEs	0/227	8	0/224	8	0/19	1	0/21	1	0/74	3	0/76	3	34/453 (7.5)	3	87/639 (13.6)	3	0/20	1	0/20	1
Moderate ocular treatment-related AEs	0/227	8	0/224	8	0/19	1	0/21	1	0/74	3	0/76	3	9/453 (2.0)	3	39/639 (6.1)	3	0/20	1	0/20	1
Severe treatment-related	0/227	8	0/224	8	0/19	1	0/21	1	0/192	5	0/209	5	28/755 (3.7)	5	95/1036 (9.2)	5	0/114	2	0/114	2

ocular AEs																				
Serious treatment-related ocular AEs	0/308	11	0/346	11	0/367	6	0/560	6	0/249	7	0/277	7	0/2394	14	0/2948	14	0/454	5	0/371	5
Discontinuation due to ocular AEs	1/177	7	1/177	7	0/19	1	0/21	1	0/48	2	0/50	2	26/539 (4.8)	4	46/687 (6.7)	4	0/36	2	2/39 (5.1)	2
Discontinuation due to intense burning sensation	0/132	6	0/132	6	0/19	1	0/21	1	0/48	2	0/50	2	-	-	-	-	0/20	1	0/20	1
Discontinuation due to instillation site pain	0/132	6	0/132	6	0/19	1	0/21	1	0/48	2	0/50	2	0/372	1	9/372 (2.4)	1	0/20	1	0/20	1
Discontinuation due to discomfort upon instillation	0/132	6	0/132	6	0/19	1	0/21	1	0/48	2	0/50	2	-	-	-	-	9/42 (21.4)	2	2/56 (3.6)	2
Discontinuation due to mild infection	1/177 (0.6)	7	1/177 (0.6)	7	0/19	1	0/21	1	0/48	2	0/50	2	-	-	-	-	0/20	1	0/20	1
Withdrawal due to ocular AEs	0/132	6	0/133	6	7/225 (3.1)	3	4/416 (1.0)	3	0/48	2	0/50	2	10/1008 (1.0)	3	46/1009 (4.6)	3	0/195	2	1/196 (0.5)	2
Withdrawal due to intolerable drug irritation	0/132	6	0/132	6	0/49	2	0/65	2	0/48	2	0/50	2	-	-	-	-	0/20	1	0/20	1
Visual acuity reduced	0/132	6	0/132	6	0/53	2	0/53	2	0/48	2	0/50	2	75/1591 (4.7)	9	75/1814 (4.1)	9	0/20	1	0/20	1
Mild instillation site reactions	0/132	6	0/133	6	0/19	1	0/21	1	0/48	2	0/50	2	35/411 (8.5)	1	42/423 (9.9)	1	0/20	1	0/20	1
Moderate instillation site reactions	0/132	6	0/133	6	0/19	1	0/21	1	0/48	2	0/50	2	1/411 (0.2)	1	1/423 (0.2)	1	0/20	1	0/20	1
Severe instillation site reactions	0/132	6	0/133	6	0/19	1	0/21	1	0/48	2	0/50	2	0/411 (0.2)	1	0/423 (0.2)	1	0/114	2	0/114	2
Blurred vision	0/132	6	0/132	6	0/19	1	0/21	1	0/48	2	0/50	2	15/1331 (1.1)	8	25/1708 (1.5)	8	0/20	1	0/20	1
Treatment-related blurred vision	0/227	8	0/224	8	0/53	2	2/53 (3.8)	2	2/98 (2.0)	4	3/121 (2.5)	4	8/90 (8.9)	2	45/275 (16.4)	2	0/20	1	0/20	1
Vitreous floaters	0/132	6	0/133	6	0/19	1	0/21	1	0/48	2	0/50	2	3/541 (0.6)	3	4/693 (0.6)	3	0/20	1	0/20	1
Vitreous detachment	0/132	6	0/133	6	0/19	1	0/21	1	0/48	2	0/50	2	0/511	2	4/662 (0.6)	2	0/20	1	0/20	1
Changes in IOP	0/148	7	0/149	7	0/53	2	0/53	2	0/48	2	0/50	2	1/33 (3.0)	1	0/129	1	0/20	1	0/20	1
Meibomian gland dysfunction	0/132	6	0/133	6	0/19	1	0/21	1	0/48	2	0/50	2	1/511 (0.2)	2	3/662 (0.5)	2	0/20	1	0/20	1

Treatment-related grittiness	0/227	8	0/224	8	0/19	1	0/21	1	0/98	4	3/121 (2.5)	4	-	-	-	-	0/20	1	0/20	1
Treatment-related epiphora	0/227	8	0/224	8	0/19	1	0/21	1	1/98 (1.0)	4	0/121	4	-	-	-	-	0/20	1	0/20	1
Conjunctivitis	0/132	6	0/133	6	0/69	2	3/71 (4.2)	2	1/72 (1.4)	3	2/95 (2.1)	3	5/563 (0.9)	3	3/817 (0.4)		0/20	1	0/20	1
Conjunctival redness	0/132	6	0/133	6	0/69	2	3/71 (4.2)	2	0/48	2	0/50	2	-	-	-	-	0/20	1	0/20	1
Conjunctival hemorrhage	0/132	6	0/133	6	0/19	1	0/21	1	0/48	2	0/50	2	3/621 (0.5)	4	15/989 (1.5)	4	0/20	1	0/20	1
Conjunctival edema	0/132	6	0/133	6	0/19	1	0/21	1	0/48	2	0/50	2	3/359 (0.8)	1	1/359 (0.3)	1	0/20	1	0/20	1
Eyelid irritation	0/132	6	0/133	6	0/19	1	0/21	1	2/72 (2.8)	3	1/95 (1.0)	3	-		-	-	0/20	1	0/20	1
Eyelid pain	0/132	6	0/133	6	0/19	1	0/21	1	0/48	2	0/50	2	0/152	1	2/303 (0.7)	1	0/20	1	0/20	1
Burning	0/132	6	0/133	6	0/19	1	0/21	1	0/48	2	0/50	2	-	-	-	-	0/20	1	0/20	1
Burning/ stinging	0/132	6	0/133	6	4/195 (2.0)	2	18/372 (4.8)	2	0/48	2	0/50	2	-	-	-	-	0/20	1	0/20	1
Treatment-related burning	0/227	8	0/224	8	4/53 (7.6)	2	2/53 (3.8)	2	3/98 (3.1)	4	1/121 (0.8)	4	35/412 (8.5)	4	153/888 (17.2)	4	0/20	1	0/20	1
Treatment-related stinging	0/227	8	0/224	8	0/19	1	0/21	1	1/98 (1.0)	4	1/121 (0.8)	4	4/322 (1.2)	2	23/613 (3.8)	2	0/20	1	0/20	1
Itching	0/132	6	0/133	6	0/19	1	0/21	1	0/48	2	0/50	2	-	-	-	-	0/20	1	0/20	1
Treatment-related itching	0/227	8	0/224	8	0/19	1	0/21	1	1/98 (1.0)	4	1/121 (0.8)	4	-	-	-	-	0/20	1	0/20	1
Pingueculitis	0/132	6	0/133	6	0/19	1	0/21	1	2/72 (2.8)	3	0/95	3	-	-	-	-	0/20	1	0/20	1
Blepharospasm	0/132	6	0/133	6	0/19	1	0/21	1	2/72 (2.8)	3	0/95	3	-	-	-	-	0/20	1	0/20	1
Episcleritis	0/132	6	0/133	6	0/19	1	0/21	1	0/72	3	1/95 (1.0)	3	-	-	-	-	0/20	1	0/20	1
Ocular pain/ eye pain	0/132	6	0/133	6	3/195 (1.5)	2	10/372 (2.7)	2	5/142 (3.5)	3	6/144 (4.2)	3	11/1019 (1.1)	6	20/1385 (1.4)	6	10/245 (4.1)	3	9/159 (5.7)	3
Mild ocular pain	0/132	6	0/133	6	0/19	1	0/21	1	0/48	2	0/50	2	-	-	-	-	0/20	1	0/20	1
Moderate ocular pain	0/132	6	0/133	6	0/19	1	0/21	1	0/48	2	0/50	2	-	-	-	-	0/20	1	0/20	1
Severe ocular pain	0/132	6	0/133	6	0/19	1	0/21	1	1/219 (0.5)	5	3/236 (1.3)	5	0/52	1	0/155	1	0/114	2	0/114	2
Treatment-related ocular pain/eye pain	0/227	8	0/224	8	0/49	2	2/65 (3.1)	2	0/74	3	0/76	3	15/632 (2.4)	4	52/1102 (4.7)	4	0/20	1	0/20	1
Eye irritation	0/132	6	0/133	6	0/49	2	1/65 (1.5)	2	0/48	2	0/50	2	9/935 (1.0)	4	20/1189 (1.7)	4	0/20	1	0/20	1
Treatment-related eye irritation	0/227	8	0/224	8	0/19	1	0/21	1	0/74	3	0/76	3	6/250 (2.4)	1	39/242 (16.1)	1	0/20	1	0/20	1
Vision loss	0/132	6	0/133	6	0/19	1	0/21	1	0/48	2	0/50	2	-	-	-	-	0/20	1	0/20	1
Abnormal sensation in eye	0/132	6	0/133	6	0/19	1	0/21	1	0/48	2	0/50	2	-	-	-	-	0/20	1	0/20	1
Asthenopia	0/132	6	0/133	6	0/19	1	0/21	1	0/48	2	0/50	2	-	-	-	-	0/20	1	0/20	1

Treatment-related redness	0/227	8	0/224	8	1/53 (1.9)	2	3/53 (5.7)	2	0/74	3	0/76	3	-	-	-	-	0/20	1	0/20	1
Eye pruritus	0/132	6	0/133	6	0/84	2	2/90 (2.2)	2	0/48	2	0/50	2	16/1236 (1.3)	5	17/1499 (1.1)	5	0/20	1	0/20	1
Treatment-related eye pruritus	0/227	8	0/224	8	0/84	2	1/90 (1.1)	2	0/74	3	0/76	3	-	-	-	-	0/20	1	0/20	1
Instillation site pruritus	0/132	6	0/133	6	0/19	1	0/21	1	0/48	2	0/50	2	6/654 (0.9)	2	27/652 (4.1)	2	0/20	1	0/20	1
Instillation site discomfort	0/132	6	0/133	6	0/19	1	0/21	1	0/48	2	0/50	2	1/30 (3.3)	1	0/31	1	0/20	1	0/20	1
Eye discharge	0/132	6	0/133	6	0/19	1	0/21	1	0/48	2	0/50	2	1/541 (0.2)	3	6/693 (0.9)	3	0/20	1	0/20	1
Treatment-related eye discharge	0/227	8	0/224	8	0/19	1	0/21	1	0/74	3	0/76	3	7/292 (2.4)	1	11/585 (1.9)	1	0/20	1	0/20	1
Foreign body sensation	2/148 (1.4)	7	2/149 (1.4)	7	0/49	2	1/65 (1.5)	2	0/48	2	0/50	2	6/731 (0.8)	2	2/731 (0.3)	2	0/20	1	0/20	1
Treatment-related foreign body sensation	0/227	8	0/224	8	3/53 (5.7)	2	1/53 (1.9)	2	0/74	3	0/76	3	6/292 (2.0)	1	12/585 (2.0)	1	0/20	1	0/20	1
Instillation site reaction	0/132	6	0/133	6	0/19	1	0/21	1	0/48	2	0/50	2	36/1693 (2.1)	7	180/1959 (9.2)	7	0/20	1	0/20	1
Treatment-related instillation site reaction	0/227	8	0/224	8	0/19	1	0/21	1	0/74	3	0/76	3	4/250 (1.6)	1	22/242 (9.0)	1	0/20	1	0/20	1
Instillation site irritation	0/132	6	0/133	6	0/19	1	0/21	1	0/48	2	0/50	2	28/1008 (2.8)	3	162/1009 (16.1)	3	0/20	1	0/20	1
Treatment-related instillation site irritation	0/227	8	0/224	8	0/19	1	0/21	1	0/74	3	0/76	3	-	-	-	-	0/20	1	0/20	1
Treatment-related visual disturbance	0/227	8	0/224	8	0/19	1	0/21	1	0/74	3	0/76	3	12/292 (4.1)	1	14/585 (2.4)	1	0/20	1	0/20	1
Increased lacrimation	0/132	6	0/133	6	0/49	2	1/65 (1.5)	2	0/48	2	0/50	2	3/1236 (0.2)	5	29/1499 (1.9)	5	0/20	1	0/20	1
Treatment-related increased lacrimation	0/227	8	0/224	8	0/84	2	1/90 (1.1)	2	0/74	3	0/76	3	5/340 (1.5)	3	45/517 (8.7)	3	0/20	1	0/20	1
Treatment-related eyelid erythema	0/227	8	0/224	8	0/19	1	0/21	1	0/74	3	0/76	3	5/250 (2.0)	1	9/242 (3.7)	1	0/20	1	0/20	1
Treatment-related meibomianitis	0/227	8	0/224	8	0/19	1	0/21	1	0/74	3	0/76	3	6/250 (2.4)	1	6/242 (2.5)	1	0/20	1	0/20	1
Conjunctival hyperemia	0/132	6	0/133	6	0/19	1	0/21	1	0/48	2	0/50	2	23/731 (3.2)	2	35/731 (4.8)	2	0/20	1	0/20	1
Mild conjunctival hyperemia	0/132	6	0/133	6	0/19	1	0/21	1	0/48	2	0/50	2	-	-	-	-	0/20	1	0/20	1
Treatment-related conjunctival hyperemia	0/227	8	0/224	8	0/19	1	0/21	1	0/74	3	0/76	3	5/542 (0.9)	2	21/827 (2.5)	2	0/20	1	0/20	1

Ocular hyperemia	0/132	6	0/133	6	0/49	2	1/65 (1.5)	2	0/48	2	0/50	2	6/712 (0.8)	3	13/824 (1.6)	3	0/20	1	0/20	1
Blepharitis	0/132	6	0/133	6	0/19	1	0/21	1	0/48	2	0/50	2	0/731	2	9/731 (1.2)	2	0/20	1	0/20	1
Treatment-related blepharitis	0/227	8	0/224	8	0/19	1	0/21	1	0/74	3	0/76	3	-	-	-	-	0/20	1	0/20	1
Moderate ocular symptoms	0/132	6	0/133	6	0/19	1	0/21	1	0/48	2	0/50	2	5/250 (2.0)	1	32/242 (13.2)	1	0/20	1	0/20	1
Severe ocular symptoms	0/132	6	0/133	6	0/19	1	0/21	1	0/166	4	0/183	4	0/302	2	7/397 (1.8)	2	0/114	2	0/114	2
Ocular infection	1/177 (0.6)	7	1/177 (0.6)	7	0/19	1	0/21	1	0/48	2	0/50	2	7/1086 (0.6)	1	0/1625	1	0/20	1	0/20	1
Instillation site pain	0/132	6	0/133	6	0/19	1	0/21	1	0/48	2	0/50	2	40/1520 (2.6)	9	224/1955 (11.5)	9	0/20	1	0/20	1
Treatment-related instillation site pain	0/227	8	0/224	8	1/49 (2.0)	2	0/65	2	0/74	3	0/76	3	8/184 (4.4)	3	50/434 (11.5)	3	0/20	1	0/20	1
Treatment-related mild instillation site pain	0/227	8	0/224	8	0/19	1	0/21	1	0/74	3	0/76	3	4/90 (4.4)	1	26/154 (16.9)	1	0/20	1	0/20	1
Treatment-related moderate instillation site pain	0/227	8	0/224	8	0/19	1	0/21	1	0/74	3	0/76	3	2/90 (2.2)	1	13/154 (8.4)	1	0/20	1	0/20	1
Treatment-related severe instillation site pain	0/227	8	0/224	8	0/19	1	0/21	1	0/168	4	0/170	4	2/142 (1.4)	2	6/309 (1.9)	2	0/114	2	0/114	2
Ocular discomfort	0/132	6	0/133	6	0/19	1	0/21	1	0/48	2	0/50	2	2/462 (0.4)	2	6/462 (1.3)	2	0/20	1	0/20	1
Mild ocular discomfort	0/132	6	0/133	6	0/19	1	0/21	1	0/48	2	0/50	2	-	-	-	-	0/20	1	0/20	1
Treatment-related ocular discomfort	0/227	8	0/224	8	0/19	1	0/21	1	2/98 (2.0)	4	0/121	4	52/340 (15.3)	3	150/517 (29.0)	3	0/20	1	0/20	1
Dry eye	0/132	6	0/133	6	0/84	2	2/90 (2.2)	2	0/48	2	0/50	2	2/359 (0.6)	1	2/359 (0.6)	1	0/20	1	0/20	1
Eyelid edema	0/132	6	0/133	6	0/19	1	0/21	1	0/48	2	0/50	2	0/359	1	3/359 (0.8)	1	0/20	1	0/20	1
Treatment-related eyelid edema	0/227	8	0/224	8	0/19	1	0/21	1	0/74	3	0/76	3	-	-	-	-	0/20	1	0/20	1
Treatment-related instillation site edema	0/227	8	0/224	8	0/19	1	0/21	1	0/74	3	0/76	3	-	-	-	-	0/20	1	0/20	1
Photophobia	0/132	6	0/133	6	0/19	1	0/21	1	0/48	2	0/50	2	0/359	1	4/359 (1.1)	1	0/20	1	0/20	1
Treatment-related photophobia	0/227	8	0/224	8	0/19	1	0/21	1	0/74	3	0/76	3	-	-	-	-	0/20	1	0/20	1

Corneal erosion	1/148 (0.7)	7	0/149	7	0/19	1	0/21	1	0/48	2	0/50	2	1/609 (0.2)	2	1/601 (0.2)	2	0/20	1	0/20	1
Eye disorders	0/132	6	0/133	6	0/19	1	0/21	1	0/48	2	0/50	2	66/541 (12.2)	3	117/693 (16.9)	3	0/20	1	0/20	1
Visual impairment	0/132	6	0/133	6	0/19	1	0/21	1	0/48	2	0/50	2	0/359	1	1/359 (0.3)	1	0/20	1	0/20	1
Corneal abrasion	0/132	6	0/133	6	0/19	1	0/21	1	0/48	2	0/50	2	4/511 (0.8)	2	2/662 (0.3)	2	0/20	1	0/20	1
Ocular irritation	0/132	6	0/133	6	0/19	1	0/21	1	0/48	2	0/50	2	-	-	-	-	0/20	1	0/20	1

Table S4. Ocular AEs not included in the meta-analysis in patients treated with artificial tears (AT), anti-inflammatory, or immunomodulatory drugs. Abbreviations: IOP, intraocular pressure. Symbol legend: * percentage indicated only for values other than zero.

Event	Anti-inflammatory drugs vs. AT				Immunomodulatory drugs vs. AT			
	Control		Intervention		Control		Intervention	
	N. of events/ patients (%*)	N. of studies	N. of events/ patients (%*)	N. of studies	N. of events/ patients (%*)	N. of studies	N. of events/ patients (%*)	N. of studies
Mild ocular AEs	0/19	1	0/19	1	0/95	2	0/97	2
Moderate ocular AEs	0/19	1	0/19	1	0/95	2	0/97	2
Severe ocular AEs	0/19	1	0/19	1	1/529 (0.2)	4	2/538 (0.4)	4
Serious ocular AEs	0/89	3	0/82	3	0/449	5	0/458	5
Mild ocular treatment-related AEs	0/57	3	0/69	3	0/95	2	0/97	2
Moderate ocular treatment-related AEs	0/57	3	0/69	3	0/95	2	0/97	2
Severe treatment-related ocular AEs	0/57	3	0/69	3	0/215	3	0/217	3
Serious treatment-related ocular AEs	0/127	5	0/132	5	0/693	7	0/700	7
Discontinuation due to ocular AEs	0/19	1	0/19	1	0/125	3	0/127	3

Discontinuation due to intense burning sensation	0/19	1	0/19	1	0/173	4	9/161 (5.6)	4
Discontinuation due to instillation site pain	0/19	1	0/19	1	0/125	3	0/127	3
Discontinuation due to discomfort upon instillation	0/19	1	0/19	1	0/125	3	0/127	3
Discontinuation due to mild infection	0/19	1	0/19	1	0/125	3	0/127	3
Withdrawal due to ocular AEs	0/19	1	0/19	1	0/255	5	0/263	5
Withdrawal due to intolerable drug irritation	0/79	2	0/74	2	0/255	5	0/263	5
Visual acuity reduced	0/19	1	0/19	1	0/115	3	0/117	3
Mild instillation site reactions	0/19	1	0/19	1	0/95	2	0/97	2
Moderate instillation site reactions	0/19	1	0/19	1	0/95	2	0/97	2
Severe instillation site reactions	0/19	1	0/19	1	0/215	3	0/217	3
Blurred vision	5/49 (10.2)	2	2/49 (4.1)	2	5/669 (0.8)	7	3/678 (0.4)	7
Treatment-related blurred vision	0/57	3	0/69	3	0/95	2	0/97	2
Vitreous floaters	0/19	1	0/19	1	0/95	2	0/97	2
Vitreous detachment	0/19	1	0/19	1	0/95	2	0/97	2
Changes in IOP	0/49	2	1/49 (2.0)	2	0/95	2	0/97	2
Meibomian gland dysfunction	0/19	1	0/19	1	0/95	2	0/97	2
Treatment-related grittiness	0/57	3	0/69	3	0/95	2	0/97	2
Treatment-related epiphora	0/57	3	0/69	3	0/95	2	0/97	2
Conjunctivitis	0/19	1	0/19	1	2/339 (0.6)	4	0/339	4

Conjunctival redness	0/19	1	0/19	1	0/95	2	0/97	2
Conjunctival hemorrhage	0/19	1	0/19	1	0/339	4	1/339 (0.3)	4
Conjunctival edema	0/19	1	0/19	1	0/95	2	0/97	2
Eyelid irritation	0/19	1	0/19	1	0/95	2	0/97	2
Eyelid pain	0/19	1	0/19	1	0/95	2	0/97	2
Burning	0/19	1	0/19	1	1/111 (0.9)	4	5/115 (4.4)	4
Burning/ stinging	8/49 (16.3)	2	3/49 (6.1)	2	0/95	2	0/97	2
Treatment-related burning	0/57	3	0/69	3	0/95	2	0/97	2
Treatment-related stinging	0/57	3	0/69	3	0/95	2	0/97	2
Itching	0/19	1	0/19	1	2/111 (1.8)	4	4/115 (3.5)	4
Treatment-related itching	0/57	3	0/69	3	0/95	2	0/97	2
Pingueculitis	0/19	1	0/19	1	0/95	2	0/97	2
Blepharospasm	0/19	1	0/19	1	0/95	2	0/97	2
Episcleritis	0/19	1	0/19	1	0/95	2	0/97	2
Ocular pain/ eye pain	0/49	2	1/49 (2.0)	2	9/653 (1.4)	5	40/660 (6.1)	5
Mild ocular pain	0/19	1	0/19	1	0/215	3	2/217 (0.9)	3
Moderate ocular pain	0/19	1	0/19	1	0/215	3	2/217 (0.9)	3
Severe ocular pain	0/19	1	0/19	1	2/215 (0.9)	3	0/217	3
Treatment-related ocular pain/eye pain	0/57	3	0/69	3	0/95	2	0/97	2
Eye irritation	0/19	1	0/19	1	1/673 (0.2)	6	11/680 (1.6)	6
Treatment-related eye irritation	0/57	3	0/69	3	0/95	2	0/97	2
Treatment-related ocular discomfort	0/57	3	0/69	3	0/95	2	0/97	2
Vision loss	0/19	1	0/19	1	6/409 (1.5)	3	4/418 (1.0)	3
Abnormal sensation in eye	0/19	1	0/19	1	0/339	4	1/339 (0.3)	4
Asthenopia	0/19	1	0/19	1	0/339	4	1/339 (0.3)	4
Treatment-related redness	0/57	3	0/69	3	0/95	2	0/97	2

Eye pruritus	0/19	1	0/19	1	0/339	4	1/339 (0.3)	4
Treatment-related eye pruritus	0/57	3	0/69	3	0/95	2	0/97	2
Instillation site pruritus	0/19	1	0/19	1	0/95	2	0/97	2
Instillation site discomfort	0/19	1	0/19	1	0/95	2	0/97	2
Eye discharge	0/49	2	1/49 (2.0)	2	0/339	4	1/339 (0.3)	4
Treatment-related eye discharge	0/57	3	0/69	3	0/95	2	0/97	2
Foreign body sensation	0/19	1	0/19	1	6/653 (0.9)	5	7/660 (1.1)	5
Treatment-related foreign body sensation	0/57	3	0/69	3	0/95	2	0/97	2
Instillation site reaction	0/19	1	0/19	1	0/95	2	0/97	2
Treatment-related instillation site reaction	0/57	3	0/69	3	0/95	2	0/97	2
Instillation site irritation	0/19	1	0/19	1	0/95	2	0/97	2
Treatment-related instillation site irritation	0/57	3	0/69	3	0/95	2	0/97	2
Treatment-related visual disturbance	0/57	3	0/69	3	0/95	2	0/97	2
Increased lacrimation	0/19	1	0/19	1	3/425 (0.7)	5	6/436 (1.4)	5
Treatment-related increased lacrimation	0/57	3	0/69	3	0/95	2	0/97	2
Treatment-related eyelid erythema	0/57	3	0/69	3	0/95	2	0/97	2
Treatment-related meibomianitis	0/57	3	0/69	3	0/95	2	0/97	2
Conjunctival hyperemia	0/19	1	0/19	1	0/95	2	0/97	2
Mild conjunctival hyperemia	1/65 (1.5)	2	3/67 (4.5)	2	0/95	2	0/97	2
Treatment-related conjunctival hyperemia	0/57	3	0/69	3	0/95	2	0/97	2
Ocular hyperemia	0/19	1	0/19	1	0/339	4	2/339 (0.6)	4

Blepharitis	0/19	1	0/19	1	0/339	4	1/339 (0.3)	4
Treatment-related blepharitis	0/57	3	0/69	3	0/95	2	0/97	2
Moderate ocular symptoms	0/19	1	0/19	1	0/95	2	0/97	2
Severe ocular symptoms	0/19	1	0/19	1	0/95	2	0/97	2
Ocular infection	0/49	2	1/49 (2.0)	2	0/127	3	0/129	3
Instillation site pain	0/19	1	0/19	1	0/95	2	0/97	2
Treatment-related instillation site pain	0/57	3	0/69	3	0/95	2	0/97	2
Treatment-related mild instillation site pain	0/57	3	0/69	3	0/95	2	0/97	2
Treatment-related moderate instillation site pain	0/57	3	0/69	3	0/95	2	0/97	2
Treatment-related severe instillation site pain	0/57	3	0/69	3	0/95	2	0/97	2
Ocular discomfort	0/19	1	0/19	1	9/789 (1.1)	8	11/798 (1.4)	8
Mild ocular discomfort	0/19	1	0/19	1	2/125 (1.6)	3	3/127 (2.4)	3
Dry eye	0/19	1	0/19	1	2/409 (0.5)	3	4/418 (1.0)	3
Eyelid edema	0/19	1	0/19	1	0/95	2	0/97	2
Treatment-related eyelid edema	0/57	3	0/69	3	0/95	2	0/97	2
Treatment-related instillation site edema	0/57	3	0/69	3	0/95	2	0/97	2
Photophobia	0/49	2	2/49 (4.1)	2	0/95	2	0/97	2
Treatment-related photophobia	0/57	3	0/69	3	0/95	2	0/97	2
Corneal erosion	0/19	1	0/19	1	0/95	2	0/97	2
Eye disorders	0/19	1	0/19	1	0/95	2	0/97	2
Visual impairment	0/19	1	0/19	1	0/95	2	0/97	2

Corneal abrasion	0/19	1	0/19	1	0/95	2	0/97	2
Ocular irritation	0/19	1	0/19	1	0/95	2	0/97	2

Table S5. Ocular AEs not included in the meta-analysis in patients treated with a combination of anti-inflammatory or immunomodulatory drugs and artificial tears (AT). Abbreviations: IOP, intraocular pressure. Symbol legend: * percentage indicated only for values other than zero.

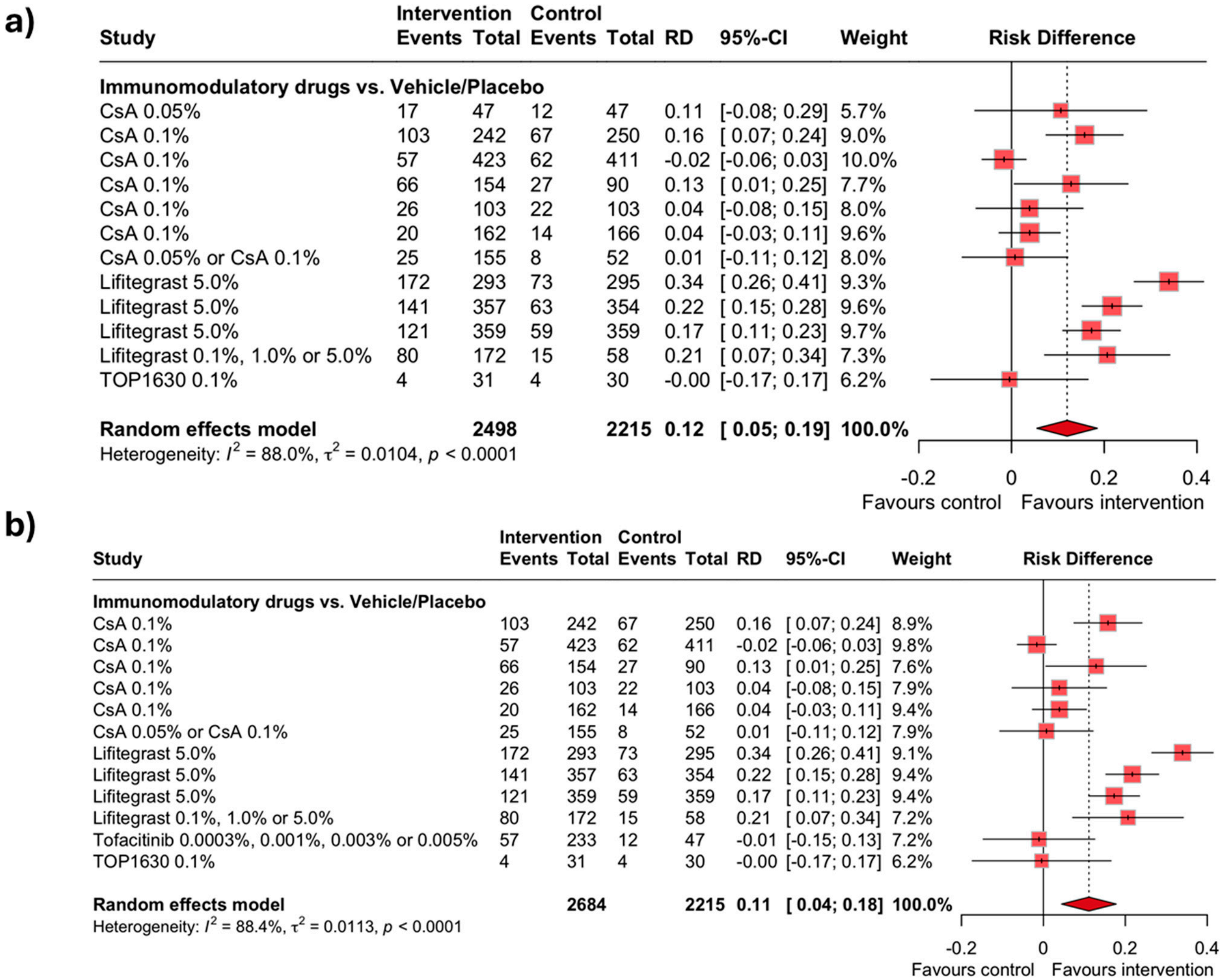

Figure S44. Forest plot of the risk difference (RD) with 95% Confidence Interval (CI) for any ocular AEs in studies comparing immunomodulatory drugs with vehicle or placebo. a) Analysis including one of the two intervention arms that shared the same control group; b) analysis including the other intervention arm. Other abbreviations: CsA, cyclosporine.

Study	Intervention Events	Intervention Total	Control Events	Control Total	RD	95%-CI	Weight
**Immunomodulatory drugs + AT vs. AT**							
CsA 0.05%	1	20	0	20	0.05	[-0.08; 0.18]	6.2%
CsA 0.05%	0	22	0	20	0.00	[-0.09; 0.09]	12.0%
CsA 0.05%	63	314	46	321	0.06	[-0.00; 0.12]	22.9%
CsA 2.0%	3	30	2	30	0.03	[-0.11; 0.17]	5.3%
Chloroquine 0.03%	0	75	0	75	0.00	[-0.03; 0.03]	53.6%
**Random effects model**		**461**		**466**	**0.02**	**[-0.02; 0.05]**	**100.0%**

Heterogeneity: $I^2 = 0.0\%$, $\tau^2 = 0.0004$, $p = 0.4599$

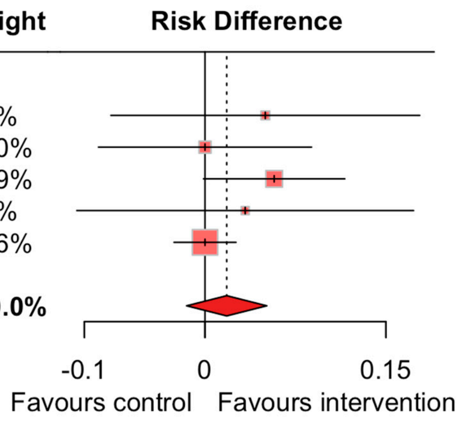

Figure S45. Forest plot of the risk difference (RD) with 95% Confidence Interval (CI) for any ocular AEs in studies comparing a combination of immunomodulatory drugs and artificial tears (AT) with AT alone. Other abbreviations: CsA, cyclosporine.

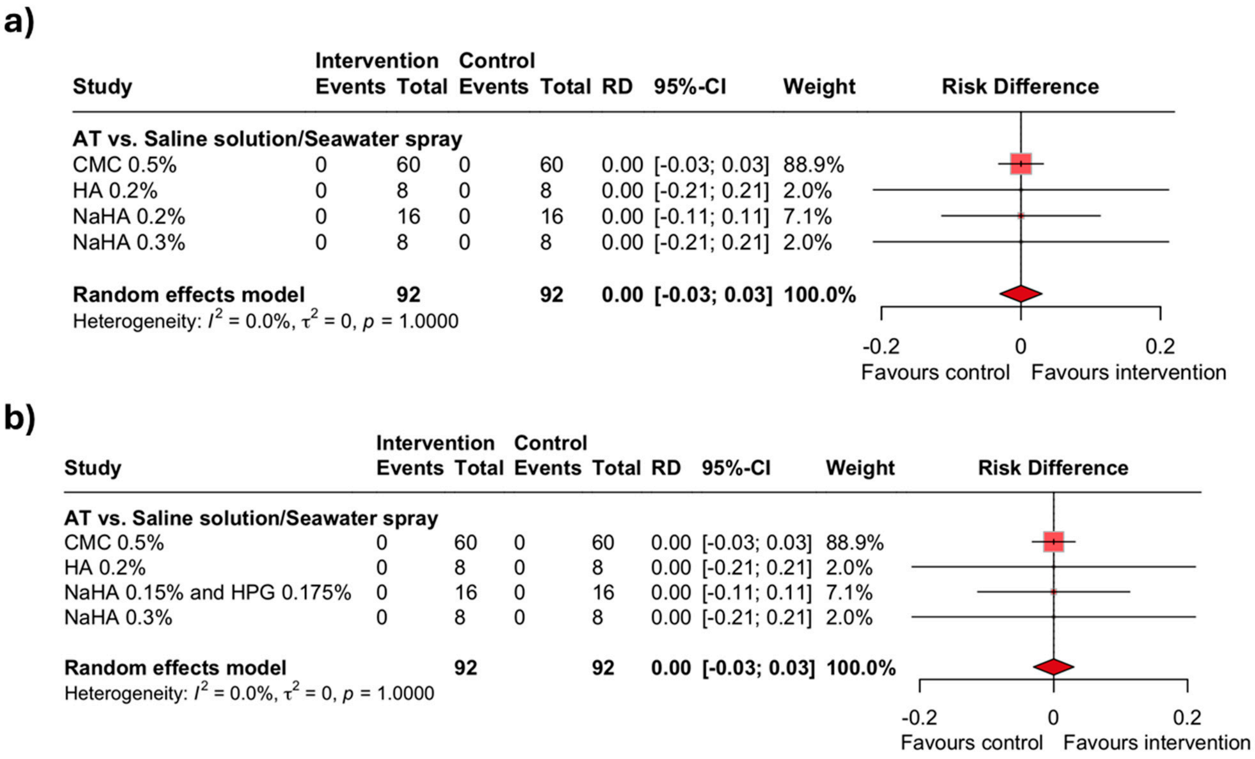

Figure S46. Forest plot of the risk difference (RD) with 95% Confidence Interval (CI) for treatment-related ocular AEs in studies comparing artificial tears (AT) with saline solution or seawater spray. a) Analysis including one of the two intervention arms that shared the same control group; b) analysis including the other intervention arm. Other abbreviations: CMC, carboxymethylcellulose; HA, hyaluronic acid; HPG, hydroxypropyl guar; NaHA, sodium hyaluronate.

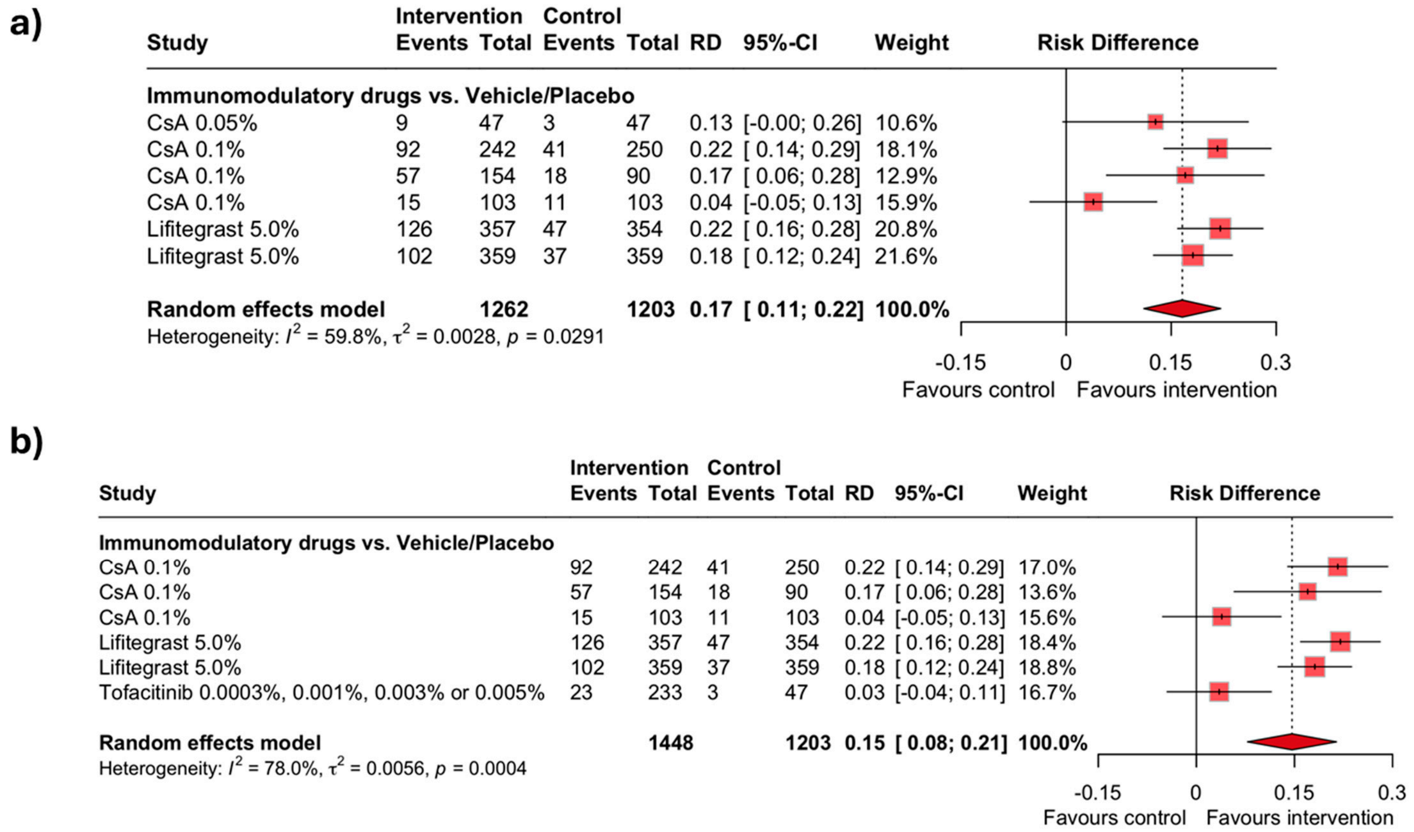

Figure S47. Forest plot of the risk difference (RD) with 95% Confidence Interval (CI) for treatment-related ocular AEs in studies comparing immunomodulatory drugs with vehicle or placebo. a) Analysis including one of the two intervention arms that shared the same control group; b) analysis including the other intervention arm. Other abbreviations: CsA, cyclosporine.

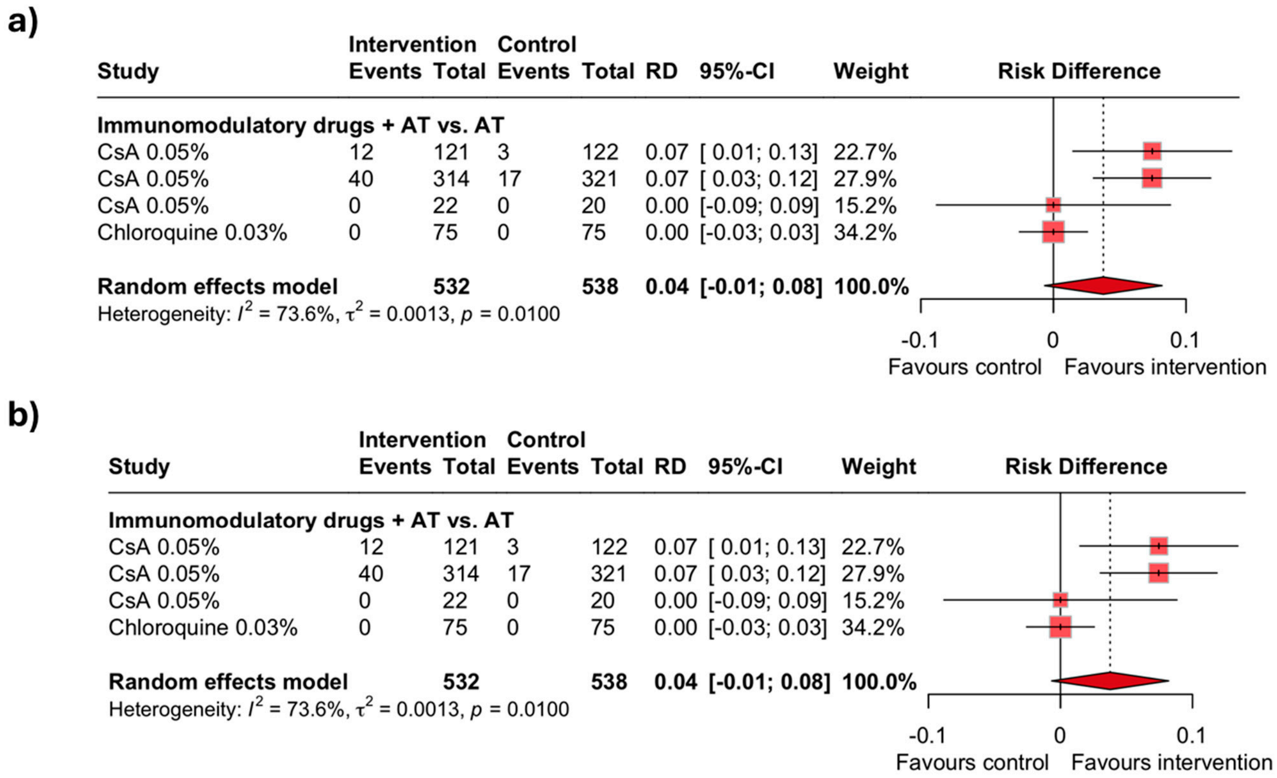

Figure S48. Forest plot of the risk difference (RD) with 95% Confidence Interval (CI) for treatment-related ocular AEs in studies comparing a combination of immunomodulatory drugs and artificial tears (AT) with AT alone. a) Analysis including one of the two intervention arms that shared the same control group; b) analysis including the other intervention arm. Other abbreviations: CsA, cyclosporine.
